# Dual Action of Eeyarestatin 24 on Sec-Dependent Protein Secretion and Bacterial DNA

**DOI:** 10.1021/acsinfecdis.2c00404

**Published:** 2023-01-13

**Authors:** Ann-Britt Schäfer, Maurice Steenhuis, Kin Ki Jim, Jolanda Neef, Sarah O’Keefe, Roger C. Whitehead, Eileithyia Swanton, Biwen Wang, Sven Halbedel, Stephen High, Jan Maarten van Dijl, Joen Luirink, Michaela Wenzel

**Affiliations:** † Division of Chemical Biology, Department of Life Sciences, Chalmers University of Technology, 412 96 Gothenburg, Sweden; ‡ Molecular Microbiology, Amsterdam Institute of Molecular and Life Sciences (AIMMS), Vrije Universiteit Amsterdam, 1081 HV Amsterdam, The Netherlands; § Department of Medical Microbiology and Infection Prevention, Amsterdam University Medical Centers - Location Vrije Universiteit Amsterdam, 1081 HZ Amsterdam, The Netherlands; ∥ Amsterdam Institute for Infection and Immunity, Amsterdam University Medical Centers, 1081 HZ Amsterdam, The Netherlands; ⊥ Department of Medical Microbiology, University of Groningen, University Medical Center Groningen, Hanzeplein 1, PO Box 30001, 9700 RB Groningen, The Netherlands; # School of Biological Sciences, Faculty of Biology, Medicine and Health, University of Manchester, Manchester M13 9PL, United Kingdom; △ School of Chemistry, Faculty of Science and Engineering, University of Manchester, Manchester M13 9PL, United Kingdom; ■ Bacterial Cell Biology and Physiology, Swammerdam Institute for Life Sciences, University of Amsterdam, 1098 XH Amsterdam, The Netherlands; ○ FG11 Division of Enteropathogenic Bacteria and Legionella, Robert Koch Institute, 38855 Wernigerode, Germany; ¶ Institute for Medical Microbiology and Hospital Hygiene, Otto von Guericke University Magdeburg, 39120 Magdeburg, Germany

**Keywords:** antibiotic, mechanism of action, protein secretion, *in vivo* efficacy, nitrofurantoin, eeyarestatin 24

## Abstract

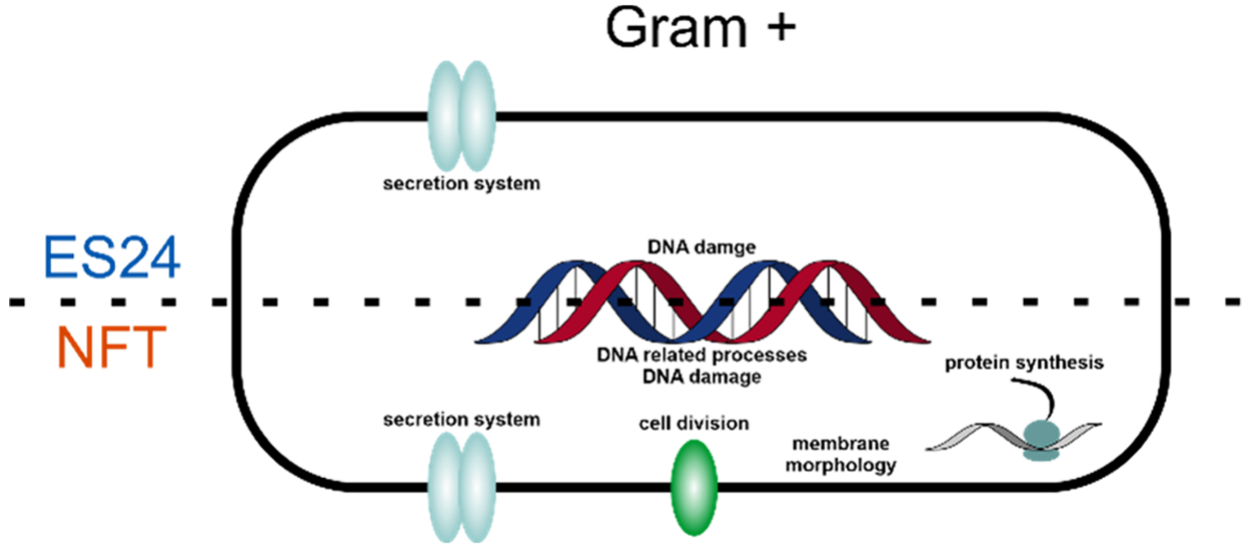

Eeyarestatin 24 (ES24) is a promising new antibiotic with broad-spectrum activity. It shares structural similarity with nitrofurantoin (NFT), yet appears to have a distinct and novel mechanism: ES24 was found to inhibit SecYEG-mediated protein transport and membrane insertion in Gram-negative bacteria. However, possible additional targets have not yet been explored. Moreover, its activity was notably better against Gram-positive bacteria, for which its mechanism of action had not yet been investigated. We have used transcriptomic stress response profiling, phenotypic assays, and protein secretion analyses to investigate the mode of action of ES24 in comparison with NFT using the Gram-positive model bacterium *Bacillus subtilis* and have compared our findings to Gram-negative *Escherichia coli*. Here, we show the inhibition of Sec-dependent protein secretion in *B. subtilis* and additionally provide evidence for DNA damage, probably caused by the generation of reactive derivatives of ES24. Interestingly, ES24 caused a gradual dissipation of the membrane potential, which led to delocalization of cytokinetic proteins and subsequent cell elongation in *E. coli.* However, none of those effects were observed in *B. subtilis*, thereby suggesting that ES24 displays distinct mechanistic differences with respect to Gram-positive and Gram-negative bacteria. Despite its structural similarity to NFT, ES24 profoundly differed in our phenotypic analysis, which implies that it does not share the NFT mechanism of generalized macromolecule and structural damage. Importantly, ES24 outperformed NFT *in vivo* in a zebrafish embryo pneumococcal infection model. Our results suggest that ES24 not only inhibits the Sec translocon, but also targets bacterial DNA and, in Gram-negative bacteria, the cell membrane.

Antimicrobial resistance is a major threat to public health, with some countries reporting resistance rates as high as 92.9% against commonly used antibiotics.^1^ Both Gram-positive and Gram-negative bacteria contribute to this problem,^2^ yet the development of innovative drugs, especially with broad-spectrum activity, is still lacking. In particular, the number of antibiotics in clinical development that belong to truly novel structural or mechanistic classes is limited.^3^ Considerable efforts have been undertaken to find such new classes, and the preclinical end of the pipeline has been filled with compounds with untapped mechanisms.^4^ While it is promising that a number of these drug candidates target Gram-negative bacteria, which are difficult to treat because of their highly impermeable outer membrane, only a few have broad-spectrum activity.^4^

Eeyarestatin 24 (ES24) is a recently discovered small molecule with broad-spectrum antibacterial activity and a novel mechanism of action. It is derived from eeyarestatin 1 (ES1), a compound that was first discovered in a high-throughput screen for inhibitors of the mammalian endoplasmic reticulum (ER)-to-cytosol degradation pathway for the disposal of misfolded proteins in a quest to find new therapeutics for diseases that are characterized by protein degradation defects.^5,6^ ES1 inhibits two major ER-based cellular processes: the ER-associated degradation (ERAD) of misfolded proteins^7−10^ and the Sec61-dependent translocation of nascent polypeptides into the ER.^11−13^ Recently, a cryogenic electron microscopy (cryo-EM) structure of Sec61 in complex with ES1 has been solved.^14^ ES24 is a smaller derivative of ES1, which retains its activity against Sec61 but not the ERAD system.^11^ Since the eukaryotic Sec61 complex is homologous to the bacterial SecYEG translocon,^15^ ES1 and ES24 have also sparked interest as potential inhibitors of bacterial protein secretion, a pathway that is essential in both Gram-positive and Gram-negative bacteria but has not yet been exploited as an antibiotic target.^16−19^

While ES1 was unable to cross the Gram-negative outer membrane and exhibited significant mammalian cell toxicity, ES24 showed broad-spectrum antibacterial activity and was significantly less toxic to HEK293 cells.^11,16^ It was recently shown using the Gram-negative *Escherichia coli* as a model system that ES24 indeed inhibits SecYEG-dependent protein translocation, both in terms of inner membrane protein insertion and protein secretion into the periplasm. In line with this observation, stress response profiling revealed an activation of the σ^32^-dependent heat shock response.^16^

ES24 contains a nitrofuran group and, thus, shares structural similarity with the long-established antibiotic nitrofurantoin (NFT) (Figure 1A), which is commonly prescribed for the treatment of urinary tract infections.^20^ Despite being widely used since 1953, resistance against this drug is relatively uncommon.^20^ NFT is a prodrug. Upon activation by bacterial nitroreductases, reactive derivatives are formed that damage cellular components, in particular, the DNA and cell membrane, in a manner similar to oxidative stress.^21−24^ Interestingly, ES24 also requires the presence of the nitroreductases NsfA and NsfB to inhibit the growth of *E. coli*, which suggests that it undergoes a similar activation process.^16^ Notably, ES24 displayed higher activity than NFT against different Gram-positive and Gram-negative test strains.^16^ Comparative stress response profiling revealed a significant overlap between the two compounds, in particular, activation of SOS and oxidative stress responses, yet nitrofurantoin had no effect on SecYEG-dependent protein translocation.^16^ This raised the question of whether the activation of ES24 by cellular nitroreductases may also result in reactive compounds that cause cellular damage, in addition to its inhibitory effect on protein secretion. Moreover, both ES24 and NFT have led to cell elongation, thereby indicating potential additional or downstream effects on cell division.^16^

**Figure 1:**
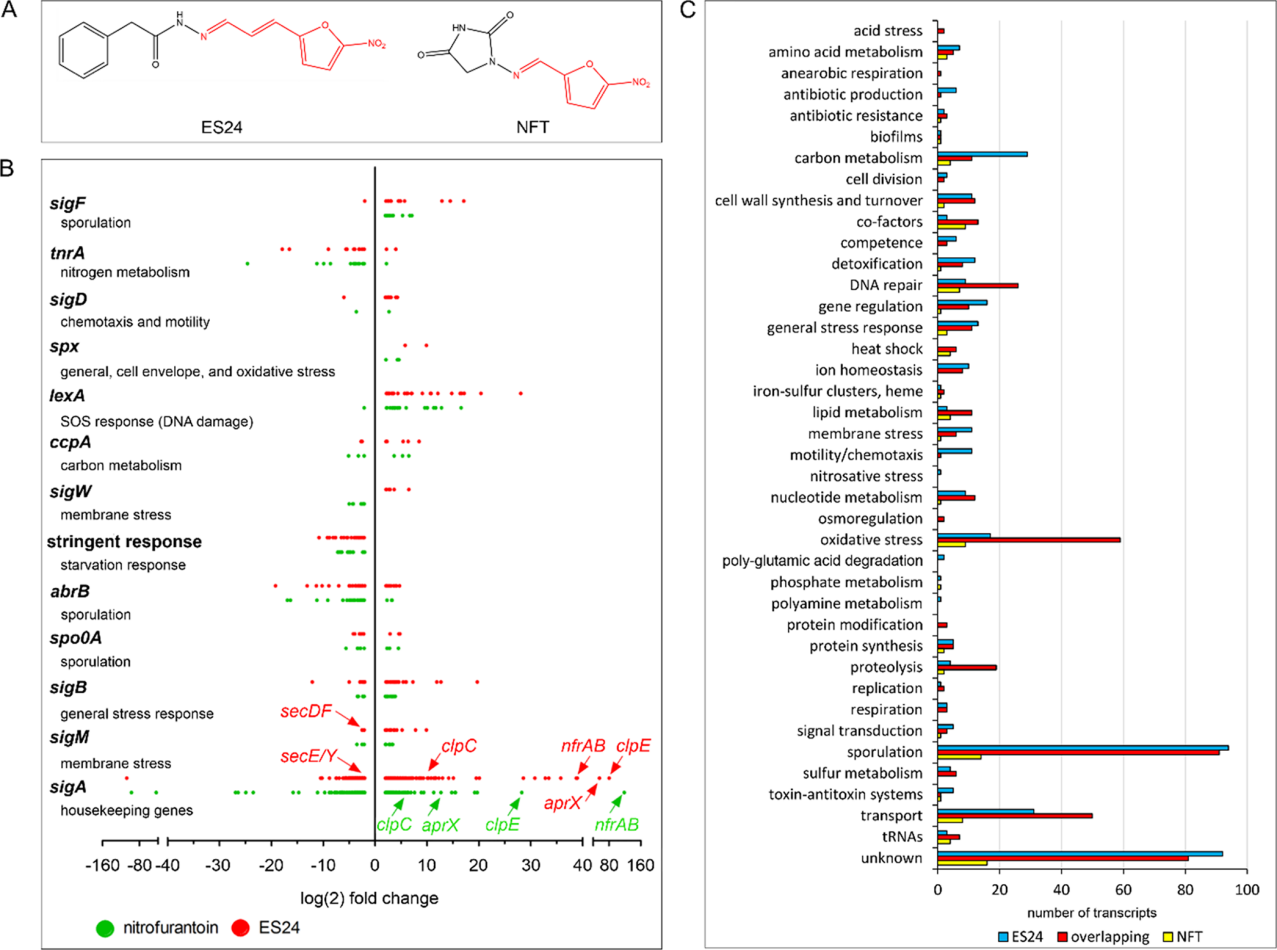
Summary of *B. subtilis* transcriptomic stress profiling results. (A) Chemical structures of ES24 and NFT. The nitrofuran ring is highlighted in red. (B) Number of regulated transcripts per regulon. Sec components and proteases potentially indicating secretion stress are highlighted. NfrAB is a nitro/flavin reductase under transcriptional control of the Spx regulator and among the most strongly activated transcripts for both ES24 and NFT treatment. (C) Number of induced transcripts per functional category of the respective gene products. Functional categories were assigned according to function, regulation, interaction, and expression data available on http://subtiwiki.uni-goettingen.de/. Transcripts that showed a log_2_-fold change of ≥3 (*p* < 0.05) were considered as differentially regulated.

These observations left several open questions, most importantly whether ES24 is truly specific for SecYEG or if it displays any additional activities in bacteria, possibly related to the formation of reactive derivatives or the downstream effects of impaired protein translocation. Moreover, ES24 was notably more active against Gram-positive species [30-fold lower minimal inhibitory concentration (MIC) against *Bacillus subtilis* compared with *E. coli*), but its mechanism of action against this class of bacteria remains to be elucidated. Since Gram-positive bacteria lack a membrane-enclosed periplasm, the effects of secretion inhibition by ES24 may differ from observations made in *E. coli*. Finally, even though ES24 was more active against bacterial pathogens than NFT and less cytotoxic than its parent compound ES1, its *in vivo* efficacy has not yet been assessed.

Here, we characterized the mechanism of action of ES24 in the Gram-positive model organism *B. subtilis* and compared it with NFT. Stress response profiling revealed the activation of oxidative stress, DNA damage, and membrane stress responses. Following up on these different leads, we performed phenotypic analyses using different bacterial cytological profiling techniques. We found that ES24 indeed affects SecYEG-dependent protein translocation in *B. subtilis* but also induces DNA damage. ES24 was notably different from NFT in all assays, which suggests that it does not generate the same reactive derivatives, at least not at concentrations sufficient to inhibit bacterial growth. Strikingly, ES24 outperformed NFT *in vivo* in a zebrafish embryo pneumococcal infection model, thereby highlighting the clinical promise of this compound.

## Results

### Transcriptomic Stress Response Profiling

We started out with transcriptomic stress response profiling as an unbiased global approach. To this end, we first determined suitable antibiotic concentrations for physiological stress experiments in our model strain *B. subtilis* 168CA. Minimal inhibitory concentrations (MICs) against this strain were 0.75 μM for ES24 and 15 μM for NFT. This is similar to previous results reporting ES24 to be 25 times more active against *B. subtilis* than NFT.^16^

Transcriptomics experiments were performed according to the conditions previously used for *E. coli* to achieve optimal comparability.^16^ Thus, cultures were grown to mid-log phase, diluted to an OD_600_ of 0.05, and subsequently treated with 3 μM ES24 or 25 μM NFT for 15 min prior to RNA extraction and sample analysis. Figure 1B,C shows an overview of the transcriptomic data set. Tables S1–S3 summarize all upregulated transcripts. The full raw data sets can be found in the Supporting Information.

We first analyzed the regulons of differentially regulated transcripts, as previously done for *E. coli* (Figure 1B).^16^ This analysis revealed that the major up- and downregulated regulons are rather similar for ES24 and NFT (Figure 1B), which follows the same trend previously observed in *E. coli*.^16^ Affected regulons reflected general and large-scale stress adaptations, such as sporulation, stringent response, general stress response, chemotaxis, and motility. This is characteristic for compounds that affect multiple downstream processes.

Both compounds showed induction of transcripts regulated by LexA, thereby controlling the SOS response to DNA damage, which is consistent with the results obtained for *E. coli*.^16^ While *B. subtilis* did not show a clear heat-shock response, as previously observed in *E. coli*, we did observe the upregulation of proteases, which is consistent with secretion inhibition, yet may also be caused by general stress. Notably, ES24, but not NFT, led to downregulation of Sec components (Figure 1B). Another marked difference was observed regarding the cell envelope stress-responsive σ^W^ regulon, which was induced by ES24 but repressed by NFT.

In *B. subtilis*, a significant number of genes is part of more than one regulatory mechanism. Therefore, we performed a deeper analysis of the functions of upregulated transcripts. To this end, we sorted all transcripts into functional categories by taking into account function, expression, regulation, and interaction data from the SubtiWiki database (www.subtiwiki.uni-goettingen.de)^25^ (Figure 1C, Tables S1–S3). This analysis revealed largely overlapping stress responses to ES24 and NFT, with sporulation-related genes being the largest upregulated group. It also showed DNA repair and proteolysis as major responses to both compounds, while cell envelope stress was more prominent in ES24-treated samples. Additionally, we observed the upregulation of genes encoding membrane transport proteins, which could reflect an attempt to compensate for impaired protein secretion. Similar to other observations, this response was stronger in ES24-treated samples. Interestingly, we also observed that both ES24 and NFT induced a large number of transcripts that are typically found upregulated upon oxidative stress.

Taken together, the transcriptome analysis led us to the following hypotheses: (1) both ES24 and NFT cause the formation of reactive species that elicit an oxidative stress response; (2) both compounds induce DNA damage; (3) ES24 induces membrane stress, possibly because of disturbed protein secretion; and (4) ES24 impairs the Sec translocon. In the following sections, we tested each of these hypotheses with independent assays.

### Antibiotic Concentrations for Phenotypic Analyses

To this end, we used a bacterial cytological profiling approach, making use of different fluorescent protein fusions and dyes that have been successfully employed in previous mode of action studies.^26−28^ We first determined optimal stressor concentrations for these assays by examining the effects of acute antibiotic stress in log phase (Figure S1). We chose concentrations that led to a temporary halt of culture growth but did not result in cell death. This ensures that the stress applied is enough to elicit a phenotype while avoiding effects that are merely a consequence of cell death. These conditions were met at 4 μM for ES24 and 25 μM for NFT (Figure S1). Since the similarity in the transcriptome profiles led us to hypothesize that ES24 could possibly have a dual mechanism of inhibiting protein secretion and generating reactive species, we additionally included a lower concentration of 2 μM to assess any concentration-dependent effects. These concentrations were then used throughout all phenotypic experiments using *B. subtilis*. As additional controls, we used gramicidin (an antibiotic that forms a transmembrane ion channel and consequently perturbs a variety of cellular functions) and, where indicated, hydrogen peroxide (H_2_O_2_, producing hydroxyl radicals) and paraquat (producing superoxide) as controls for oxidative stress.

### Oxidative Stress

**Figure 2:**
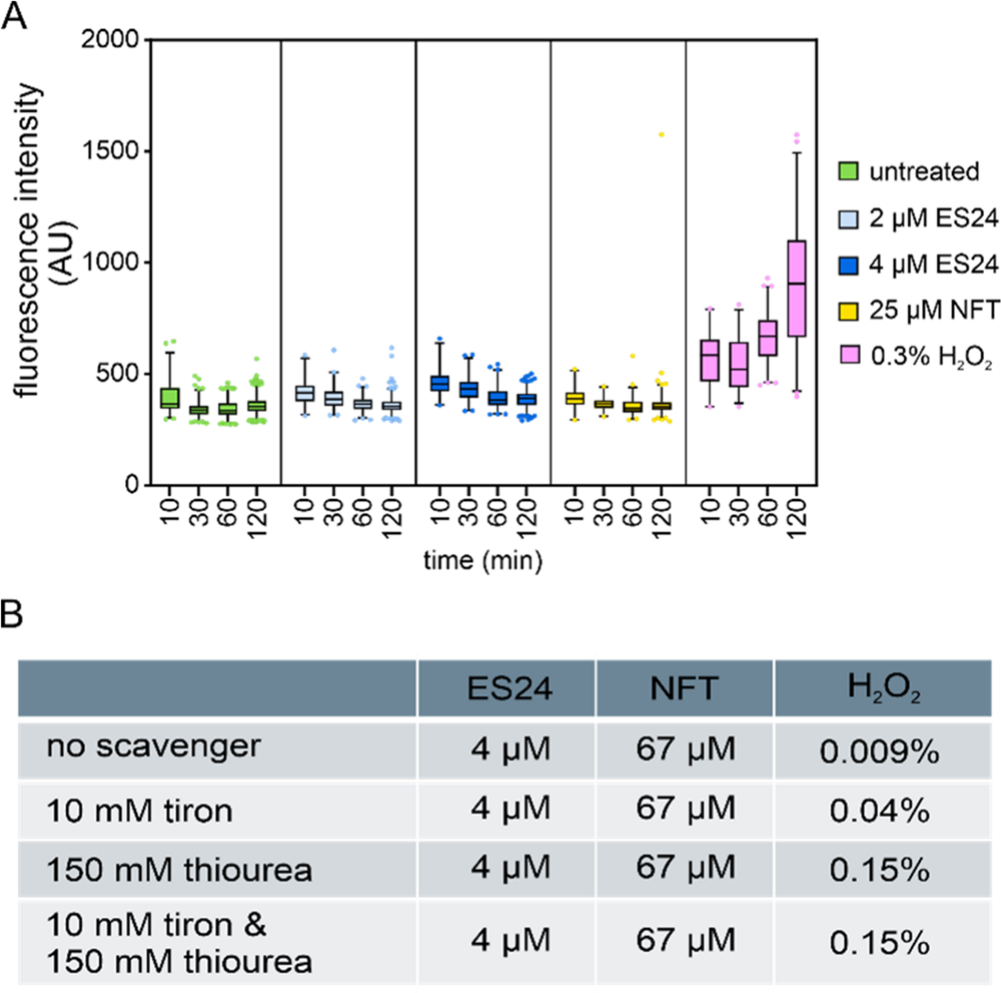
Oxidative stress assays. (A) Oxyburst Green assay for detecting ROS in *B. subtilis* 168CA. The fluorescent probe shows a marked increase in fluorescence when ROS are present. It should be noted that Oxyburst Green has been reported to be specific for the detection of superoxide, yet we have found that it reacts to different sources of ROS (see Figure S2). (B) Influence of ROS scavengers on antibiotic activity against *B. subtilis* 168CA. MICs were determined in the absence or presence of the superoxide scavenger tiron or the hydroxyl radical scavenger thiourea. If one of these ROS species contributes to the activity of the compound, the MIC should increase in the presence of the respective scavenger.

Nitrofurantoin has been suggested to generate reactive oxygen species (ROS),^29^ and both ES24 and NFT induced a high number of transcripts that indicate oxidative stress (Figure 1C) and are typically induced by hydrogen peroxide.^30^ Therefore, we examined whether ROS are present in the antibiotic-treated samples using the fluorescent reporter Oxyburst Green (Figure S2).^31^ However, we did not observe a marked increase in fluorescence signal for any of the samples compared with 0.3% hydrogen peroxide (Figure 2A). To confirm this result, we determined MICs against *B. subtilis* in the absence and presence of the superoxide scavenger tiron and the hydroxyl radical scavenger thiourea.^32,33^ While tiron moderately, and thiourea strongly, increased the MIC of the positive control hydrogen peroxide, neither scavenger affected the MICs of ES24 or NFT (Figure 2B), thereby supporting the notion that no ROS are formed by these antibiotics.

### DNA Damage

**Figure 3:**
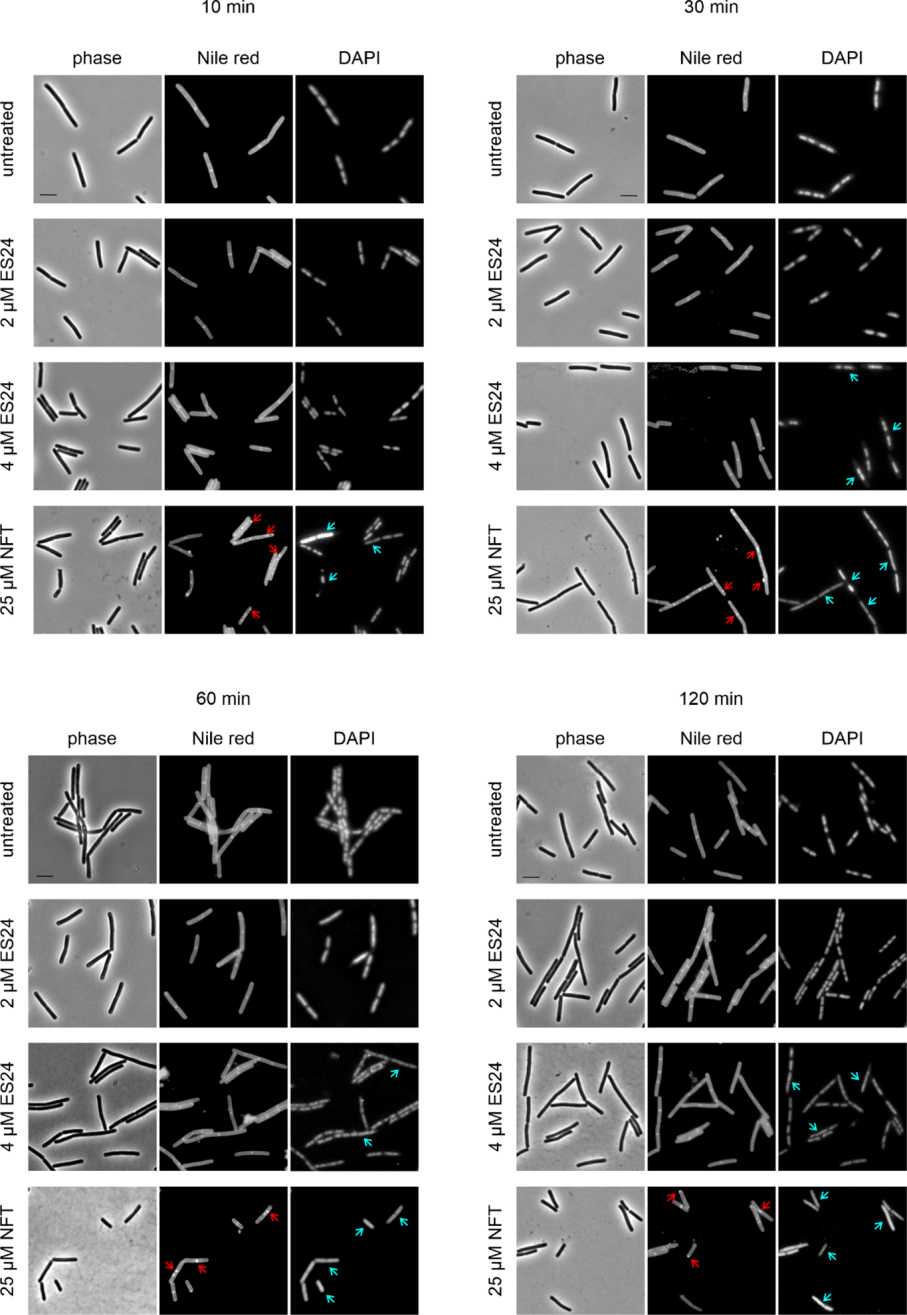
Bacterial cytological profiling of *B. subtilis* 168CA treated with ES24 or NFT. Red arrows indicate membrane aberrations, and blue arrows indicate nucleoid aberrations. See Figure S4 for corresponding images of cells treated with hydrogen peroxide and paraquat. Scale bar, 2 μM.

While we did not find evidence for the presence of ROS, it is possible that nitroreductases metabolize NFT and/or ES24 into other reactive species, for example, nitric oxide or other reactive nitrogen species, that may cause similar cell damage and, hence, elicit a similar stress response without necessarily being detected by ROS-specific probes or scavengers. This hypothesis is supported by the upregulation of DNA damage repair mechanisms, such as the LexA-regulated SOS response, which are typically induced upon oxidative DNA damage (Figure 1B,C). Therefore, we examined the effects of the compounds on bacterial DNA in more detail. We first examined nucleoid morphology with a DAPI stain and used the membrane dye Nile red as a counter stain (Figure 3, Figure S3). It was previously observed that the treatment of *B. subtilis* with high NFT concentrations led to a temporary condensation of the nucleoid followed by an entirely dispersed DAPI signal after 30 min.^21^ Here, despite using lower concentrations of NFT, the same effect was observed, thereby indicating strong effects on nucleoid integrity. ES24 caused similar, yet overall slower and milder, effects. Thus, we observed nucleoid condensation after 30 min followed by relaxation after 60 min, yet no complete dispersion of the DAPI signal. Notably, these phenotypes did not resemble the nucleoid morphology observed after treatment with hydrogen peroxide or paraquat (Figure S4), thereby supporting our notion that ROS do not account for these effects.

**Figure 4:**
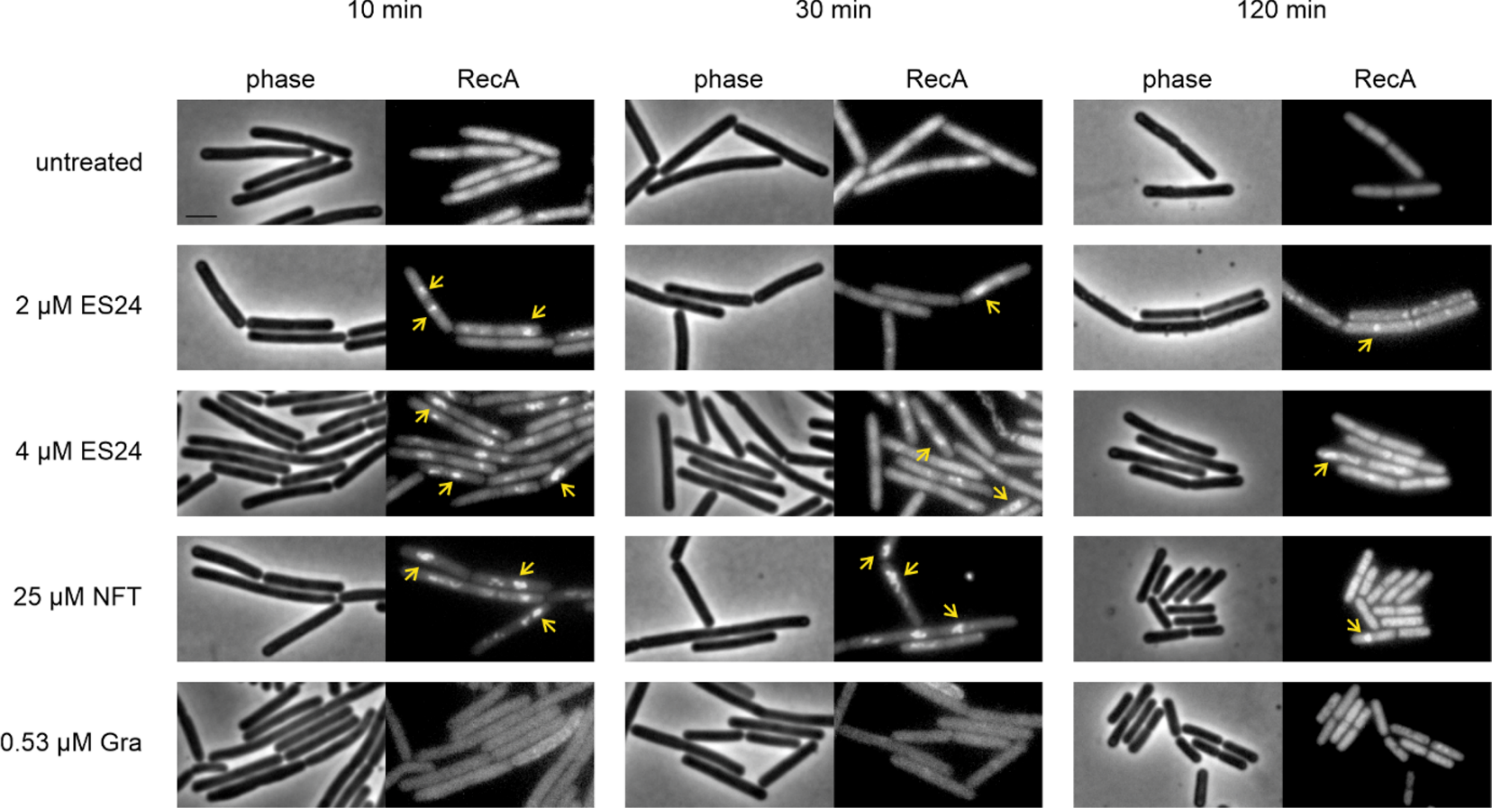
Localization of RecA-GFP (*B. subtilis* UG10) after treatment with ES24 or NFT. Gramicidin (Gra) was used as additional control. Yellow arrows indicate clustered protein. Scale bar, 2 μM.

We verified that the observed effects on nucleoid morphology are indeed indicative of DNA damage by employing a green fluorescent protein (GFP) fusion to the DNA damage reporter RecA (Table 1, Figure 4). This DNA repair protein is uniformly associated with the nucleoid and, upon DNA damage, forms foci over the damaged sites.^34^ Indeed, RecA foci were observed for both ES24 and NFT-treated cells, thereby indicating that both compounds induce DNA damage. While we observed more cells with distinct RecA foci upon treatment with NFT, ES24-treated cells showed a clear, concentration-dependent increase of RecA foci. In both cases, the RecA response was strongest after 10 min of treatment and decreased over time (Figure S5).

**Table 1:** Overview of GFP Localization Results, With the Ion Pore-Forming Peptide Antibiotic Gramicidin (Gra), Which Affects Nearly All GFP Fusion Proteins, Used as a Positive Control^a^

reporter for	protein	localization pattern				
		untreated	0.53 μM Gra^b^	15 μM NFT^c^	2 μM ES24	4 μM ES24
DNA damage	RecA	cytosolic: dispersed	cytosolic: dispersed	**nucleoid-associated foci**^b^	**nucleoid-associated foci**^b^	**nucleoid-associated foci**^b^
replication	DnaN	cytosolic: nucleoid-associated	**cytosolic: dispersed**^b^	**cytosolic: dispersed**^c^	cytosolic: nucleoid-associated	cytosolic: nucleoid-associated
transcription	RpoC	cytosolic: nucleoid-associated	**cytosolic: dispersed**^b^	**cytosolic: dispersed**^b^	cytosolic: nucleoid-associated	cytosolic: nucleoid-associated
translation	RpsB	cytosolic: excluded from nucleoid	**cytosolic: dispersed**^b^	**cytosolic: dispersed**^c^	cytosolic: excluded from nucleoid	cytosolic: excluded from nucleoid
membrane potential, cell division	DivIVA	membrane-associated: septal/polar	**cytosolic: dispersed**^b^	**cytosolic: dispersed, membrane: clusters**^b^	membrane-associated: septal/polar	membrane-associated: septal/polar
membrane potential, cell division	MinD	membrane-associated: septal/polar	**cytosolic: dispersed^b^**	**cytosolic: dispersed, membrane: clusters^c^**	membrane-associated: septal/polar	membrane-associated: septal/polar
ATP synthesis	AtpA	membrane-associated: foci	**cytosolic: dispersed**^b^	**cytosolic: dispersed, membrane: clusters**^c^	membrane-associated: foci	membrane-associated: foci
protein secretion	SecA	membrane-associated: foci	**cytosolic: dispersed**^b^	**cytosolic: dispersed, membrane clusters**^c^	**membrane: clusters**^c^	**membrane: clusters**^c^

a Fusions showing an effect are in bold.

b Immediate effects.

c Effects visible after 30–120 min.

We gained insight into whether this DNA damage and the concomitant changes to nucleoid morphology affect other nucleoid-associated proteins by examining the localization of the DNA polymerase DnaN and the RNA polymerase RpoC. Complementarily, we included the ribosomal protein RpsB, which is excluded from the nucleoid (Table 1, Supplementary Figures S6–S8). While NFT showed strong effects on the localization of all three proteins, ES24 did not show any pronounced phenotype in these assays, which is in line with its milder effects on nucleoid morphology and a less pronounced RecA response (Figure 3 and 4).

### Membrane Stress

Our transcriptomic analysis revealed that ES24 induced more transcripts that are functionally connected to membrane stress than NFT. Yet, none of the common membrane stress markers that are typically induced by membrane-interacting compounds, like gramicidins,^35^ were upregulated by ES24. If ES24 indeed inhibits the Sec translocon in *B. subtilis*, an accumulation of normally secreted proteins in the cell membrane may explain such an “atypical” membrane stress response. If that is, indeed, the case, we should not observe phenotypes typically associated with direct membrane damage. If, however, reactive species are formed that attack the cell membrane, at least some effects should be observed, as recently shown for NFT.^21^ We investigated this by examining membrane parameters that are typically affected by membrane-interacting compounds: membrane morphology, membrane potential, membrane fluidity, and membrane protein localization.

**Figure 5:**
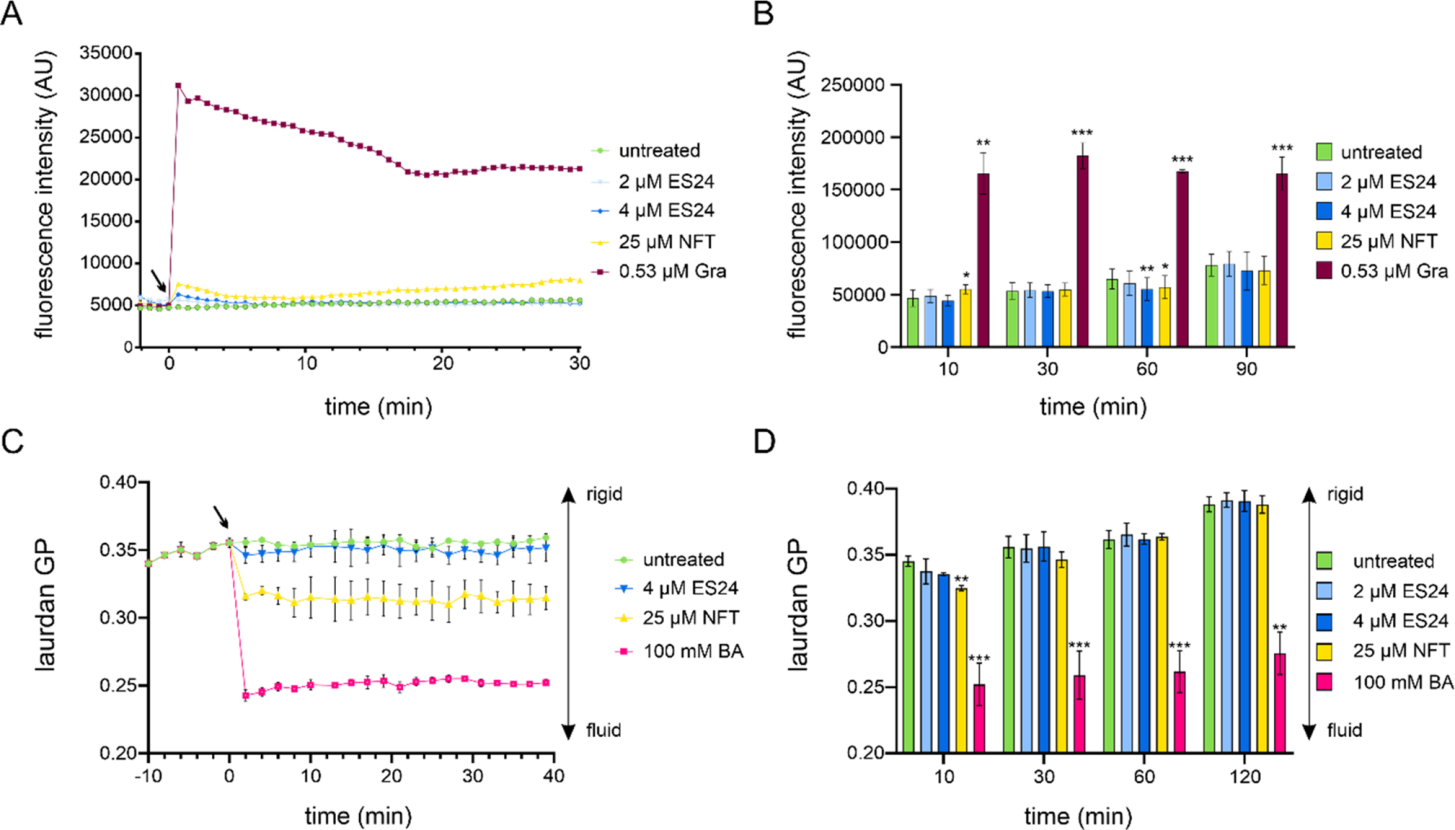
Effects of ES24 and NFT on *B. subtilis* membrane potential and fluidity. (A) Kinetic DiSC(3)5 spectroscopy measurements of *B. subtilis* 168CA treated with ES24, NFT, or positive control gramicidin (Gra). An increase in fluorescence intensity indicates membrane depolarization. The arrow indicates the time point of antibiotic addition. (B) End point measurements under the same conditions as in panel A up to 90 min. (C) Kinetic laurdan generalized polarization (GP) spectroscopy measurements of *B. subtilis* 168CA treated with ES24, NFT, or positive control benzyl alcohol (BA). A decrease in GP indicates membrane fluidization. The arrow indicates the time point of antibiotic addition. (D) End point measurements under the same conditions as in panel C up to 120 min. The *p*-values were calculated using a paired two-tailed *t* test. *p < 0.05, **p < 0.01, ***p < 0.001.

We first examined the Nile red membrane stain for morphological changes of the cell membrane. While ES24 had no visible effect on this membrane stain, NFT caused aberrant membrane foci (Figure 3, Figure S3), which is in line with an earlier study.^21^ Similar membrane defects were observed after treatment with hydrogen peroxide and paraquat (Figure S4), which suggests that they may be due to membrane damage caused by reactive species. Next, we tested whether the compounds affect the membrane potential using the voltage-sensitive dye DiSC(3)5. Neither ES24 nor NFT showed a notable effect in this assay (Figure 5A,B). We then examined the effects of the compounds on membrane fluidity, which has recently emerged as a key factor in the activities of membrane-active antibiotics.^26−28,36,37^ We used the fluidity-sensitive fluorescence dye laurdan^38,39^ and observed no change in membrane fluidity in ES24-treated cells. In contrast, NFT caused a clear membrane fluidization (Figure 5C). However, this effect disappeared during extended treatment, thereby suggesting that cells are able to re-establish their original membrane fluidity (Figure 5D). We hypothesized that this transient fluidization could be a consequence of lipid peroxidation due to reactive species. However, when we measured the membrane fluidity of cells treated with peroxide and paraquat, only very minor effects were observed, which suggests that lipid peroxidation does not lead to a similar fluidization effect (Figure S9). Thus, it is a more likely explanation that the transient fluidization observed with NFT is due to a temporary disturbance of membrane function when the compound crosses the bilayer.

To corroborate our findings, we examined the localization of the peripheral membrane proteins MinD and DivIVA, both of which are involved in cell division site regulation and depend on the membrane potential for correct localization,^28,40^ and AtpA, a subunit of the transmembrane ATP synthase complex that is insensitive to fluctuations in both membrane potential and fluidity.^28^ ES24 did not affect the localization of any of these proteins (Table 1, Figures S10–S12). In contrast, NFT affected all three proteins, with DivIVA delocalizing in a shorter time frame than MinD and AtpA.

### Inhibition of Protein Secretion

**Figure 6:**
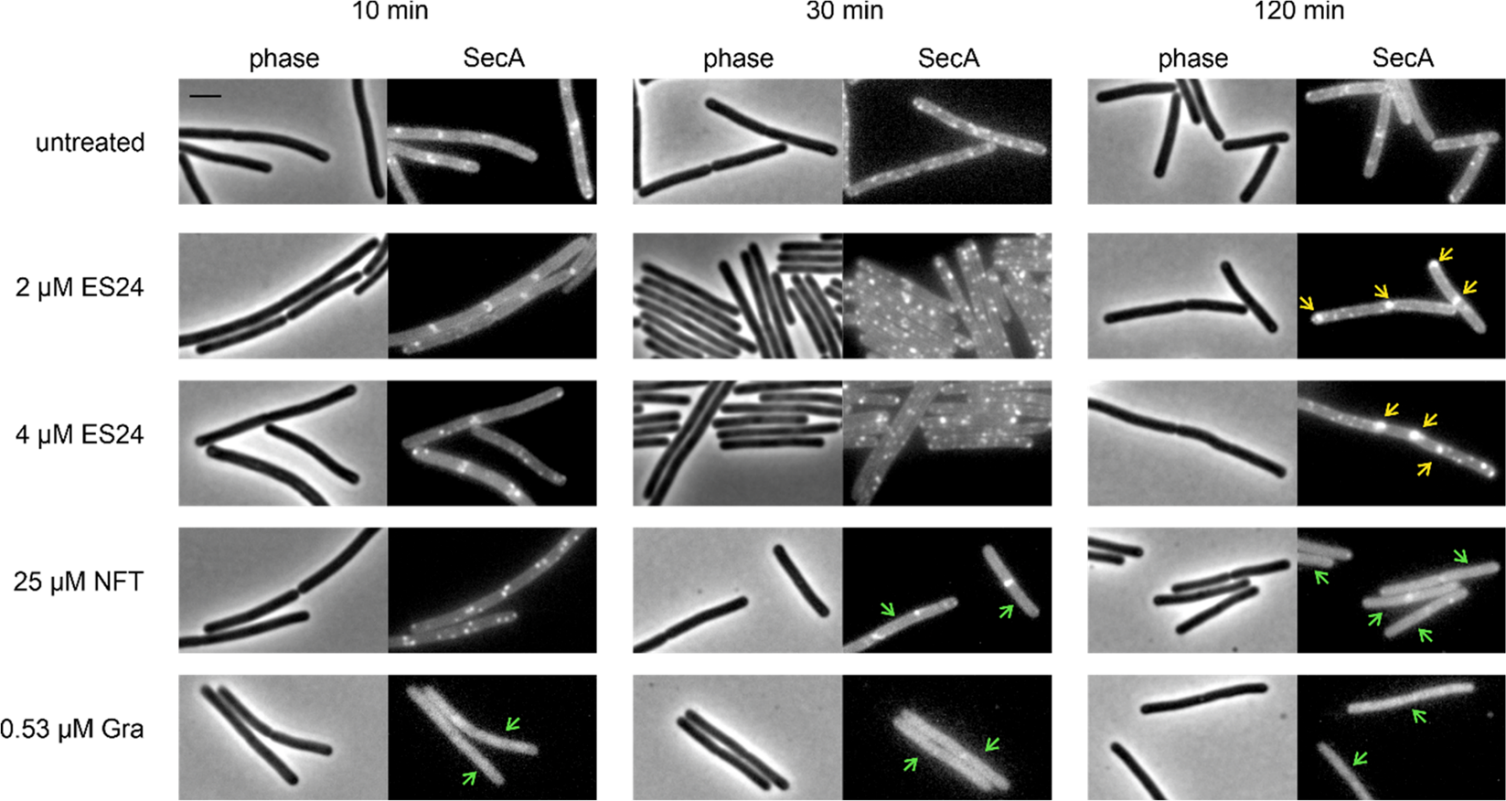
Localization of SecA-GFP after treatment with ES24 or NFT. Gramicidin (Gra) was used as additional control. Yellow arrows: clustered protein. Green arrows: dispersed protein. Scale bar, 2 μM.

To examine whether ES24 inhibits the Sec translocon in *B. subtilis*, we first examined the localization of a GFP fusion to the Sec component SecA. Prolonged treatment with ES24 caused a clear accumulation of SecA at specific sites in the cell membrane (Table 1, Figure 6, Figure S13). While NFT and gramicidin also affected SecA localization, they caused the protein to dissociate into the cytosol, an effect commonly observed for peripheral membrane proteins upon membrane damage.^26−28,41^ The accumulation of SecA at specific sites, as observed with ES24, rather indicates a disturbance of the Sec translocon itself. We excluded that the observed accumulation of SecA may be related to delayed transition into stationary phase in treated cells (Figure S1) by sampling the localization of SecA at different time points over the growth curve of an untreated control culture (Figure S14). While SecA did lose its membrane binding after cells entered the late stationary “death phase,” we did not detect any accumulation into large foci, as observed for ES24-treated cells at any stage during the growth curve.

**Figure 7:**
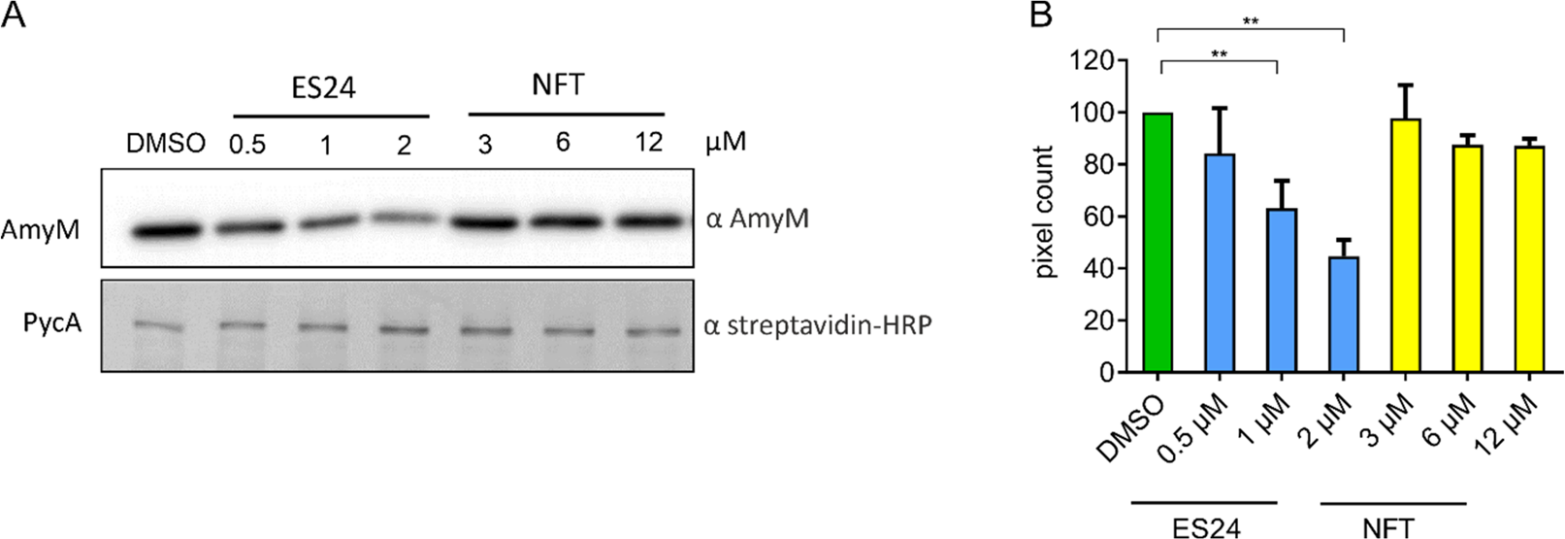
Effect on AmyM secretion. *B. subtilis* BWB09 (*ΔxynA ΔamyE*) carrying pCS73 (*PamyQ–amyM*) to express *amyM* from the constitutive *PamyQ* promoter was grown until mid-log phase and treated with different concentrations of ES24 or NFT for 3 h prior to SDS-PAGE and Western blotting. (A) Western blot analysis of culture supernatant using an α-AmyM antibody (top) and of cell lysate using an α-streptavidin antibody detecting the cytosolic protein PycA (bottom). (B) Quantification of pixel intensity in AmyM bands. **p < 0.01.

We then analyzed the capability of *B. subtilis* cultures to secrete the extracellular amylase AmyM using Western blotting. To this end, we used a *B. subtilis* strain expressing *amyM* from the constitutive *PamyQ* promoter. As shown in Figure 7, ES24 caused a clear, concentration-dependent inhibition of AmyM secretion, while no effect was observed with NFT (see Figures S15 and S16 for additional controls), thereby indicating that ES24 indeed inhibits SecYEG-dependent protein secretion in Gram-positive bacteria. As an additional control, we included the cytosolic protein PycA in the Western blot analysis. Neither ES24 nor NFT affected the levels of this control protein. We corroborated our results by additionally investigating the secretion of LipA, an extracellular lipase that is secreted by the Sec translocon.^42^ Indeed, we observed that 2 μM ES24 caused a 20–30% reduction in LipA secretion compared with bacteria cultured in the absence of ES24 or bacteria treated with only 1 μM ES24. In contrast, NFT at concentrations of up to 12 μM, had no detectable effect on LipA secretion (Figure S17).

### Mechanistic Differences in *E. coli*

**Figure 8:**
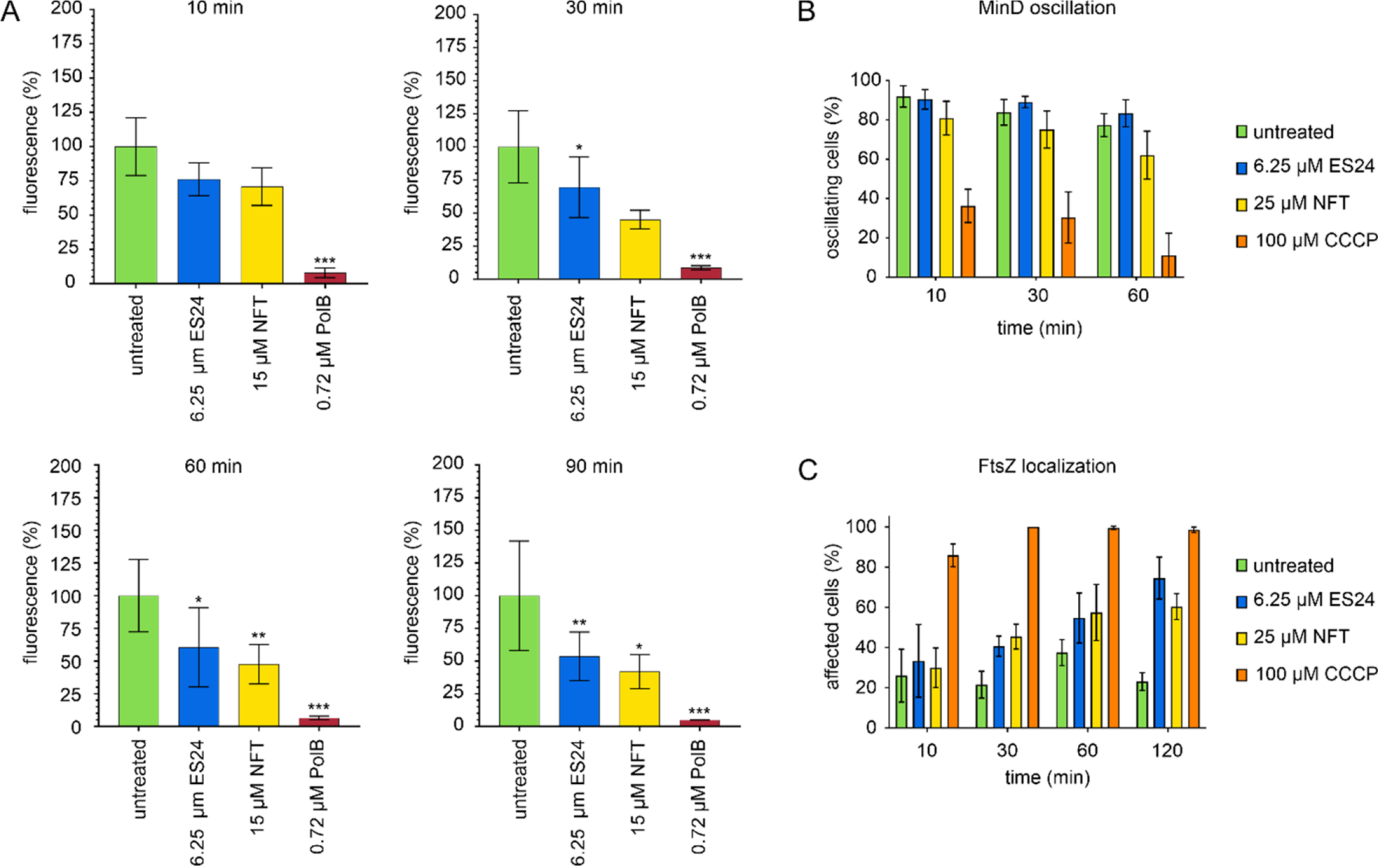
Effects of ES24 on cell membrane function in *E. coli*. (A) DiSC(3)5 measurements of *E. coli* MC4100 carrying pABCON2-*fhuA* ΔC/Δ4L treated with ES24 or NFT. Polymyxin B (PolB) was used as positive control. *p*-Values were calculated using a heteroscedastic, two-tailed *t* test. *p < 0.05, **p < 0.01, ***p < 0.001. (B) MinD oscillation in *E. coli* RC1 carrying pFX9 treated with ES24 or NFT. The proton ionophore CCCP, which is known to abolish MinD oscillation due to membrane depolarization,^40^ was used as positive control. (C) Quantification of FtsZ delocalization. Cells displaying an entirely cytosolic GFP signal, GFP clusters in the cell membrane, double bands, or spirals of FtsZ were counted as delocalized. Cells displaying single FtsZ bands, two opposing foci, or one focus at midcell (indicative of a closing septum) were counted as normal.

Taken together, our data on *B. subtilis* suggest that NFT causes general cell damage through reactive species while ES24 also acts through secretion inhibition. However, the effects of secretion inhibition may have different consequences in Gram-negative bacteria because of the presence of a periplasm. Thus, it is known that secretion stress activates different membrane stress responses in *E. coli*.^43^ Therefore, we used DiSC(3)5 microscopy^44^ to perform membrane potential measurements in *E. coli*. Indeed, we could observe a significant reduction in membrane potential after prolonged treatment with both ES24 and NFT (Figure 8A). A similar, yet less pronounced, trend was observed with paraquat but not hydrogen peroxide, which suggests that the formation of superoxide may also lead to a partial loss of membrane potential (Figure S18).

We next examined whether the observed membrane depolarization has an effect on membrane proteins in *E. coli* by performing timelapse fluorescence microscopy with a strain expressing GFP-labeled MinD.^45^ In *E. coli*, MinD oscillates from pole to pole, and this oscillation is aborted when the membrane potential is dissipated by ionophores, such as carbonyl cyanide *m*-chlorophenyl hydrazone (CCCP).^40^ This property has made fluorescent fusions to MinD a popular reporter for membrane depolarization.^35,44,46^ However, no significant effects on the number of oscillating cells were observed with ES24, thereby suggesting that the partial membrane depolarization observed in the DiSC(3)5 assay is not sufficient to abort MinD oscillation. In contrast, the number of oscillating cells was significantly reduced in NFT-treated cultures and further decreased during prolonged incubation (Figure 8B, Supplementary Figure S19). When examining our time lapse data in more detail, we observed that while MinD still oscillated in ES24-treated samples, this oscillation appeared to slow down in many ES24-treated cells compared with the untreated control. However, the high cell-to-cell variability in oscillation speed did not allow for meaningful analysis and quantification of this observation.

Therefore, we examined the localization of another cell division protein, FtsZ. This tubulin homologue is the major bacterial cell division protein and is anchored to the cell membrane through FtsA.^47^ Like MinD, FtsA is sensitive to dissipation of the membrane potential, yet appears to be more sensitive than MinD.^40,48^ Since FtsA is the only membrane anchor of FtsZ in *E. coli*, FtsA delocalization inevitably causes FtsZ delocalization. In fact, we observed a clear and time-dependent delocalization of FtsZ in both ES24 and NFT-treated *E. coli*. In line with the DiSC(3)5 data, this effect was both time-dependent and stronger for NFT (Figure 8C, Figure S20). We also observed cell elongation, which was previously observed for ES24-treated *E. coli*(16) and can now be explained by FtsZ delocalization.

### Efficacy in a Zebrafish Embryo Infection Model

**Figure 9:**
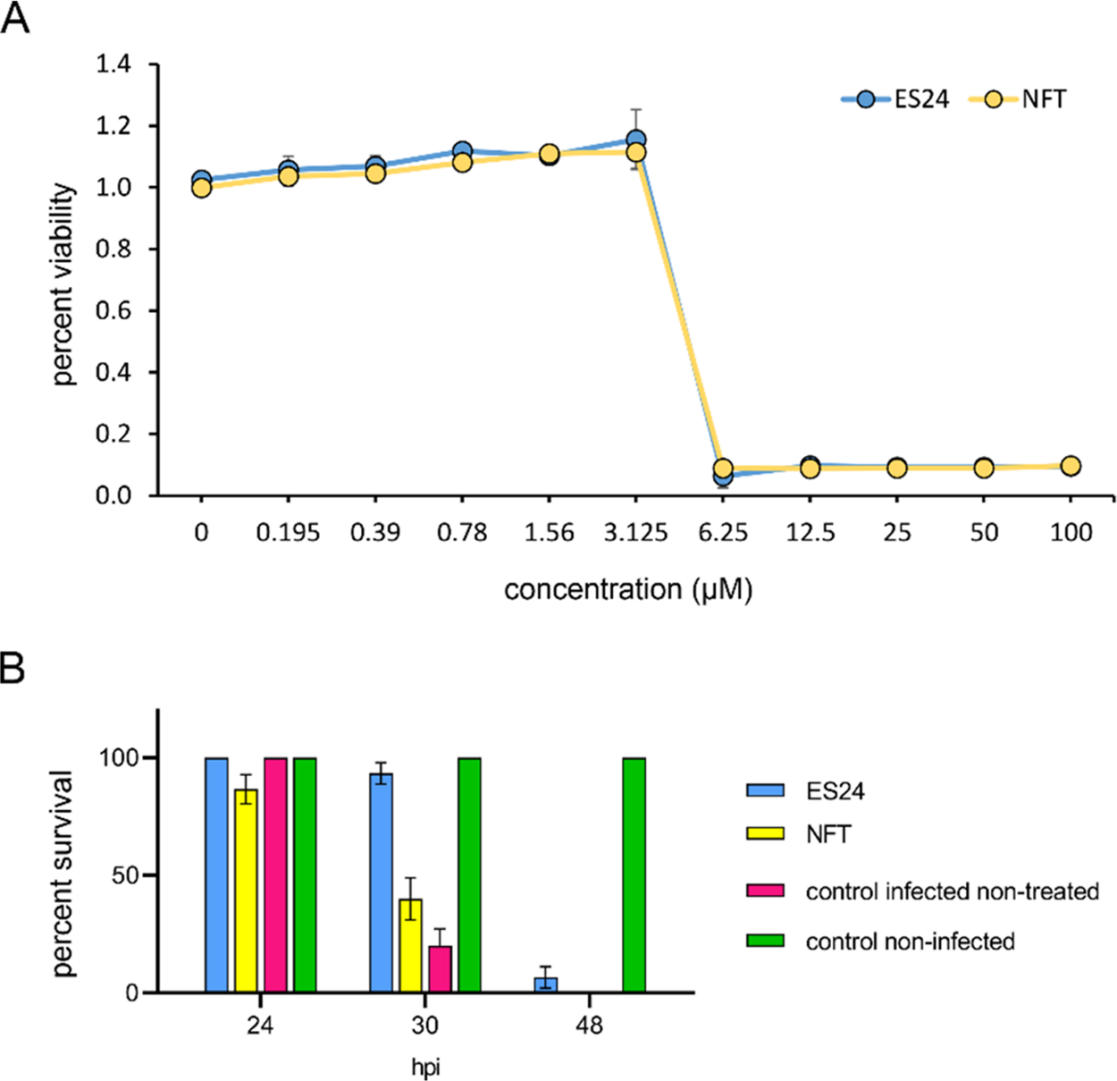
*In vivo* efficacy of ES24 in an *S. pneumoniae* zebrafish embryo infection model. (A) Viability of *S. pneumoniae* D39 after overnight incubation with a concentrations series of ES24 or NFT measured with resazurin. (B) Survival of 2 days postfertilization zebrafish embryos infected with *S. pneumoniae* D39. Embryos were injected with 150 CFU of bacteria in the caudal vein and subsequently treated with 5 μM ES24 or 5 μM NFT by adding the compounds directly to the water 1 h post infection (hpi). ES24 treatment improved the survival rate significantly in zebrafish embryos infected with *S. pneumoniae* D39 compared with NFT-treated embryos (*p* < 0.0001) or nontreated infected embryos (*p* < 0.0001). NFT also improved the survival rate compared with nontreated infected embryos, albeit not significantly (*p* = 0.4139). Data represents the mean ± SEM. Experiments were performed in biological triplicates with 10 fish per condition in each replicate. Survival rates were compared using log-rank statistics.

ES24 shows promising potential as an antibiotic, as demonstrated by its novel mechanism of action, low cytotoxicity, and broad-spectrum antibacterial activity, which is equal to or better than that of NFT against common uropathogens.^11,16^ We further examined its suitability as a future antibiotic drug by assessing its *in vivo* efficacy against *Streptococcus pneumoniae*, a common Gram-positive pathogen, in a zebrafish embryo infection model.^49^ To this end, we first confirmed its bactericidal activity against a *S. pneumoniae* D39 serotype 2 strain *in vitro*. Complete killing was achieved at 6.25 μM, similar to NFT (Figure 9A). After confirming its bactericidal activity, we proceeded with *S. pneumoniae* infection experiments in two-day-old zebrafish embryos by injecting 150 colony-forming units (CFU) of the bacteria into the bloodstream. One hour post infection, the embryos were treated with either ES24 or NFT by adding 5 μM of the respective compound to the water. While ES24 did not eradicate the infection, it significantly increased the survival time and clearly outperformed NFT in these experiments (Figure 9B), thereby underlining its clinical promise.

## Discussion

**Figure 10:**
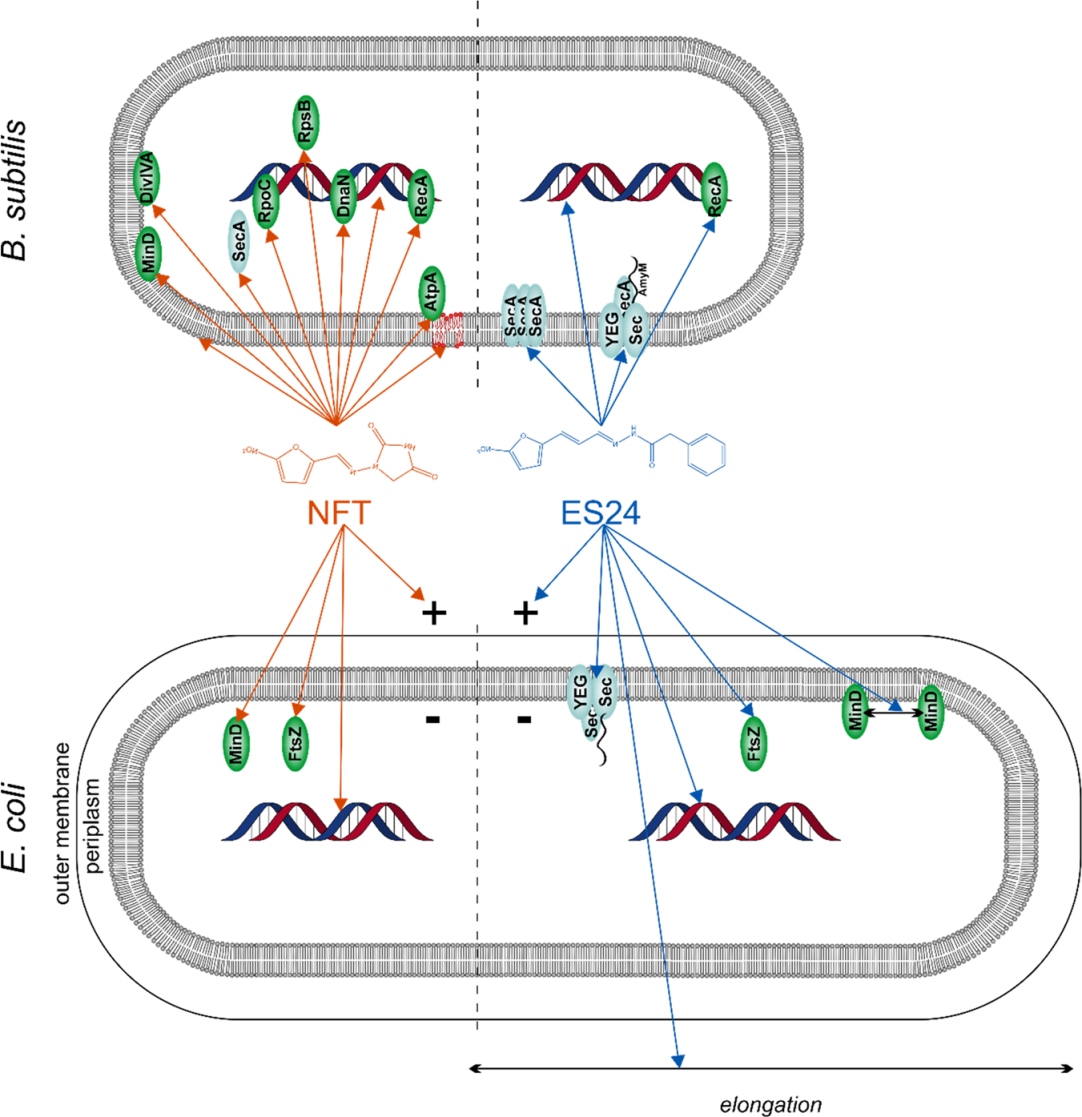
Current model of ES24 mechanism of action in Gram-positive and Gram-negative bacteria in comparison with NFT. NFT affects different cellular processes, including DNA packing; localization of DNA-binding proteins; and membrane integrity, membrane fluidity, and membrane protein binding. No clear differences were observed in its mechanism against *E. coli* and *B. subtilis*. ES24 affects Sec-mediated protein secretion and causes DNA damage in both organisms, but only affects cell division in *E. coli*. Reporter protein functions: MinD and DivIVA, cell division site regulation; FtsZ, cell division; SecA, Sec-dependent protein secretion; AtpA, ATP synthesis; RecA, DNA damage repair; DnaN, replication; RpoC, transcription; RpsB: translation.

ES24 is a promising antibiotic candidate with broad-spectrum activity and a novel mechanism of action. In the present study, we have further evaluated its mechanism of action and compared its effects on Gram-negative and Gram-positive bacteria. Figure 10 summarizes our major findings and takes into account previous results on *E. coli*.^16^ We could show that ES24 indeed inhibits Sec-dependent protein translocation in both Gram-negative and Gram-positive bacteria. Given recent structural evidence that ES1 intercalates in the lateral gate/plug region of the Sec61 secretion system, thus blocking the translocation channel,^14^ it is reasonable to assume that ES24 may bind similarly to the homologous bacterial SecYEG channel. Additionally, ES24 induces DNA damage. Interestingly, in Gram-negative bacteria, ES24 also partially dissipates the membrane potential and leads to cell elongation. We could further show that, despite its structural similarity to NFT and a similar stress response profile, ES24 has a profoundly different mechanism of action, which does not involve generalized macromolecule damage, as observed with NFT. Importantly, ES24 outperformed NFT in a zebrafish embryo pneumococcal infection model.

### Differences in Effects on Gram-Negative and Gram-Positive bacteria

When compared with Gram-negative bacteria, ES24 has stronger antibacterial activity against Gram-positive bacteria,^16^ which may indicate a higher affinity for the Sec-translocon of the latter group of bacteria. Alternatively, the absence of an outer membrane may enhance the ability of ES24 to access its target. Consistent with the second explanation, the MIC value for *E. coli* is reduced when outer membrane permeability is increased by the expression of a mutant β-barrel protein.^16^ Since eukaryotic cells also possess a Sec homologue, cytotoxicity is a potential concern. While ES24 displays a significantly lower cytotoxicity than its parent compound ES1^11^ and did not display acute toxicity in our zebrafish embryo model, a larger therapeutic window may still increase its safety. Thus, while displaying broad-spectrum activity, ES24 may be of higher relevance as future treatment for Gram-positive, versus Gram-negative, infections.

While we could confirm that ES24 does impair Sec-dependent protein translocation in both *E. coli* and *B. subtilis*, we also found marked mechanistic differences between both organisms in our phenotypic analysis. ES24 did not affect the cytoplasmic membrane of *B. subtilis* in terms of membrane potential, fluidity, or morphology, and did not affect the localization of any tested membrane-bound protein other than SecA. In contrast, we did observe a gradual dissipation of the membrane potential and concomitant delocalization of FtsZ in *E. coli*. FtsZ localization depends on the membrane potential since its membrane anchor FtsA is sensitive to depolarization and both proteins lose their membrane binding upon treatment with ionophores.^40^ It was previously observed that *E. coli* cells elongate after prolonged treatment with ES24,^16^ which can now be explained by delocalization of the key cell division protein FtsZ. In *B. subtilis*, both MinD and FtsA are sensitive to dissipation of the membrane potential and so are FtsZ, its secondary membrane anchor SepF, and the cell division site regulation protein DivIVA.^28,40,50^ Thus, membrane depolarization can likewise lead to cell elongation in this organism.^40^ However, since ES24 did not affect the membrane potential in *B. subtilis*, it makes sense that we did not observe any effects on the localization of MinD or DivIVA. In line, we did not observe any change in the cell length of ES24-treated *B. subtilis* cells (Figures 3, 4, 6, and Figures S21 and S22).

The reason why ES24 dissipates the membrane potential in *E. coli* but not in *B. subtilis* is not yet clear. It is possible that the presence of a periplasm makes Gram-negative bacteria more sensitive to secretion defects. Indeed, defects in the Sec translocon induce expression of membrane stress response systems in *E. coli*.^43^ However, this would not explain why the same observation was made for NFT, which did not inhibit protein secretion in either organism. It could be speculated that both compounds, being structurally similar, have a higher affinity for Gram-negative than Gram-positive cell membranes, which could, for example, lead to stronger membrane disturbances upon passing through the lipid bilayer, possibly caused by an extended translocation time, as previously suspected for membrane-active atypical tetracyclines.^21,51^

In contrast to our phenotypic assays, no apparent membrane stress response was found in the *E. coli* transcriptome analysis,^16^ while in *B. subtilis* at least some membrane stress responses were activated (Figure 1). However, the most typical markers for membrane stress in *B. subtilis*, which are induced upon membrane depolarization, changes in membrane fluidity, or membrane protein delocalization, were not found to be upregulated in the transcriptome.^35,36,52^ The absence of these markers is consistent with the absence of any of these effects in ES24-treated cells. While we cannot fully explain the absence of membrane stress markers in *E. coli*, despite a clear effect on both membrane potential and membrane protein localization, the transcriptome analysis was performed after 15 min of treatment while first phenotypic effects set in after 30 min. Thus, it is possible that transcriptome profiling would show a corresponding membrane stress response when analyzed after longer antibiotic exposure.

### Differences between ES24 and NFT

Both ES24 and NFT carry a nitrofuran ring (Figure 1A), and both compounds require activation by bacterial nitroreductases.^16,23,24^ Therefore, a first notion was that they may have a similar mechanism of action. Transcriptomic profiling of both *E. coli* and *B. subtilis* did show a significant overlap between the stress responses to ES24 and NFT (Figure 1B,C).^16^ However, it was previously shown that only ES24, but not NFT, inhibited protein secretion in *E. coli*,^16^ and we have now shown the same for *B. subtilis* (Figure 7). Moreover, our phenotypic analysis consistently showed major differences between ES24 and NFT in almost every assay performed, which suggests a rather specific mechanism of ES24 on the Sec translocon and more general macromolecule and structural damage by NFT.

Both ES24 and NFT elicited a DNA damage response in both *E. coli*(16) and *B. subtilis* (Figure 1B,C). Using a *B. subtilis* RecA-GFP strain, we could now show that, indeed, both antibiotics induce DNA damage (Figure 4). This was further corroborated by DAPI staining, which revealed morphological changes to the nucleoid after prolonged treatment with 4 μM ES24 (Figure 3). In contrast, NFT led to full dispersion of the DAPI signal, most likely because of complete structural disintegration of the nucleoid, as previously suggested by both fluorescence and electron microscopy imaging.^21^ Moreover, NFT caused large-scale effects on the localization of the DNA-associated proteins DnaN and RpoC, as well as RpsB, which is excluded from the nucleoid, while ES24 did not affect any of these reporters (Figures S6–S8). Taken together, both compounds induce DNA damage, yet NFT appears to be much more aggressive and structurally disintegrates the nucleoid.

A similar pattern was observed for the cell membrane in *B. subtilis*. While NFT caused membrane damage, which was apparent in the Nile red stain and laurdan-based fluidity measurements, and delocalized all tested membrane proteins, ES24 had no effect in any membrane assay with the exception of SecA localization. While this was clearly different in *E. coli*, where ES24 did affect the cell membrane, NFT displayed stronger effects on the membrane potential, MinD oscillation, and FtsZ localization than ES24 (Figure 8, Figures S18–S20), thereby confirming the notion that the mechanism of NFT is broader and more aggressive than that of ES24.

NFT has previously been proposed to cause oxidative damage to cellular macromolecules, in particular DNA and cell membrane lipids.^21−24^ However, it has not yet been clear whether it leads to the formation of ROS, such as superoxide and hydroxyl radicals, or whether activation by cellular nitroreductases generates reactive products that then oxidize cellular components. Here, we found no evidence for ROS playing a role for NFT activity, thereby supporting the latter explanation (Figure 2).

This raises the question as to whether the same, or similar, derivative species are formed upon nitroreductase-dependent activation of ES24 and NFT. For instance, it is conceivable that these compounds both lead to the formation of nitric oxide or other reactive nitrogen species, but to different extents.^53^ This could explain the observed SOS response and RecA activation, as well as the overall similar, general stress response to ES24 and NFT. It would also fit with our observation that the DNA damage and nucleoid morphology changes induced by ES24 appear to be concentration-dependent. Since such a mechanism would depend on the number of reactive species released, and the activity of ES24 and NFT differs by a factor of 10, this explanation would also fit well with the generally more pronounced effects observed with NFT. It is conceivable that equimolar concentrations of ES24 would cause similar effects as NFT; however, this hypothesis could not be tested since 8 μM of ES24 already completely killed *B. subtilis* cultures (Figure S1).

### Conclusion

Taken together, we propose that ES24 primarily acts by inhibiting the Sec translocation machinery together with damaging DNA through the generation of reactive species. We propose that in Gram-negative bacteria, membrane damage caused by aberrant protein insertion additionally contributes to its activity. Strikingly, ES24 outcompeted NFT in our zebrafish embryo infection model using the human pathogen *S. pneumoniae*, which suggests that its higher *in vitro* activity corresponds to better *in vivo* efficacy. These results highlight ES24 as a promising new lead structure for further drug development.

## Methods

### Antimicrobial Compounds

ES24 was synthesized according to Gamayun et al.^11^ Nitrofurantoin and benzyl alcohol were purchased from Acros Organics; gramicidin and CCCP were purchased from Alfa Aesar; chloramphenicol and hydrogen peroxide were purchased from Fisher Bioreagents; polymyxin B was purchased from Duchefa; and paraquat was purchased from Sigma-Aldrich. Stock solutions were prepared in sterile water (chloramphenicol, hydrogen peroxide) or sterile DMSO (all other compounds). Aliquots were kept at 4 °C (hydrogen peroxide) or −20 °C (all other compounds) until further use. Benzyl alcohol dilutions were freshly prepared for each day of experiments. Unless stated otherwise, the following antibiotic concentrations were used throughout the study: 2 μM and 4 μM ES24, 25 μM nitrofurantoin, 0.53 μM gramicidin, 100 mM benzyl alcohol, 0.3% hydrogen peroxide, and 500 μM paraquat for *B. subtilis* and 6.25 μM ES24, 0.72 μM polymyxin B, and 100 μM CCCP for *E. coli*.

### Strains and Growth Conditions

Strains and plasmids used in this study are listed in Table S4. All *B. subtilis* and *E. coli* strains were aerobically grown in lysogeny broth (LB) supplemented with appropriate inducer concentrations where indicated (see Table S4). Unless stated otherwise, *E. coli* and *B. subtilis* cultures were grown at 30 °C. *S. pneumoniae* was grown in competence medium plus yeast (C+Y medium) at 37 °C.^54^ Unless stated otherwise, experiments were performed in biological triplicates, and error bars represent the standard deviations of independent replicate experiments.

### Strain Construction

Primers used for strain construction are listed in Table S5. Strain *B. subtilis* BSN101 (*secA::Pxyl-secA-gfp cat*) was constructed by restriction cloning using the pSG1164 plasmid backbone.^55^ The *secA* gene was amplified from *B. subtilis* 168CA chromosomal DNA using the primer pair SV77/SV78 and cloned into pSG1164 using Asp718 and *Sal*I restriction sites. The resulting plasmid pSH54 was transformed into *B. subtilis* 168CA using a standard starvation protocol.^56^ Campbell integration into the native locus *secA* was confirmed by PCR, resistance pattern, and fluorescence microscopy.

Strain BWB09 was constructed by marker-free deletion of the *xynA* and *amyE* genes in the tryptophan–prototrophic background strain BSB1^57^ following the method by Morimoto et al.^58^ Purified amplicons of the *xynA* upstream (primer pair BW45/BW46) and downstream sequences (BW41/42), the Sp^R^-*mazF* cassette (BW05/BW06), and the *xynA* gene (BW47/BW48) were fused by overlap extension PCR (BW45/BW48) to create the *xynA* deletion cassette. The resulting recombinant DNA fragment was used to transform competent *B. subtilis* BSB1, followed by selection for spectinomycin resistance (150 μg/mL). Subsequently, expression of the *mazF* toxin was induced by the addition of 1 mM IPTG, which resulted in excision of the deletion cassette and, thus, marker-free deletion of *xynA* (strain BWB06). In a second analogous step, the *amyE* gene was deleted. Purified amplicons of the *amyE* upstream (BW49/BW51) and downstream sequences (BW50/BW52), the Sp^R^-*mazF* cassette (BW05/BW06), and the *amyE* gene (BW53/BW54) were fused by overlap extension PCR (BW49/BW54), and the resulting deletion cassette was used to transform competent BWB06. Selection for spectinomycin resistance followed by IPTG-forced excision of the deletion cassette resulted in a marker-free double deletion of *xynA* and *amyE* (strain BWB09). The strains were confirmed by sequencing.

### Minimal Inhibitory Concentration and Growth Experiments

Minimal inhibitory concentrations (MICs) were determined in a serial microdilution assay according to guidelines issued by the Clinical Laboratory Standardization Institute (CLSI), as described previously.^27^ In short, serial 2-fold dilutions of the compound of interest were prepared in sterile 96-well plates and inoculated with 5 × 10^5^ CFU of *B. subtilis* 168CA per mL. MIC plates were incubated at 37 °C under steady agitation for 16 h. Optical density readings were taken at 600 nm. Growth experiments aimed at finding an appropriate stress level for physiological experiments were performed with *B. subtilis* 168CA, as described previously.^26^ MICs and growth experiments were performed in biological duplicates.

### Transcriptomics

*B. subtilis* 168CA cells were grown at 30 °C to mid-log phase, diluted to an OD_600_ of 0.05, and subsequently treated with 0.5% DMSO (untreated control), 3 μM ES24, or 25 μM nitrofurantoin for 15 min. Cultures were then pelleted by centrifugation (5000 × *g*, 5 min), and total RNA was extracted using the Qiagen RNA purification kit according to the manufacturer’s instructions. Transcriptomics and differential expression analysis were performed by Macrogen (Seoul, Korea). The experiments were performed in duplicates. Transcripts that showed a log^2^-fold change of ≥3 (*p* < 0.05) were considered differentially regulated.

### Fluorescence Light Microscopy

Unless stated otherwise, all microscopy experiments were performed on a Nikon Eclipse Ti2 equipped with a CFI Plan Apochromat DM Lambda 100× Oil objective (N.A. 1.45, W.D. 0.13 mm), a Photometrics PRIME BSI camera, a Lumencor Sola SE II FISH 365 light source, and an Okolab temperature incubation chamber. Images were obtained using the NIS-Elements AR software version 5.21.03 and analyzed with ImageJ.^59^ Quantification of the microscopy images was performed using the ImageJ plugins ObjectJ^60^ and MicrobeJ.^61^

### Oxyburst Green Assay

Oxyburst Green H2DCFDA succinimidyl ester was purchased from Thermo Fisher and dissolved in sterile DMSO. Aliquots were covered with nitrogen gas and stored at −20 °C until further use. *B. subtilis* 168CA was inoculated from overnight cultures in fresh LB medium to an OD_600_ of 0.05 and allowed to grow until early log phase (OD_600_ = 0.4) prior to the addition of antibiotics. Samples were taken after 10, 30, 60, and 120 min of antibiotic incubation, respectively. Staining with 10 μM Oxyburst Green was performed for 160 min, with the respective antibiotic incubation times included. After staining and antibiotic treatment, cells were washed once with phosphate-buffered saline (10 mM phosphate buffer, 2.7 mM KCl, 137 mM NaCl, pH 7.4), immobilized on glass slides covered with a thin film of 1.2% agarose,^44^ and imaged immediately. Images were analyzed with MicrobeJ.^61^ The parameters for bacterial recognition were set to an area of 1.5 max, length of 1 max, width of 0.5–2.5, curvature of 0–1.5, and an angularity of 0–0.5 for detection in phase contrast. All other parameters remained at default settings. For detecting the fluorescence intensity, parameters were set to an area of 0.7–4.5, length of 1.2–4, and width of 2.5.

### ROS Scavenger Assay

Stock solutions of tiron (Acros Organics) and thiourea (Alfa Aesar) were prepared in sterile water at 1 and 2.5 M, respectively. Serial 2-fold dilutions of antimicrobial compounds were prepared in LB containing either 10 mM tiron, 150 mM thiourea, both 10 mM tiron and 150 mM thiourea, or no scavenger. *B. subtilis* 168CA was added to a final CFU count of 5 × 10^5^ CFU/mL. Cells were incubated for 16 h at 30 °C. Optical density was measured by absorbance readings at 600 nm using a BMG Clariostar Plus plate reader.

### Bacterial Cytological Profiling

Bacterial cytological profiling was performed according to Wenzel et al.^26^ In short, *B. subtilis* 168CA was grown until an OD_600_ of 0.3 prior to antibiotic addition, and samples were taken after 10, 30, 60, and 120 min of antibiotic treatment. Samples were then stained with 0.5 μM Nile red or MitoTracker Green (membrane stains, Invitrogen) and 1 μg/mL of DAPI (DNA stain, Invitrogen) for 1 min, spotted on 1.2% agarose films, and imaged immediately. Images were processed with ImageJ and analyzed with ObjectJ.^60^

### DiSC(3)5 Spectroscopy

DiSC(3)5 is a dye that accumulates in polarized cell membranes. Because of its self-quenching properties, a release of the dye from the cell membrane following depolarization results in an increased fluorescence in the cell suspension, which can be observed spectroscopically.^44^ Stock solutions of DiSC(3)5 (Anaspec) were prepared at 100 μM in sterile DMSO and stored at −20 °C until further use. The membrane potential in *B. subtilis* 168CA was determined by DiSC(3)5 spectroscopy. To this end, cultures were grown to an OD_600_ of 0.3 in the presence of 50 μg/mL of BSA. Kinetic measurements were performed on a Biotek Synergy MX plate reader, as described previously.^26^ Kinetic measurements longer than 30 min become imprecise because of photobleaching. Therefore, end point measurements were performed to assess membrane potential after prolonged treatment times. To this end, samples were taken after 10, 30, 60, and 90 min of antibiotic treatment and subsequently stained with DiSC(3)5. Fluorescence was measured at an excitation wavelength of 610–30 nm and an emission wavelength of 675–50 nm in a BMG Clariostar Plus plate reader. Three technical replicates were performed for each biological replicate.

### DiSC(3)5 Microscopy

Membrane potential measurements of *E. coli* MC4100 carrying plasmid pABCON2-*fhuA* ΔC/Δ4L^62^ were performed by DiSC(3)5 microscopy according to te Winkel et al.^44^ In contrast with spectroscopic measurements, where an increase in the total signal of the sample is observed upon depolarization because of dequenching of the dye, microscopic measurements show a decrease in single-cell fluorescence upon release of the dye. This is due to the remaining signal of the quenched fluorescence still being strong enough to be detected microscopically with a good signal to background ratio. Detachment of the dye from the cell membrane results in a clearly reduced fluorescence signal in single cells.^44^ The expression of FhuA increases outer membrane permeability,^62^ results in better uptake of DiSC(3)5, and subsequently, better sensitivity and signal stability in this assay. Cells were grown to an OD_600_ of 0.3 prior to antibiotic treatment. DiSC(3)5 staining was carried out for 10 min immediately prior to microscopy. Samples were taken after 10, 30, 60, and 90 min of antibiotic stress; spotted on glass slides covered with 1.2% agarose; and imaged immediately. Images were analyzed with MicrobeJ.^49^ The parameters for bacterial recognition were set to an area of 0.7–4.65, length of 1.7–4.9, width of 0.6–2.5, circularity of 0.45–1, curvature of 0–1.5, and an angularity of 0–1 for detection in phase contrast. All other parameters remained at default settings. For detecting the fluorescence intensity, parameters were set to an area of 0.7–4.5, length of 1.2–4, and width of 2.5.

### MinD Oscillation Microscopy

MinD oscillation in *E. coli* PFX9 expressing GFP-MinD^63^ was observed by timelapse microscopy. Cells were induced at an OD_600_ of 0.1 by the addition of 10 μM IPTG and allowed to grow until an OD_600_ of 0.2 prior to the addition of antibiotics. Samples were taken after 10, 30, and 60 min of antibiotic treatment; immobilized on glass slides covered with a 1.2% agarose film; and immediately imaged. Images were taken for 4 min at 15 s intervals. The counting of oscillating and nonoscillating cells was done manually.

### Laurdan Spectroscopy

Stock solutions of laurdan (Anaspec) were prepared at 10 mM in sterile dimethylformamide and stored at −20 °C until further use. Kinetic membrane fluidity measurements were performed as described previously^39^ with minor modifications. In short, cells were grown in the presence of 0.2% glucose until an OD_600_ of 0.5 and subsequently stained with 10 μM laurdan for 5 min. The cells were washed 5 times with laurdan buffer (PBS, 0.2% glucose, 1% DMF) and resuspended in the same buffer to a final OD_600_ of 0.8. Samples of 100 μL were withdrawn and added to a prewarmed black 96-well polystyrene microplate (Corning). Laurdan fluorescence was measured at an excitation of 350–15 nm and emission of 460–15 nm and 500–15 nm in a BMG Clariostar Plus plate reader. After recording the untreated baseline for 10 min, 100 μL of prewarmed laurdan buffer containing the respective antibiotics was added, and measurements were continued for 30 min. Longer kinetic measurements become imprecise because of photobleaching. Therefore, additional end point measurements were performed to assess membrane fluidity after prolonged treatment times. End point measurements were obtained by following the same workflow until the resuspension step. Cell suspensions were adjusted to an OD_600_ of 0.4 prior to antibiotic addition and further incubated at constant temperature and agitation. Samples of 200 μL were taken after 10, 30, 60, and 120 min; transferred to microplates; and measured as described above. Laurdan general polarization values were calculated according to ref (39).

### Protein Localization

Protein localization studies were performed as previously described.^26^ In short, *B. subtilis* and *E. coli* strains expressing the different GFP fusion proteins were grown in the presence of appropriate inducer concentrations (Table S4) until an OD_600_ of 0.3 and subsequently stressed with antibiotics. Samples were taken after 10, 30, and 120 min of treatment; spotted on glass slides coated with a thin film of 1.2% agarose; and immediately imaged using a Nikon Eclipse Ti microscope equipped with a CFI Plan Apochromat DM 100× oil objective, an Intensilight HG 130 W lamp, a C11440-22CU Hamamatsu ORCA camera, and an Okolab temperature incubation chamber. Images were recorded with NIS elements software, version 4.20.01, and processed with ImageJ.

### AmyM Secretion

*B. subtilis* BWB9 carrying pCS73 (*PamyQ-amyM*) was grown in LB medium at 37 °C until mid-log phase, followed by washing twice in fresh LB medium and resuspension to a final OD_600_ of 0.05. Subsequently, the cells were treated with ES24 (2 μM, 1 μM, 0.5 μM), NFT (12 μM, 6 μM, 3 μM), or DMSO (0.5%) as a negative control, and growth was continued for 3 h. The cells were collected by centrifugation at 5000 × *g* for 5 min, and the culture supernatant was subjected to trichloroacetic acid precipitation at 4 °C overnight. Both the cell lysate and supernatant were analyzed by 9% SDS-PAGE, followed by Coomassie staining and Western blotting. AmyM was detected using a rabbit α-AmyM primary antibody^64^ in 1:20 000 dilution and a horseradish peroxidase-conjugated goat α-rabbit secondary antibody (Rockland) in 1:10 000 dilution. PycA was detected with a streptavidin horseradish peroxidase conjugate (Sigma-Aldrich) in 1:2000 dilution. Sample loading on the protein gel was adjusted on the basis of the OD_600_ of the culture prior to collection for analysis.

### LipA Secretion

*B. subtilis* TEB1030 (*ΔlipA*) carrying pBSlipA^42^ to express the *lipA* gene from the constitutive *PhpaII* promoter was grown in LB broth. After reaching midexponential growth phase, the cells were collected and washed twice with LB growth medium and resuspended in LB to a final OD_600_ of 0.05. Subsequently, different concentrations of ES24 or NFT were added, and cultures were incubated for 3 h under vigorous shaking (in the dark) until midexponential growth phase. Samples were collected, and the cells and growth medium fractions were separated by centrifugation. Bacterial cells were disrupted by bead-beating and, subsequently, proteins in the cell and growth medium fraction were separated by LDS-PAGE using 10% NuPage gels (Life Technologies). Gel loading of the samples was corrected on the basis of cellular protein content as determined with the Pierce bicinchoninic acid (BCA) Protein Assay Kit (Thermofisher Scientific, USA). Gels were stained with InstantBlue Coomassie Protein Stain (AbCam). The experiment was performed in duplicate.

### Resazurin Assay

The cell viability of *S. pneumoniae* D39 was measured in a standard resazurin bacterial viability assay in 96-well plates, as previously reported.^65^ In brief, 20 μL of resazurin solution [0.025% (w/v) resazurin sodium] were added to each well containing bacterial cultures. After color conversion of the dye, bacterial viability was measured on the basis of fluorescence intensity using a BioTek plate reader (Synergy H1) with bottom reading mode (excitation/emission, 560 nm/590 nm).

### Zebrafish Embryo Experiments

All methods were carried out in accordance with relevant guidelines and regulations. *Danio rerio* (zebrafish) were handled in compliance with the local animal welfare regulations and maintained according to standard protocols (zfin.org). The breeding of zebrafish in authorized institutions such as the Amsterdam Animal Research Center of the Vrije Universiteit (VU) Amsterdam is in full compliance with the Dutch law on animal research. All animal experiments are supervised by the local Animal Welfare Body (Instantie voor Dierenwelzijn, IvD) of the VU and the VU Medical Center (IvD VU/VUmc). All used research protocols adhere to the international guidelines on the protection of animals used for scientific purposes, that is, the EU Animal Protection Directive 2010/63/EU, which allows zebrafish embryos to be used up to the moment that they are able to independently take up external food (5 days after fertilization) without additional approval by the Central Committee for Animal Experiments in The Netherlands (Centrale Commissie Dierproeven, CCD). Because the zebrafish embryos used in this study meet these criteria, this specific study was therefore approved by the IvD VU/VUmc. *Casper* zebrafish embryos were infected at 2 days postfertilization in the caudal vein by microinjection with 150 CFU of *S. pneumoniae* D39, as previously described.^49^ The embryos were treated 1 h postinjection (hpi) by addition of 5 μM of either ES24 or NFT to the water. Infected embryos were monitored for survival at 24, 30, and 48 hpi. All experiments were performed in triplicate.

## Supporting Information Available

Growth experiments, additional ROS assay controls, quantification and overview images of microscopy, peroxide and paraquat controls, protein localization assays, additional protein secretion experiments and controls, microscopy images of membrane potential and cell division assays in *E. coli*, cell length measurements, transcriptomics data tables, list of strains, plasmids, and primers, supplementary references (PDF)
Transcriptomics raw data (XLSX)
