# Supplementary material for: Dual Action of Eeyarestatin 24 on Sec-Dependent Protein Secretion and Bacterial DNA
Source: ACS Infect Dis. 2023 Jan 13;9(2):253–69. doi: 10.1021/acsinfecdis.2c00404 (PMC9926488; doi:10.1021/acsinfecdis.2c00404)
Supplement: Supplementary file 1 — id2c00404_si_001.pdf [file id2c00404_si_001.pdf]

Supplementary Information

Dual action of eeyarestatin 24 on Sec-dependent protein secretion and bacterial DNA

Ann-Britt Schäfer[1#], Maurice Steenhuis[2#¤], Kin Ki Jim[3,4], Jolanda Neef[5], Sarah O'Keefe[6§], Roger C. Whitehead[7], Eileithyia Swanton[6], Biwen Wang[8], Sven Halbedel[9,10], Stephen High[6], Jan Maarten van Dijl[5], Joen Luirink[2]*, Michaela Wenzel[1]*

[1]Division of Chemical Biology, Department of Biology and Biological Engineering, Chalmers University of Technology, 412 96 Gothenburg, Sweden
[2]Molecular Microbiology, Amsterdam Institute of Molecular and Life Sciences (AIMMS), Vrije Universiteit Amsterdam, 1081 HV Amsterdam, The Netherlands
[3]Department of Medical Microbiology and Infection Prevention, Amsterdam University Medical Centers - Location Vrije Universiteit Amsterdam, 1081 HZ Amsterdam, The Netherlands
[4]Amsterdam Institute for Infection and Immunity, Amsterdam University Medical Centers, 1081 HZ Amsterdam, The Netherlands
[5]Department of Medical Microbiology, University of Groningen, University Medical Center Groningen, Hanzeplein 1, PO Box 30001, 9700 RB Groningen, The Netherlands
[6]School of Biological Sciences, Faculty of Biology, Medicine and Health, University of Manchester, Manchester, M13 9PL, United Kingdom
[7]School of Chemistry, Faculty of Science and Engineering, University of Manchester, Manchester, M13 9PL, United Kingdom
[8]Bacterial Cell Biology and Physiology, Swammerdam Institute for Life Sciences, University of Amsterdam, 1098 XH Amsterdam, The Netherlands
[9]FG11 Division of Enteropathogenic Bacteria and Legionella, Robert Koch Institute, 38855 Wernigerode, Germany
[10]Institute for Medical Microbiology and Hospital Hygiene, Otto von Guericke University Magdeburg, 39120 Magdeburg, Germany

*Authors to whom correspondence should be addressed:

Joen Luirink: s.luirink@vu.nl; Michaela Wenzel: wenzelm@chalmers.se

Figure S1: Growth inhibition of *B. subtilis* 168CA by ES24 and NFT.

Figure S2: Effect of different ROS sources on Oxyburst Green fluorescence.

Figure S3: Quantification of bacterial cytological profiling images shown in Figure 3.

Figure S4: Bacterial cytological profiling of *B. subtilis* 168CA treated with hydrogen peroxide and paraquat.

Figure S5: Quantification of RecA microscopy images.

Figure S6: Localization of DnaN-GFP after treatment with ES24 and NFT.

Figure S7: Localization of RpoC-GFP after treatment with ES24 and NFT.

Figure S8: Localization of RpsB-GFP after treatment with ES24 and NFT.

Figure S9: Membrane fluidity of *B. subtilis* 168CA after treatment with peroxide and paraquat.

Figure S10: Localization of GFP-MinD after treatment with ES24 and NFT.

Figure S11: Localization of DivIVA-GFP after treatment with ES24 and NFT.

Figure S12: Localization of AtpA-GFP after treatment with ES24 and NFT.

Figure S13: Overview images of *B. subtilis* BSN101 expressing SecA-GFP after 4 h of antibiotic treatment.

Figure S14: Localization of SecA-GFP at different timepoints.

Figure S15: AmyM secretion assay loading controls.

Figure S16: Western blot of cell pellets.

Figure S17: Effects on LipA secretion.

Figure S18 DiSC(3)5 measurements of *E. coli* MC4100 carrying pABCON2-*fhuA* ΔC/Δ4L treated with peroxide, paraquat, and CCCP.

Figure S19: MinD oscillation in *E. coli* RC1 carrying pFX9 treated with ES24, and NFT.

Figure S20: Effects on FtsZ localization in *E. coli*.

Figure S21: Cell length measurements of *B. subtilis* 168CA treated with ES24 and NFT.

Figure S22: Cell length measurements of *B. subtilis* 168CA treated with peroxide and paraquat.

Table S1: Transcripts induced by both ES24 and NFT.

Table S2: Transcripts uniquely induced by ES24.

Table S3: Transcripts uniquely induced by NFT.

Table S4: Strains and plasmids used in this study.

Table S5: Primers used in this study.

Supplementary References

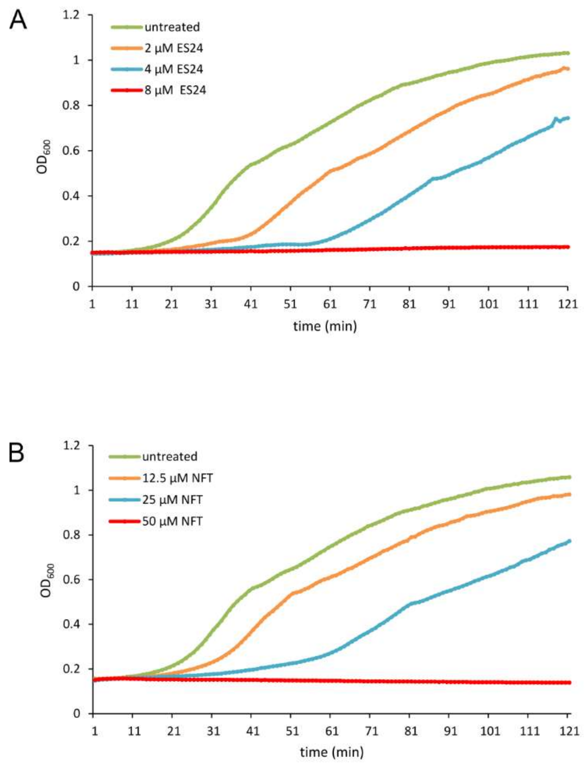

Figure S1: Growth inhibition of *B. subtilis* 168CA by ES24 **(A)** and NFT **(B)**.

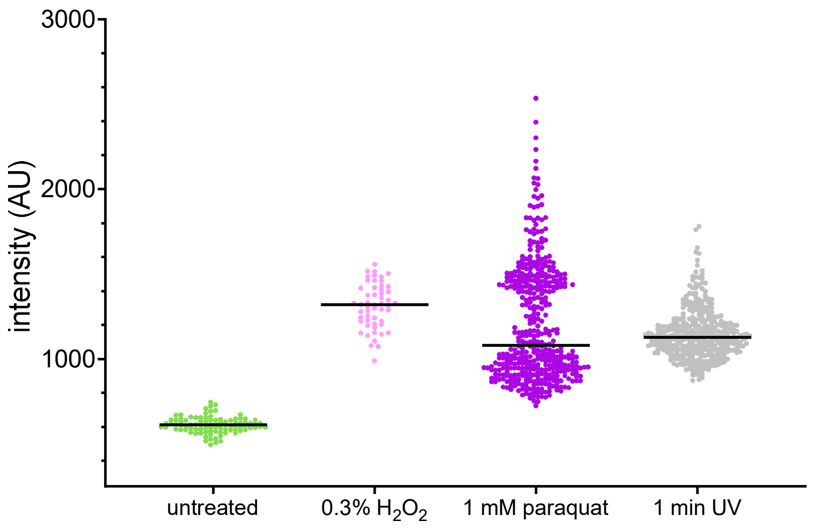

Figure S2: Effect of different ROS sources on Oxyburst Green fluorescence. *B. subtilis* 168CA was treated with peroxide or paraquat for 10 min, or exposed to UV light for 1 min, prior to ROS detection with the Oxyburst Green probe. Whole cell fluorescence was quantified for each single cell using MicrobeJ.

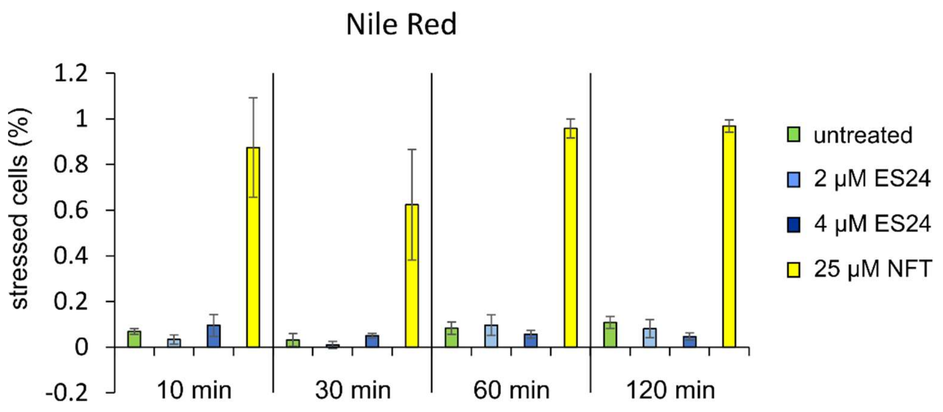

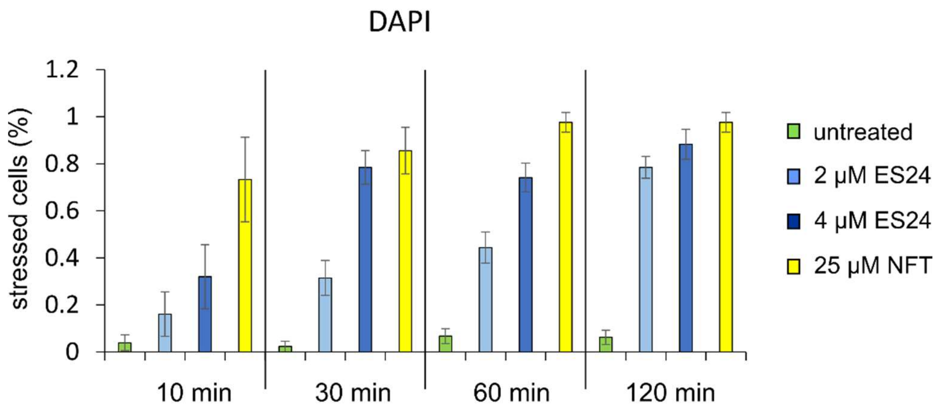

Figure S3: Quantification of bacterial cytological profiling images shown in Figure 3. *B. subtilis* 168CA was treated with ES24 or NFT and cells were counted according to their phenotype. For the Nile red stain, cells with membrane foci of any kind were counted as stressed while cells with smooth membrane stains were defined as not stressed. For DAPI, both nucleoid compaction and relaxation as well as disappearance of the DAPI signal were counted as stressed, while cells with regularly packed and spaced nucleoids were counted as unstressed. A minimum of 50 cells were counted per condition. Error bars show standard deviation of three replicates.

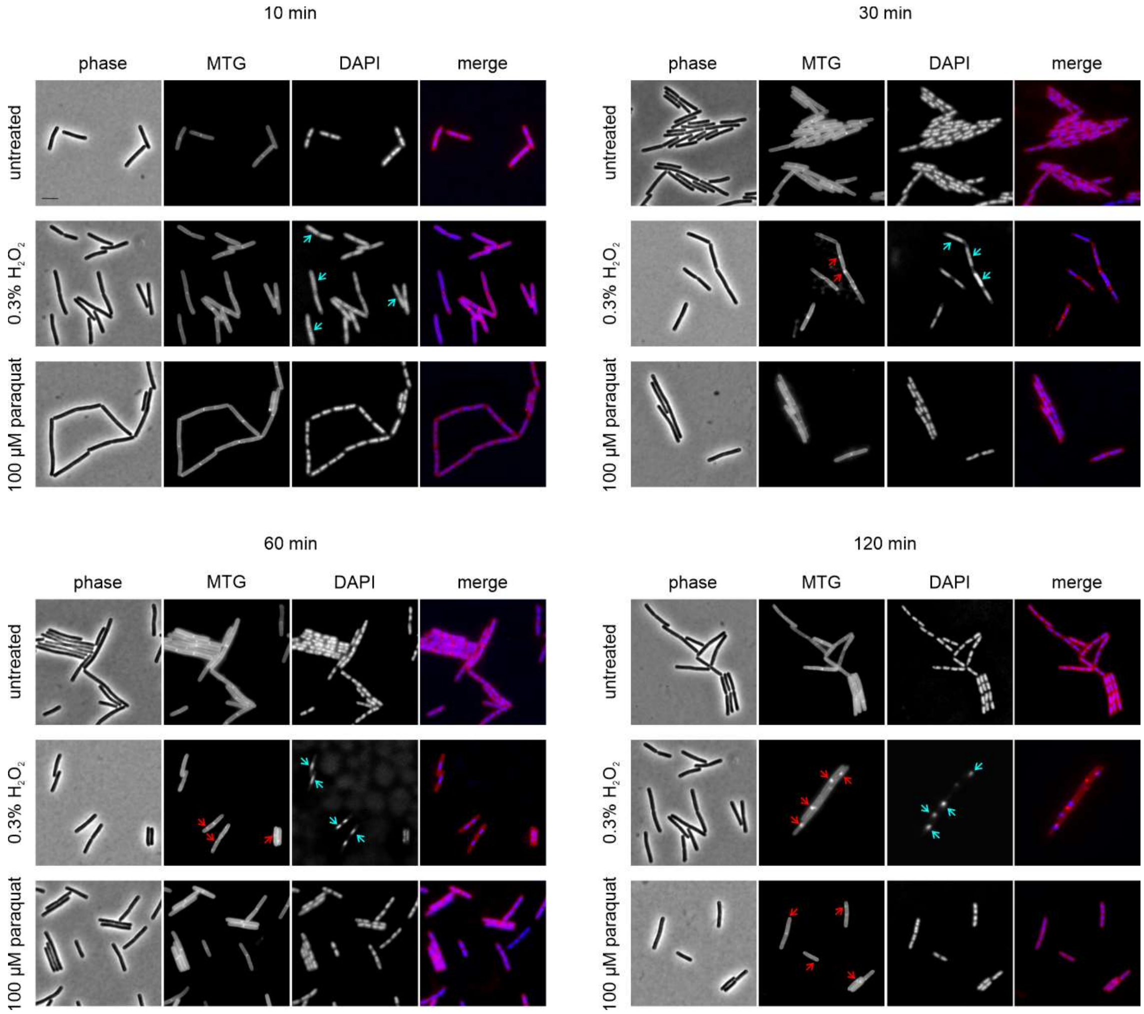

Figure S4: Bacterial cytological profiling of *B. subtilis* 168CA treated with hydrogen peroxide or paraquat. Merged images show the membrane stain in red and the DAPI stain in blue. Red arrows indicate membrane aberrations. Blue arrows indicate DNA packing defects. Scale bar 2 µm.

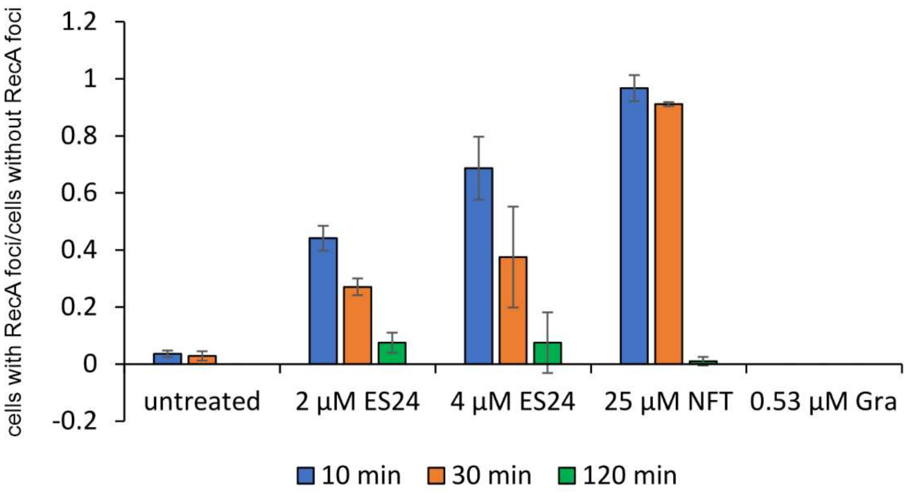

Figure S5: Quantification of RecA microscopy images. *B. subtilis* UG10 was treated with ES24, NFT, or gramicidin (Gra) and cells with distinct RecA foci were counted and expressed relative to the number of total cells. A minimum of 50 cells were counted per condition. Error bars show standard deviation of three replicates.

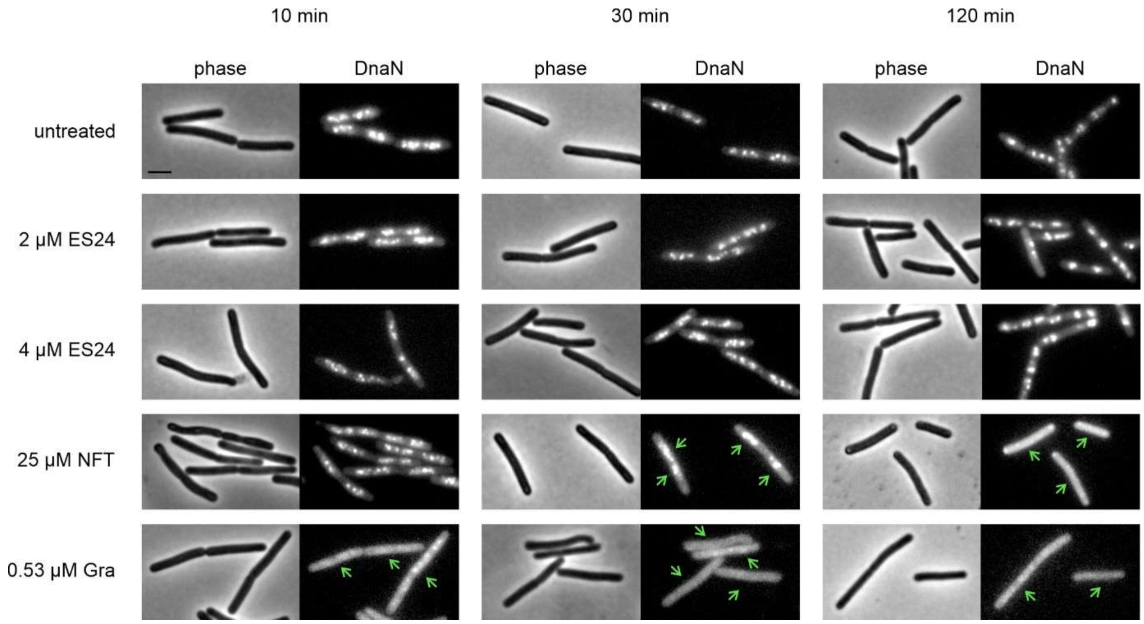

Figure S6: Localization of DnaN-GFP (strain *B. subtilis* HM771) after treatment with ES24 or NFT. Gramicidin (Gra) was used as additional control. Green arrows indicate dispersed protein. Scale bar 2 µm.

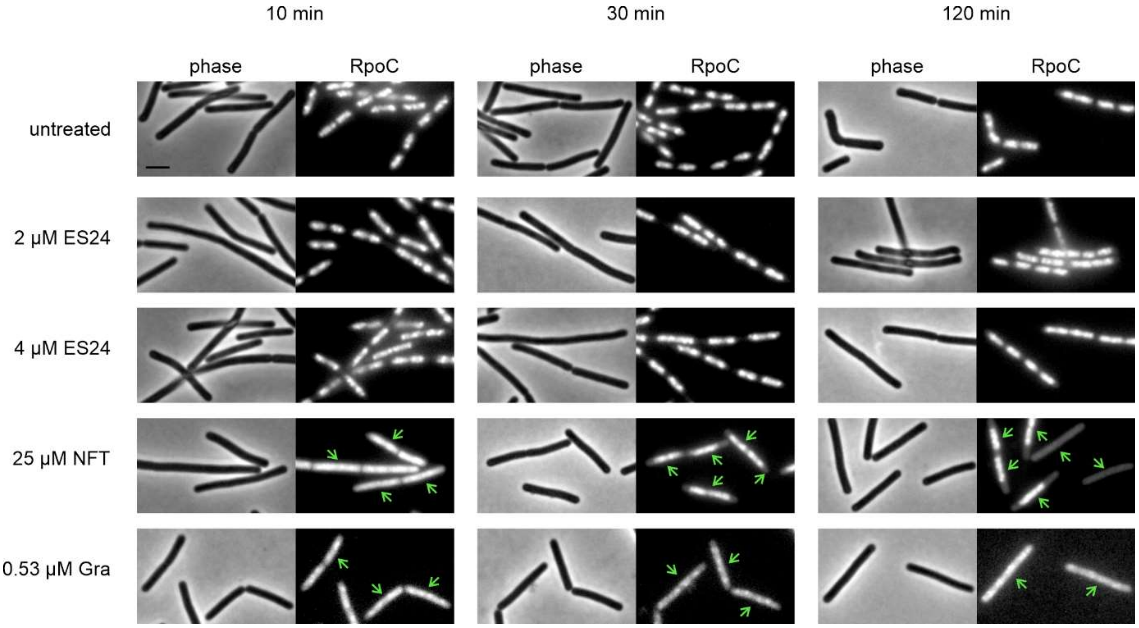

Figure S7: Localization of RpoC-GFP (strain *B. subtilis* 1048) after treatment with ES24 or NFT. Gramicidin (Gra) was used as additional control. Green arrows indicate dispersed protein. Scale bar 2 µm.

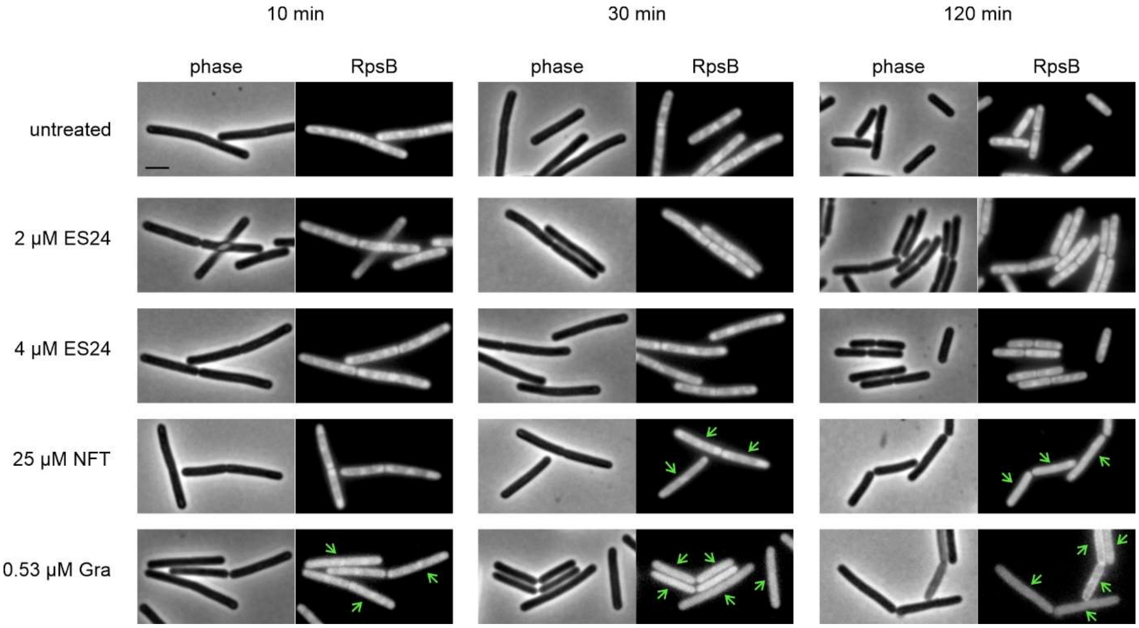

Figure S8: Localization of RpsB-GFP (strain *B. subtilis* 1049) after treatment with ES24 or NFT. Gramicidin (Gra) was used as additional control. Green arrows indicate dispersed protein. Scale bar 2 µm.

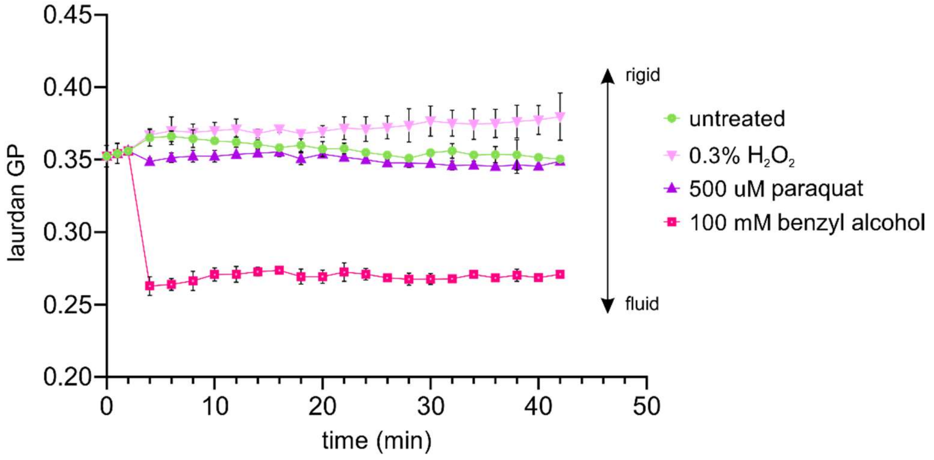

Figure S9: Membrane fluidity of *B. subtilis* 168CA after treatment with peroxide or paraquat. The membrane fluidizer benzyl alcohol was included as positive control.

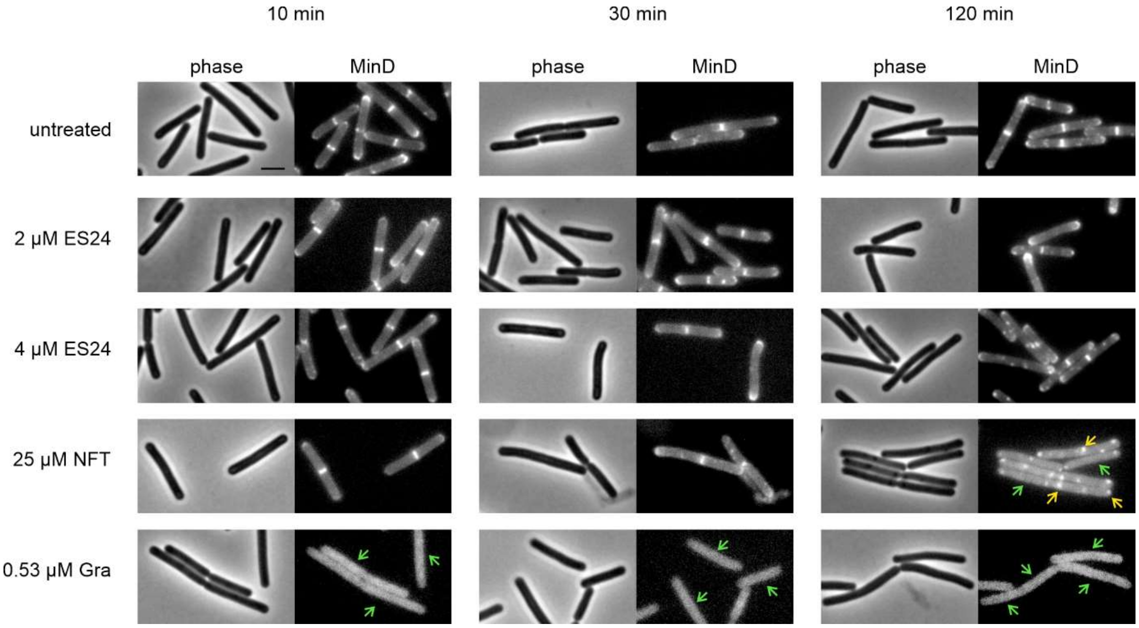

Figure S10: Localization of GFP-MinD (strain *B. subtilis* LH131) after treatment with ES24 or NFT. Gramicidin (Gra) was used as additional control. Green arrows indicate dispersed protein. Yellow arrows indicate clustered protein. Scale bar 2 μm.

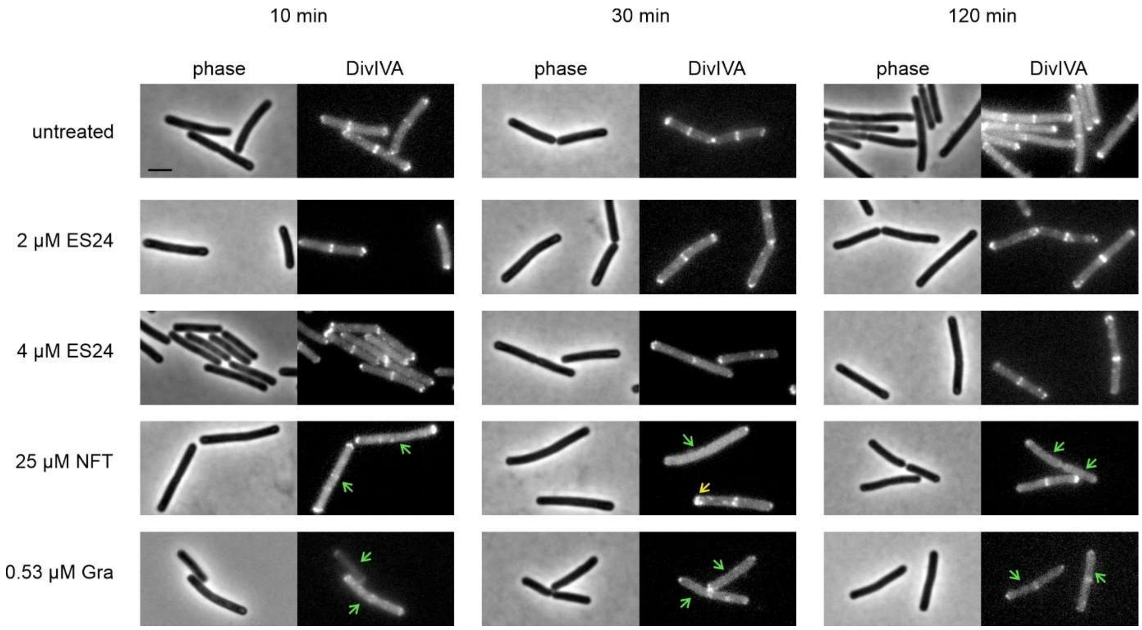

Figure S11: Localization of DivIVA-GFP (strain *B. subtilis* HS63) after treatment with ES24 or NFT. Gramicidin (Gra) was used as additional control. Green arrows indicate dispersed protein. Yellow arrows indicate clustered protein. Scale bar 2 µm.

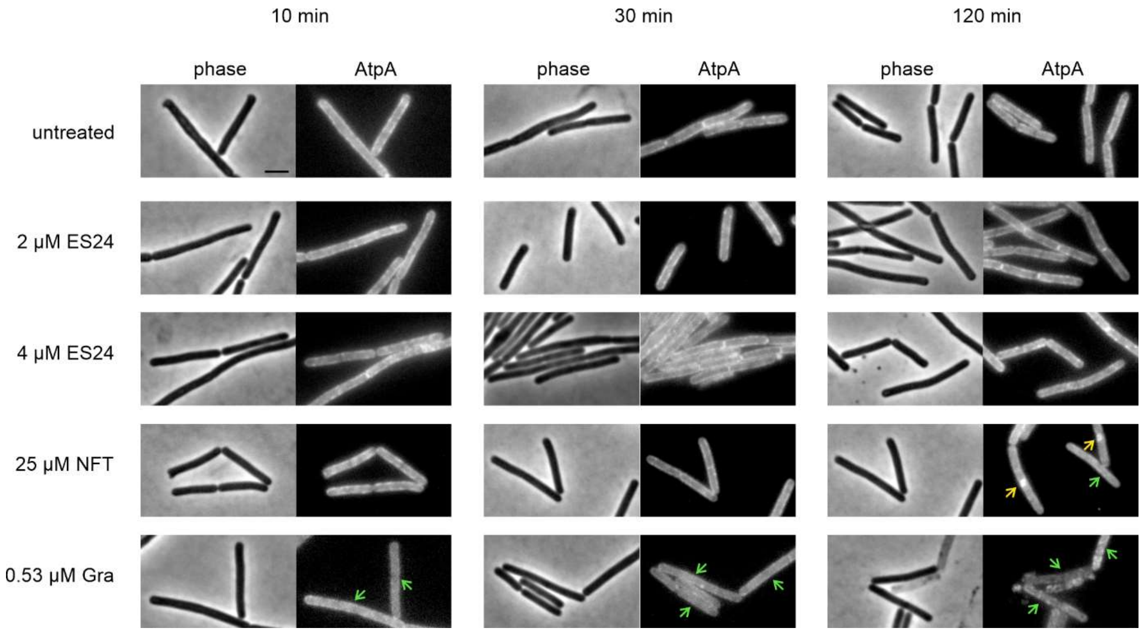

Figure S12: Localization of AtpA-GFP (strain *B. subtilis* BS23) after treatment with ES24 or NFT. Gramicidin (Gra) was used as additional control. Green arrows indicate dispersed protein. Yellow arrows indicate clustered protein. Scale bar 2 µm.

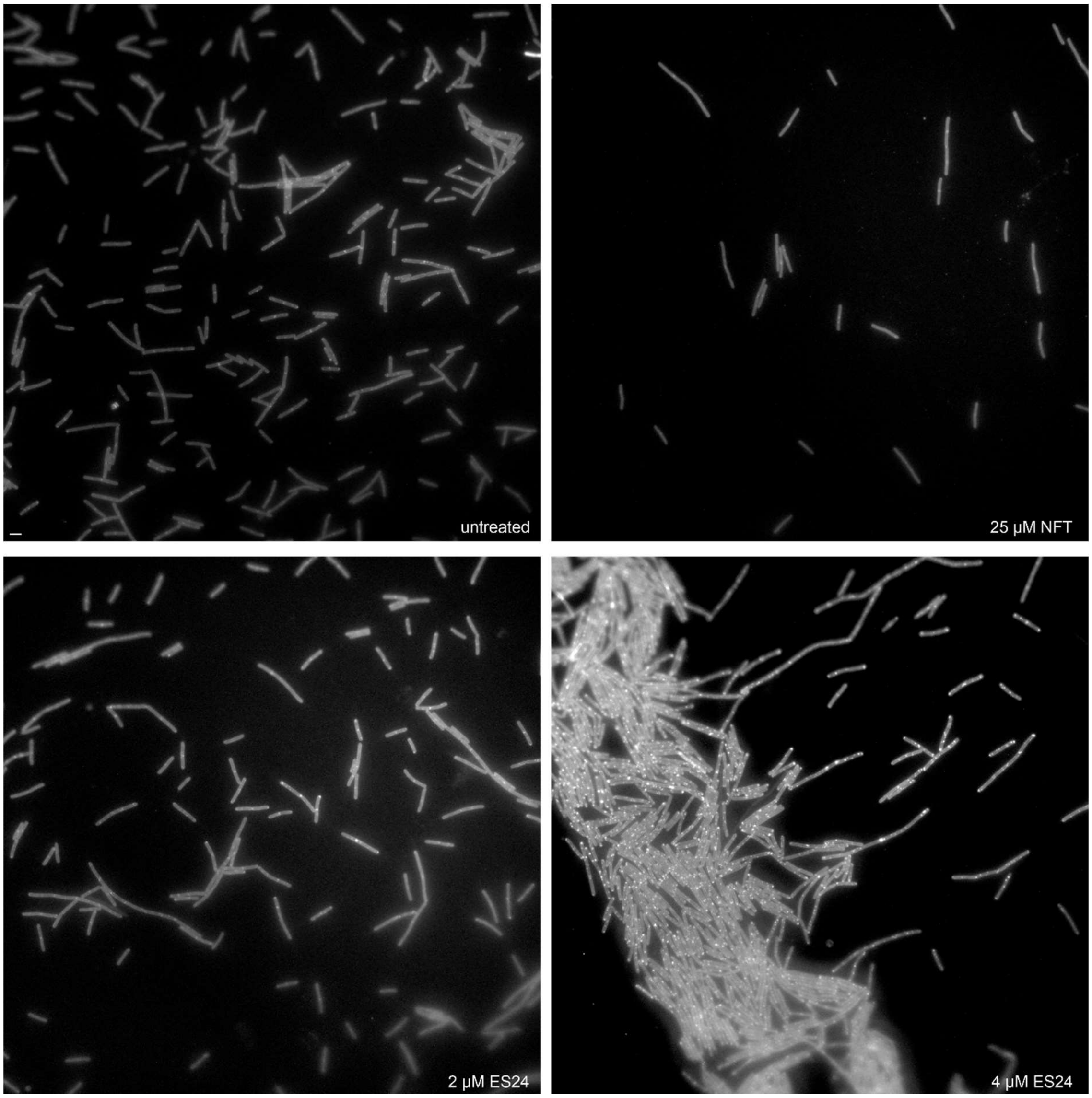

Figure S13: Overview images of *B. subtilis* BSN101 expressing SecA-GFP after 4 h in the absence or presence of ES24 or NFT, respectively.

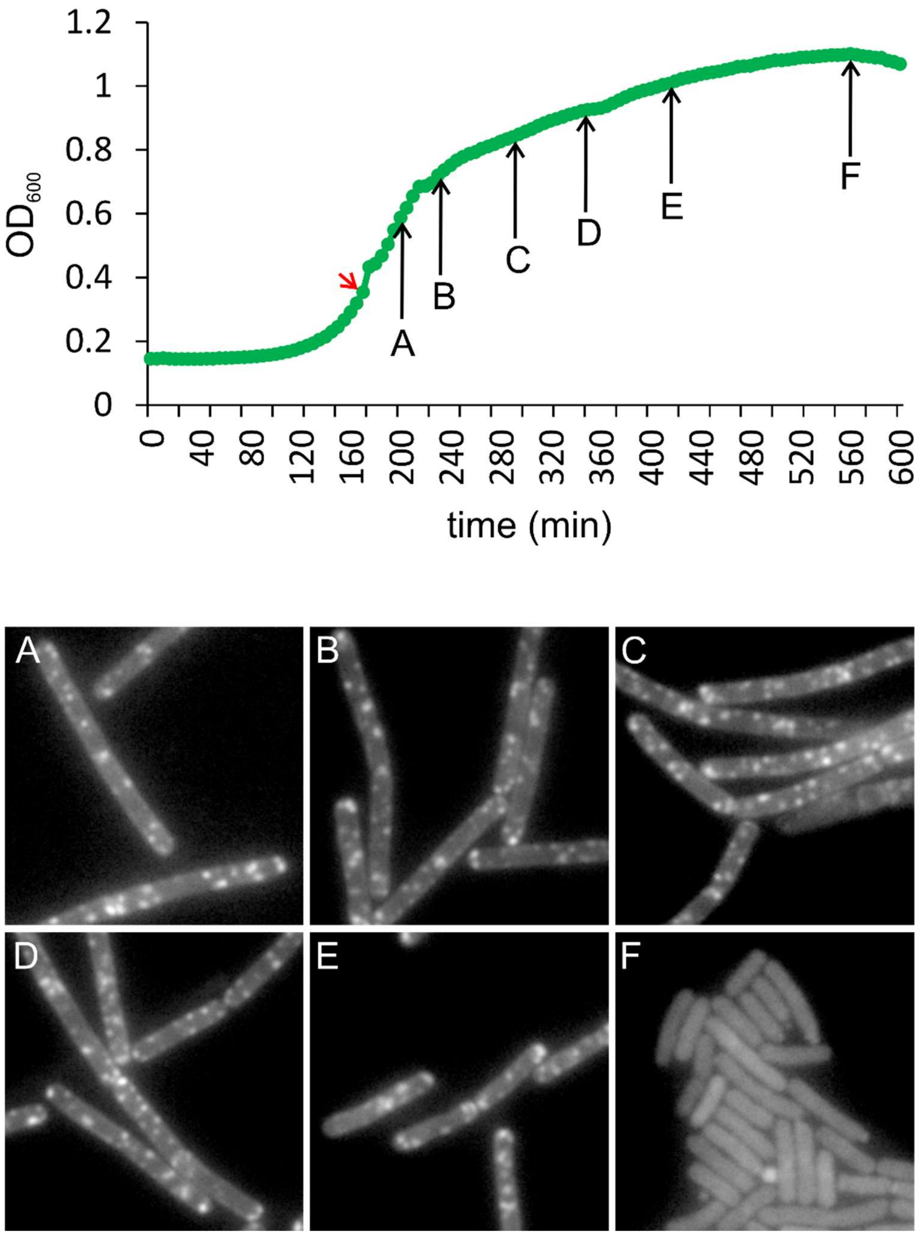

Figure S14: Localization of SecA-GFP at different timepoints in the absence of antibiotics. The red arrow marks the timepoint, at which antibiotics were added in stress experiments. Black arrows indicate timepoints A-F, at which samples were taken for microscopy. Corresponding microscopy images below are labeled accordingly.

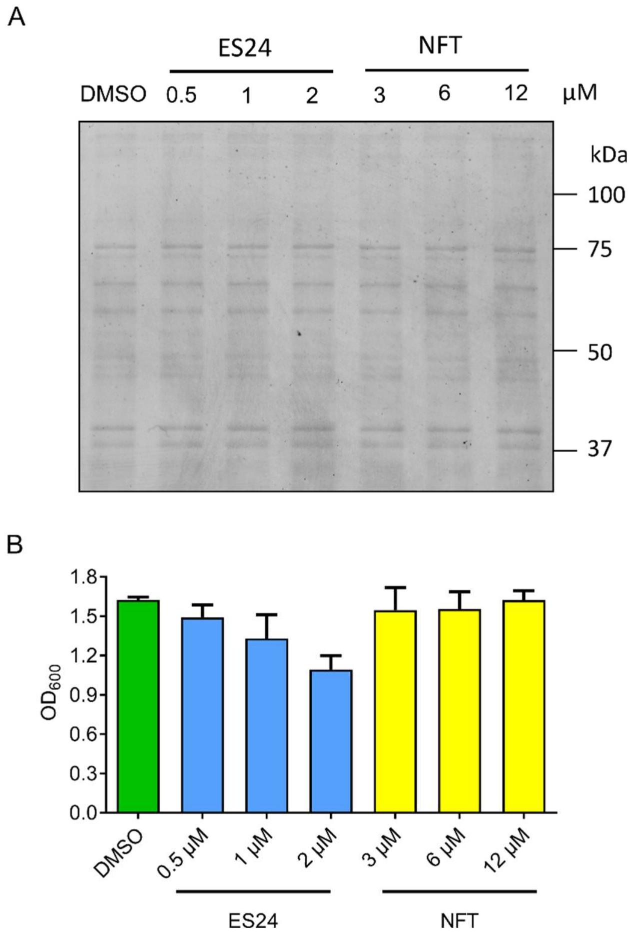

Figure S15: AmyM secretion assay loading controls. **(A)** Coomassie blue staining of the SDS-PAGE gel corresponding to the αAmyM Western blot shown in Figure 7. **(B)** Optical density of cell samples after 3 h of antibiotic treatment. Sample volumes for SDS-PAGE were adjusted based on OD_{600}.

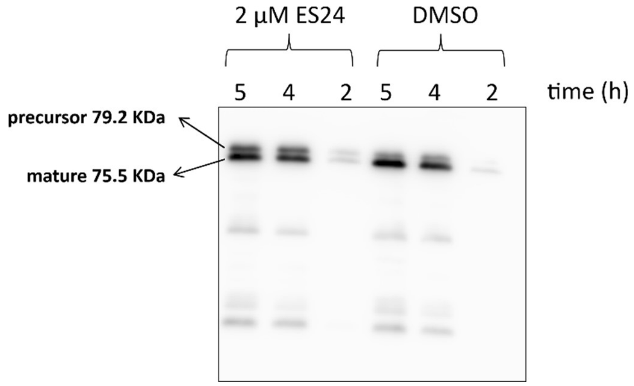

Figure S16: Western blot of cell pellets. *B. subtilis* BWB09 (*ΔxynA ΔamyE*) carrying pCS73 (*PamyQ-amyM*) to express *amyM* from the constitutive *PamyQ* promoter was grown until mid-log phase and treated with 2 µM ES24 or DMSO (negative control) for 4 h prior to SDS-PAGE and Western blotting.

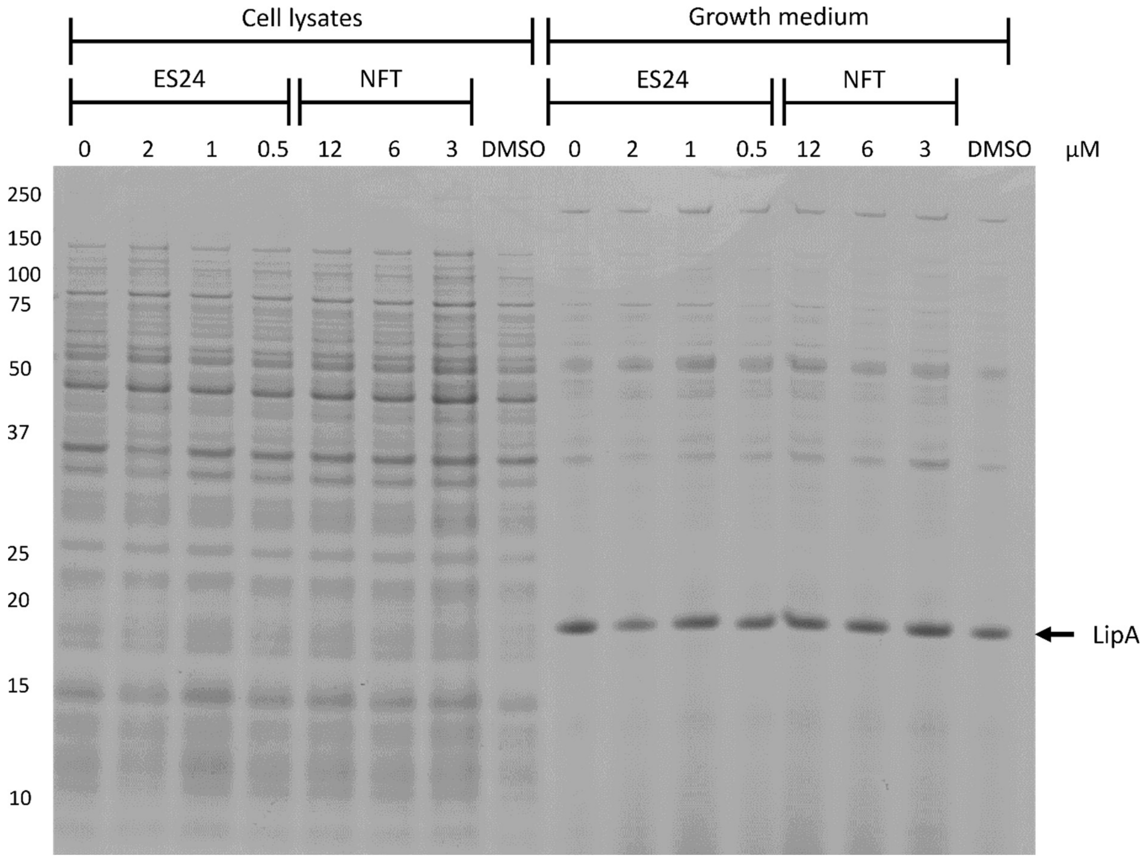

Figure S17: Effects on LipA secretion. LDS-PAGE of cell lysates and culture supernatant (growth medium) of *B. subtilis* TEB1030 (*ΔlipA*) carrying pBSlipA to express the *lipA* gene from the constitutive *PhpaII* promoter. DMSO concentration was 0.5%.

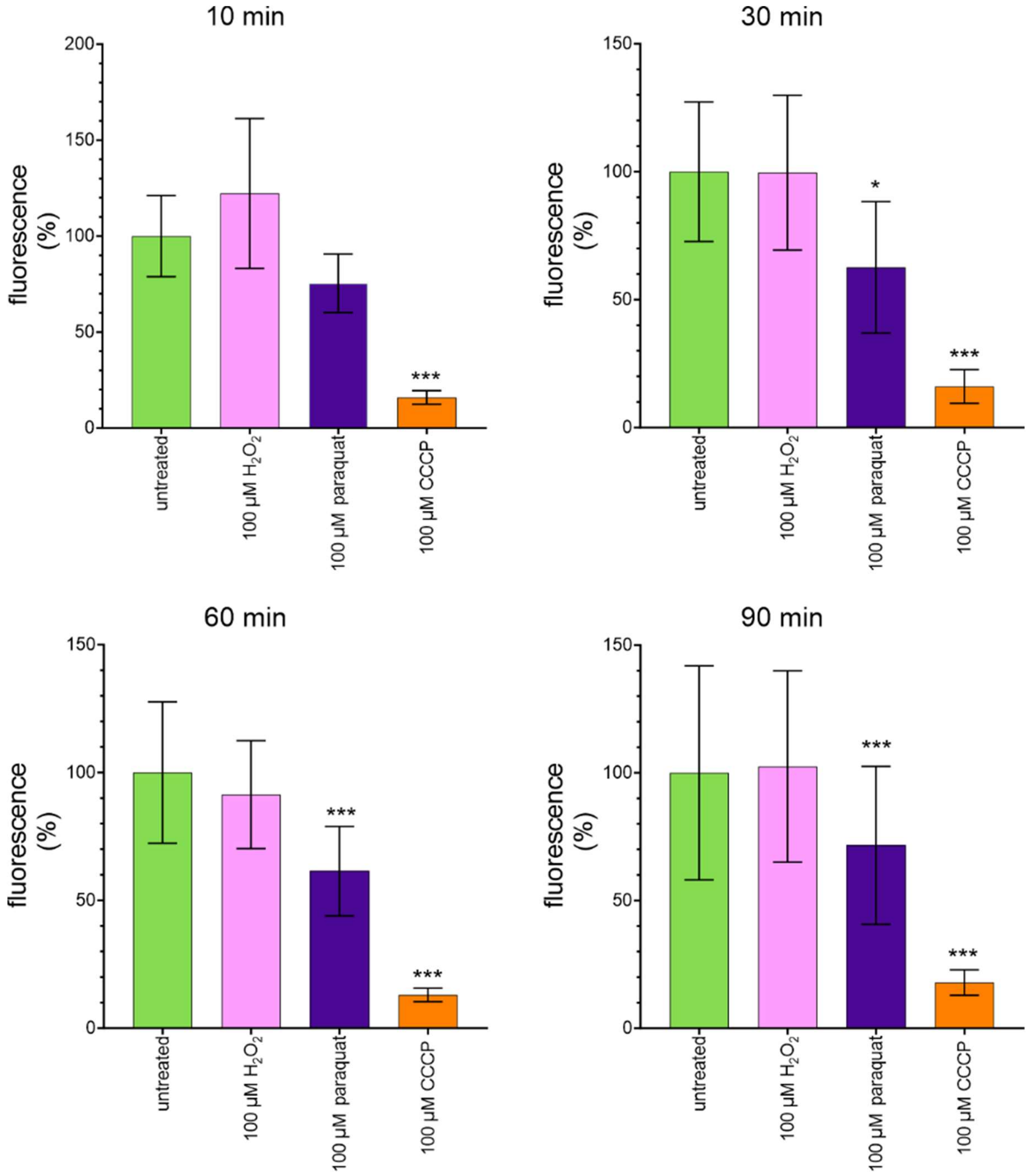

Figure S18: DiSC(3)5 measurements of *E. coli* MC4100 carrying pABCON2-*fhuA* ΔC/Δ4L treated with peroxide, paraquat, or CCCP. p-values were calculated using a heteroscedastic, two-tailed t-test. *$p<0.05$, **$p<0.01$, ***$p<0.001$.

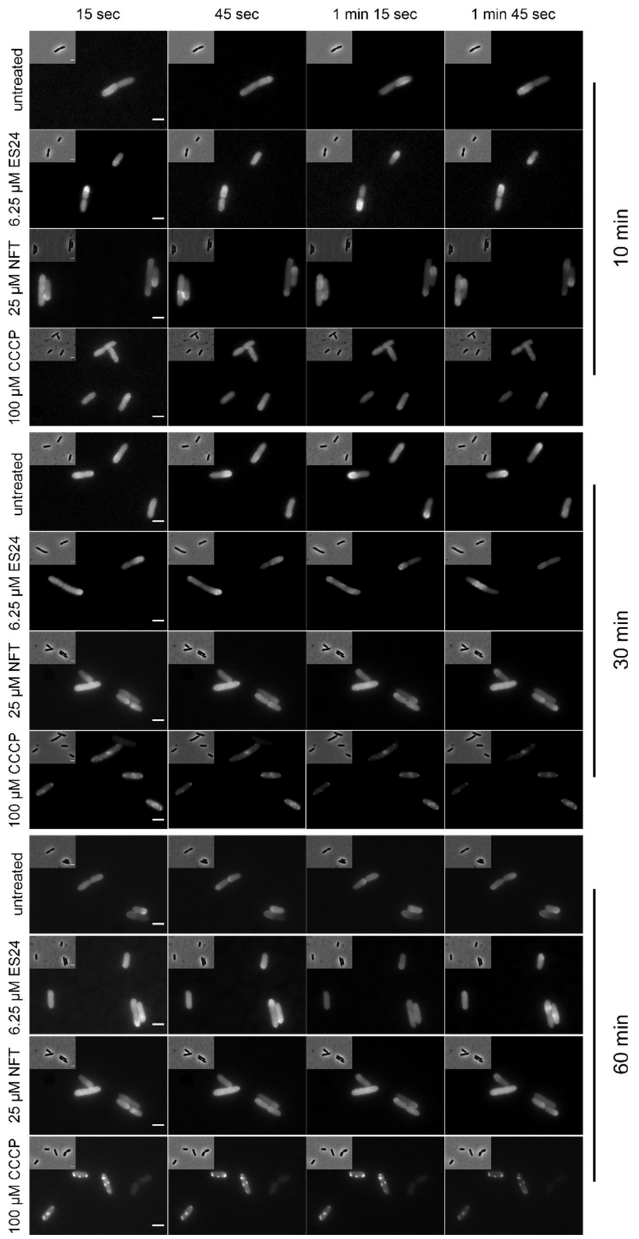

Figure S19: MinD oscillation in *E. coli* RC1 carrying pFX9 treated with ES24 or NFT. The proton ionophore CCCP, which is known to abolish MinD oscillation due to membrane depolarization (1), was used as positive control.

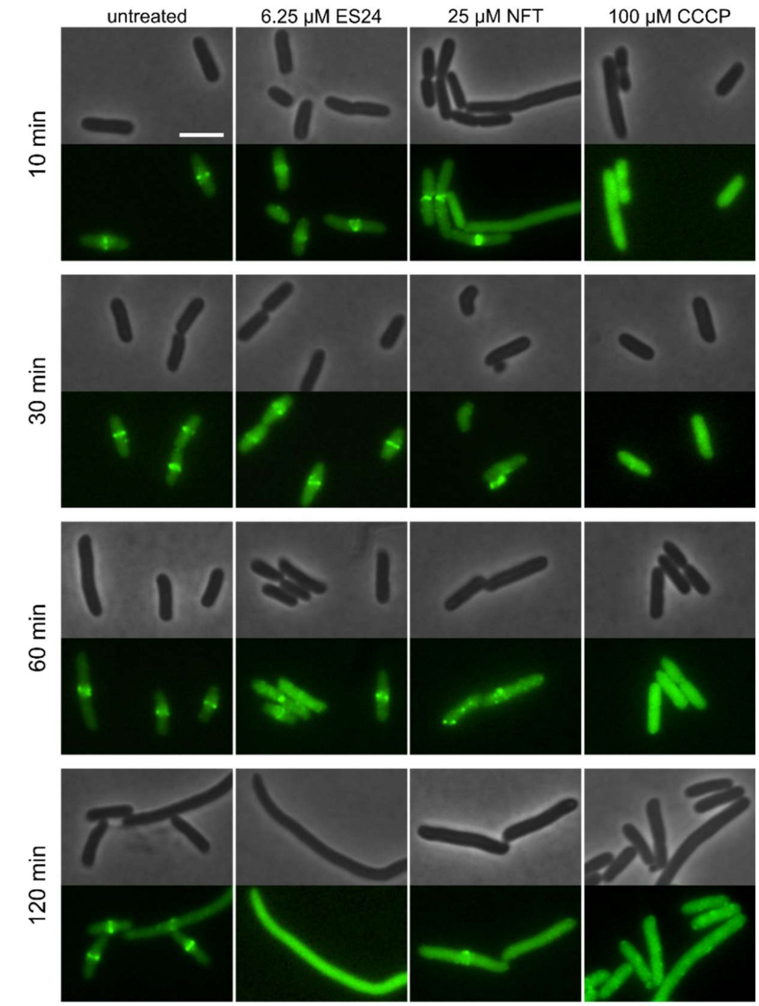

Figure S20: Effects on FtsZ localization in *E. coli*.

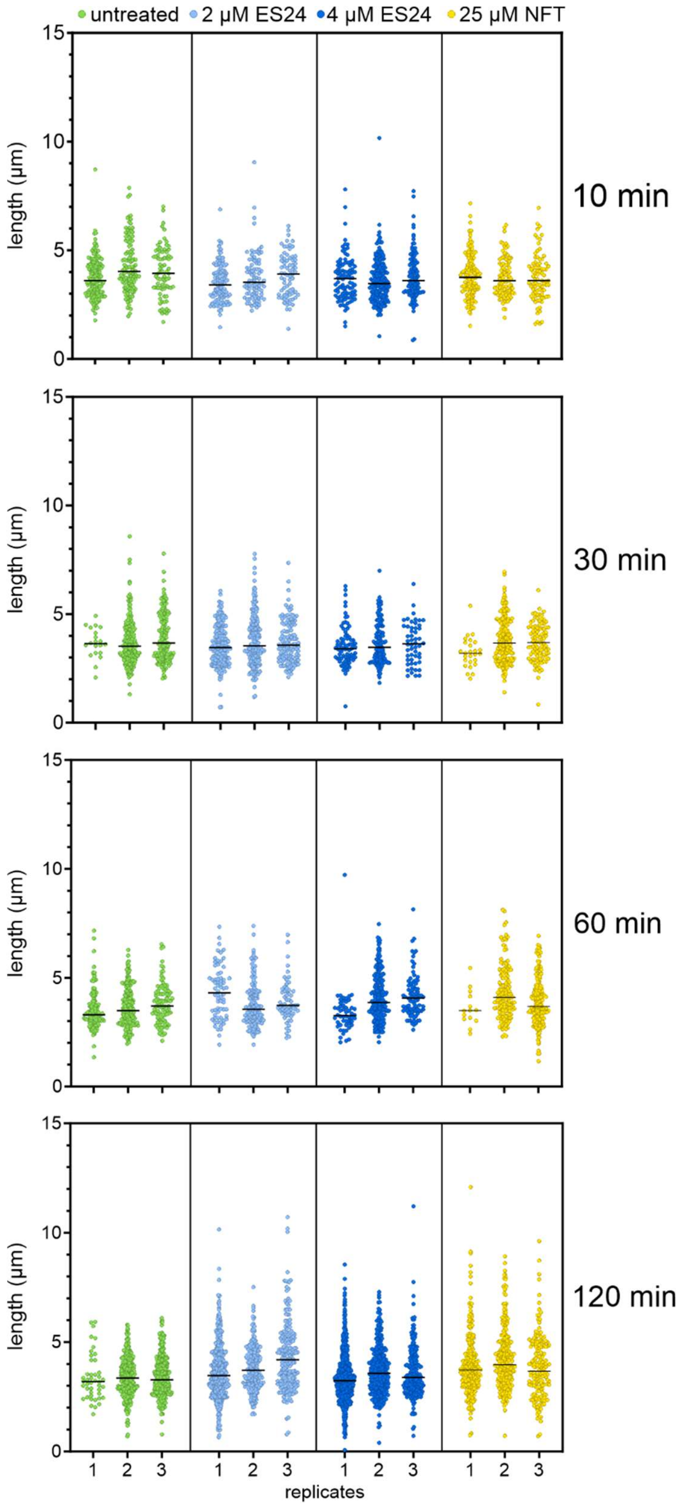

Figure S21: Cell length measurements of *B. subtilis* 168CA treated with ES24 or NFT. Cell length was measured from closed septum to closed septum based on membrane staining with mitotracker green.

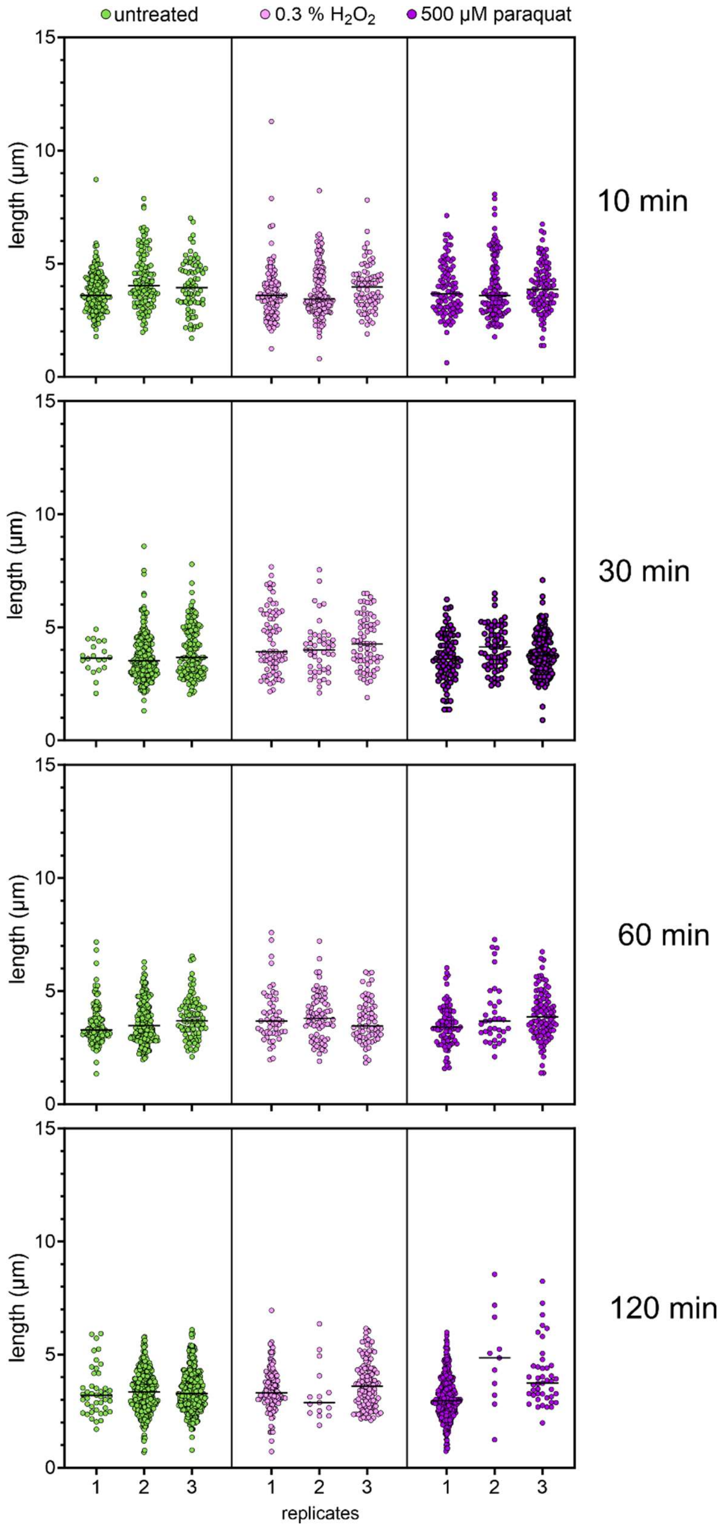

Figure S22: Cell length measurements of *B. subtilis* 168CA treated with peroxide or paraquat. Cell length was measured from closed septum to closed septum based on membrane staining with mitotracker green.

Table S1: Transcripts induced by both ES24 and NFT. IF: induction factor

locus tag	gene name	category	gene product	IF ES24	IF NFT
BSU_33222	*rsoA*	acid stress response	regulator of sigma-O	8.12	3.70
BSU_33230	*sigO*	acid stress response	alternative sigma factor	22.03	17.44
BSU_31920	*adeR*	amino acid metabolism	transcription activator (PucR family) for *ald* gene expression	2.41	2.14
BSU_11250	*argF*	amino acid metabolism	ornithine carbamoyltransferase	2.04	2.12
BSU_39920	*asnH*	amino acid metabolism	asparagine synthetase (glutamine-hydrolyzing)	3.04	2.22
BSU_03890	*gabR*	amino acid metabolism	transcriptional regulator (GntR/MocR family) with PLP binding site (GabR-GABA-PLP aldimine)	4.33	2.01
BSU_03900	*gabT*	amino acid metabolism	4-aminobutyrate aminotransferase	3.94	4.74
BSU_05970	*rex*	anearobic respiration	transcriptional repressor of anaerobically expressed genes involved in anaerobic respiration and fermentation	3.05	2.22
BSU_31590	*yufS*	antibiotic production	putative bacteriocin	2.40	2.12
BSU_21490	*sunI*	antibiotic resistance	protein of immunity to sublancin	4.07	2.99
BSU_19420	*yojK*	antibiotic resistance	putative YDP-glycosyltransferase	3.87	3.26
BSU_39930	*yxaM*	antibiotic resistance	similar to antibiotic resistance protein	2.85	2.60
BSU_18780	*yoaW*	biofilms	biofilm forming exported protein	3.96	3.31
BSU_28810	*abnA*	carbon metabolism	arabinan-endo-1,5-alpha-L-arabinase	3.31	3.34
BSU_39330	*abnB*	carbon metabolism	arabinan endo-1,5-alpha-L-arabinosidase ([Ca(2+)-dependent]	9.21	3.91
BSU_36020	*alsR*	carbon metabolism	transcriptional regulator controlling alsSD, ictEP expression (LysR family)	2.66	2.03
BSU_03040	*amyE*	carbon metabolism	alpha-amylase	2.77	2.08
BSU_28760	*egsA*	carbon metabolism	sn-glycerol-1-phosphate dehydrogenase [NAD+] (catabolic)	2.22	2.03
BSU_40190	*fbp*	carbon metabolism	fructose-1,6-bisphosphatase	2.67	3.09
BSU_03920	*glcU*	carbon metabolism	glucose uptake protein	33.77	25.89
BSU_39780	*iolS*	carbon metabolism	putative aldo-keto reductase	2.62	3.96
BSU_37050	*maeA*	carbon metabolism	NAD-dependent malic enzyme (conversion of malate into pyruvate)	6.90	2.86
BSU_29220	*maeB*	carbon metabolism	NADP-dependent malic enzyme (conversion of malate into pyruvate, anabolic)	3.51	3.90
BSU_01900	*ybcM*	carbon metabolism	similar to glucosamine-fructose-6-phosphate aminotransferase	23.38	4.93
BSU_17860	*dinK*	cell division	cell division inhibitor	16.49	12.78
BSU_34750	*whiA*	cell division	putative morphogen	2.89	2.82
BSU_25900	*cwlA*	cell wall synthesis and turnover	N-acetylmuramoyl-L-alanine amidase; skin element	6.67	4.97
BSU_25710	*cwlH*	cell wall synthesis and turnover	N-acetylmuramoyl-L-alanine amidase	5.27	2.32
BSU_32630	*dadA*	cell wall synthesis and turnover	D-amino acid oxidase	2.03	2.30
BSU_14040	*ltdD*	cell wall synthesis and turnover	murein L,D-transpeptidase	3.79	3.18
BSU_34760	*mgfK*	cell wall synthesis and turnover	gluconeogenesis morphogenetic factor (UDP-sugar binding)	2.36	2.44

BSU_17270	*pghC*	cell wall synthesis and turnover	gamma-polyglutamate hydrolase (phage origin)	11.99	5.34
BSU_35770	*tagC*	cell wall synthesis and turnover	putative polyglycerol phosphate assembly and export protein (teichoic acid biosynthesis)	2.77	2.20
BSU_36230	*uglF*	cell wall synthesis and turnover	UDP-glucose dehydrogenase	6.44	5.59
BSU_18720	*yoaR*	cell wall synthesis and turnover	putative factor for cell wall maintenance or synthesis	4.74	3.17
BSU_22580	*ypiB*	cell wall synthesis and turnover	effector protein of MurAA degradation by ClpC-ClpP	5.45	4.30
BSU_31160	*yubA*	cell wall synthesis and turnover	similar to coordinator of zonal elongation, may control cell wall synthesis by the penicillin-binding proteins	2.92	2.31
BSU_35970	*ywsB*	cell wall synthesis and turnover	putative cell wall binding enzyme	7.34	2.90
BSU_misc_RNA_78	*fswB*	co-factors	flavin mononucleotide riboswitch	2.45	3.15
BSU_10120	*hemE*	co-factors	uroporphyrinogen III decarboxylase	2.54	2.49
BSU_10130	*hemH*	co-factors	ferrochelatase	3.53	3.32
BSU_10140	*hemY*	co-factors	promiscuous protoporphyrinogen IX and coproporphyrinogen III oxidase	3.50	3.70
BSU_36700	*moaA*	co-factors	GTP 3',8-cyclase	3.68	4.45
BSU_05960	*moaC*	co-factors	molybdenum cofactor biosynthesis protein C	3.18	2.21
BSU_15110	*panE*	co-factors	2-dehydropantoate 2-reductase	4.78	3.07
BSU_29540	*ppnKB*	co-factors	inorganic polyphosphate/ATP-NAD kinase	4.36	3.50
BSU_11140	*ribZC*	co-factors	5-amino-6-ribitylamino-2,4(1H, 3H)-pyrimidinedione 5'-phosphate phosphatase (promiscuous)	3.17	2.32
BSU_misc_RNA_14	*tswA*	co-factors	thiamine pyrophosphate riboswitch	8.55	5.76
BSU_27850	*nadA*	co-factors, respiration	quinolinate synthetase	3.56	2.04
BSU_27860	*nadC*	co-factors, respiration	nicotinate-nucleotide pyrophosphorylase (quinolinate phosphoribosyltransferase)	6.52	2.67
BSU_27880	*nifS*	co-factors, respiration	desulfurase involved in iron-sulfur clusters for NAD biosynthesis	2.62	2.14
BSU_11530	*coiA*	competence	protein involved in establishment of DNA transport in competence	34.95	7.26
BSU_24730	*comGA*	competence	membrane associated ATPase of the pilin platform for DNA competence	3.86	2.36
BSU_34770	*yvcJ*	competence	GTPase possibly involved in regulator sRNA degradation	2.60	2.48
BSU_25790	*arsB*	detoxification	arsenite efflux transporter; skin element	20.11	14.82
BSU_25810	*arsR*	detoxification	transcriptional regulator (ArsR-arsenate); skin element	35.79	15.47
BSU_27020	*sufL*	detoxification	deglycase; general stress protecting enzyme; protects against methylglyoxal toxicity	15.54	14.75
BSU_40780	*tetL*	detoxification	tetracycline resistance leader peptide	14.17	7.57
BSU_02880	*yceB*	detoxification	putative luciferase-like monooxygenase	32.26	11.47
BSU_32810	*yusI*	detoxification	putative oxidoreductase with thioredoxin domain and regulator domain	3.25	4.79
BSU_39540	*yxeI*	detoxification	penicillin V amidase	14.29	6.09
BSU_40740	*yyaR*	detoxification	putative acetyl-transferase	19.53	10.45
BSU_06060	*bsuMA*	DNA repair	DNA-methyltransferase (cytosine-specific); prophage 3 region	5.69	4.26
BSU_06070	*bsuMB*	DNA repair	DNA-methyltransferase (cytosine-specific); defective prophage 3	6.01	4.71

BSU_17870	*dinL*	DNA repair	putative site-specific recombinase, resolvase	16.27	13.47
BSU_13540	*ogt*	DNA repair	O6-alkylguanine DNA alkyltransferase	3.15	3.20
BSU_23870	*polYA*	DNA repair	DNA-damage lesion bypass DNA polymerase	4.46	2.94
BSU_23710	*polYB*	DNA repair	Y family DNA polymerase V bypassing lesions during replication	26.94	13.80
BSU_00870	*radA*	DNA repair	DNA repair protein; 6-O-methylguanine-DNA methyltransferase	5.55	2.34
BSU_16940	*recA*	DNA repair	multifunctional SOS repair factor	3.93	4.68
BSU_09920	*sbcE*	DNA repair	ATPase involved in DNA double strand break repair and recombination	2.80	4.34
BSU_35160	*uvrA*	DNA repair	excinuclease ABC (subunit A)	16.36	12.85
BSU_35170	*uvrB*	DNA repair	excinuclease ABC (subunit B)	20.42	11.75
BSU_28490	*uvrC*	DNA repair	excinuclease ABC (subunit C)	6.99	4.75
BSU_21500	*uvrX*	DNA repair	lesion bypass phage DNA polymerase; phage SPbeta	28.08	16.60
BSU_09910	*yhaO*	DNA repair	putative DNA repair exonuclease	3.37	4.45
BSU_09810	*yhaZ*	DNA repair	alkyl purine DNA glycosylase	16.72	9.57
BSU_10470	*yhjD*	DNA repair	conserved hypothetical protein	17.22	10.09
BSU_10480	*yhjE*	DNA repair	putative integral membrane protein	2.22	2.16
BSU_13800	*ykvR*	DNA repair	conserved hypothetical protein (HGT island)	9.09	4.30
BSU_15050	*ylbL*	DNA repair	putative degradative enzyme	2.59	2.32
BSU_17880	*ynzC*	DNA repair	conserved protein of unknown function	3.23	3.35
BSU_20500	*yoqV*	DNA repair	DNA ligase-like protein; bacteriophage SPbeta	11.05	5.07
BSU_20440	*yorB*	DNA repair	conserved hypothetical protein; phage SPbeta	6.32	6.05
BSU_20430	*yorC*	DNA repair	conserved protein of unknown function; phage SPbeta	7.13	9.99
BSU_23700	*yqjX*	DNA repair	conserved protein of unknown function	14.82	11.32
BSU_23660	*yqkB*	DNA repair	conserved protein of unknown function	5.73	3.38
BSU_23630	*yqkE*	DNA repair	conserved protein of unknown function	5.69	3.73
BSU_34740	*crh*	gene regulation	catabolite repression HPr-like protein	8.89	4.54
BSU_03850	*ycnC*	gene regulation	putative transcriptional regulator (TetR/AcrR family)	9.50	5.12
BSU_03880	*yczG*	gene regulation	putative transcriptional regulator (ArsR family)	3.63	2.48
BSU_05370	*ydfD*	gene regulation	putative PLP-dependent transcriptional regulator	4.74	2.25
BSU_05270	*ydzF*	gene regulation	putative transcriptional regulator	4.09	3.99
BSU_08410	*yfiV*	gene regulation	putative transcriptional regulator (MarR family)	2.02	2.00
BSU_13870	*ykvZ*	gene regulation	putative transcriptional regulator (LacI family)	2.51	2.92
BSU_32669	*yuzN*	gene regulation	putative transcriptional regulator	6.10	3.84
BSU_36440	*ywoH*	gene regulation	putative transcriptional regulator (MarR family)	3.12	3.47
BSU_39850	*yxbF*	gene regulation	putative transcriptional regulator	3.33	3.24
BSU_33400	*pgoN*	general stress response	promiscuous glyoxal/methylglyoxal reductase	5.32	7.14
BSU_04740	*rsbX*	general stress response	serine phosphatase	2.88	2.22
BSU_14670	*suhB*	general stress response	inositol monophosphatase / 5' nucleotidase (purine nucleoside monophosphate)	5.50	2.99
BSU_00890	*yacL*	general stress response	putative membrane protein possibly involved in RNA binding	5.87	2.08

BSU_08570	*yfhK*	general stress response	putative exported protein	6.53	2.44
BSU_07680	*yflH*	general stress response	putative enzyme	2.23	2.57
BSU_14680	*ykzC*	general stress response	putative acyltransferase	3.97	2.90
BSU_28340	*ysnF*	general stress response	putative stress response protein	2.54	2.10
BSU_29410	*ytkL*	general stress response	putative metal-dependent hydrolase	3.84	3.40
BSU_36720	*ywmE*	general stress response	conserved protein of unknown function	2.87	3.32
BSU_40210	*yydC*	general stress response	conserved hypothetical protein	3.40	3.22
BSU_00830	*ctsR*	heat shock	transcriptional regulator of class III stress genes	20.10	6.35
BSU_06020	*groES*	heat shock	chaperonin small subunit	2.00	3.86
BSU_25490	*hrcA*	heat shock	transcriptional regulator of heat-shock genes	3.85	2.89
BSU_07550	*yflT*	heat shock	heat stress induced protein	5.44	3.50
BSU_40529	*yyzH*	heat shock	hypothetical protein	11.91	3.70
BSU_00840	*mcsA*	heat shock/sporulation	activator of protein kinase McsB	12.97	4.85
BSU_33490	*cadA*	ion homeostasis	Cd(II), Zn(II) and Co(II) exporter (ATPase)	38.80	9.44
BSU_33500	*copA*	ion homeostasis	copper transporter ATPase	6.33	2.11
BSU_33510	*copZ*	ion homeostasis	copper insertion chaperone and transporter component	8.50	2.26
BSU_31322	*kbfO*	ion homeostasis	potassium channel protein involved in biofilm formation	3.75	2.11
BSU_09870	*khtS*	ion homeostasis	K+/H+ antiporter for K+ efflux	14.57	11.90
BSU_09860	*khtT*	ion homeostasis	K+/H+ antiporter for K+ efflux	16.86	10.89
BSU_36030	*ywrK*	ion homeostasis	putative Na+/H+ antiporter	4.87	3.62
BSU_09850	*khtU*	ion homeostasis, detoxification	proton/potassium antiporter; methylglyoxal resistance	15.46	11.44
BSU_32160	*sufA*	iron-sulfur clusters, heme	sulfur carrier chaperone involved in Fe-S cluster assembly	3.47	3.32
BSU_11160	*yitW*	iron-sulfur clusters, heme	putative protein involved in Fe-S cluster assembly, PaaD-like	3.37	3.09
BSU_37170	*acdA*	lipid metabolism	acyl-CoA dehydrogenase	4.28	4.10
BSU_36590	*clsA*	lipid metabolism	cardiolipin synthase (major)	2.32	2.34
BSU_24180	*glpQ*	lipid metabolism	glycerophosphodiester phosphodiesterase (exolytic cleavage of individual teichoic acid monomer units)	3.96	3.29
BSU_09240	*glpW*	lipid metabolism	promiscuous phosphoglycolate phosphatase / glycerol-3-phosphate phosphatase / 2-deoxyglucose-6-phosphatase	3.47	3.47
BSU_10340	*phoE*	lipid metabolism	promiscuous phosphatase; putative (phosphoglycerate) mutase	6.68	4.25
BSU_32850	*putM*	lipid metabolism	fatty acid degradation, similar to proline dehydrogenase	4.50	3.13
BSU_29560	*ytcI*	lipid metabolism	putative acyl-coenzyme A synthetase	3.82	5.06
BSU_29420	*ytkK*	lipid metabolism	putative 3-oxoacyl-acyl-carrier protein reductase	3.86	3.31
BSU_33590	*yvaG*	lipid metabolism	similar to 3-oxoacyl- acyl-carrier protein reductase	5.88	2.46
BSU_29060	*coaE*	lipid metabolism, co-factor	dephosphocoenzyme A kinase	2.23	2.45
BSU_00700	*coaX*	lipid metabolism, co-factor	pantothenate kinase type III	7.76	3.36
BSU_09640	*mscY*	membrane stress	small conductance mechano-sensitive channel	2.73	2.87

BSU_30500	*tbcS*	membrane stress	tetraprenyl-beta-curcumene synthase	2.52	2.95
BSU_06380	*yebC*	membrane stress	putative integral inner membrane protein	5.24	2.79
BSU_18500	*yoxD*	membrane stress	putative oxido-reductase	2.43	5.05
BSU_23300	*ypuD*	membrane stress	putative intramembrane metalloprotease	12.70	2.74
BSU_30510	*ytpA*	membrane stress	phospholipase component of bacilysocin synthesis or export	2.43	2.88
BSU_25060	*yqfZ*	motility/chemotaxis	factor involved in motility	3.73	3.21
BSU_misc_RNA_8	*aswA*	nucleotide metabolism	adenine riboswitch	5.59	3.62
BSU_misc_RNA_34	*gswA*	nucleotide metabolism	guanine riboswitch	2.08	2.24
BSU_32130	*guaC*	nucleotide metabolism	GMP reductase (NADP-dependent)	2.20	2.83
BSU_21890	*hdhQ*	nucleotide metabolism	Mn(2+)-dependent (deoxy)ribonucleoside pyrophosphohydrolase	2.11	2.40
BSU_17380	*nrdE*	nucleotide metabolism	ribonucleoside-diphosphate reductase (major subunit)	2.65	2.41
BSU_17390	*nrdF*	nucleotide metabolism	ribonucleoside-diphosphate reductase (minor subunit)	2.66	2.66
BSU_17370	*nrdI*	nucleotide metabolism	co-factor of ribonucleotide diphosphate reductase	2.99	2.21
BSU_15520	*pyrAB*	nucleotide metabolism	pyrimidine-specific carbamoyl-phosphate synthetase (large subunit)	8.47	2.56
BSU_15540	*pyrD*	nucleotide metabolism	dihydroorotate dehydrogenase (catalytic subunit)	7.34	3.13
BSU_15560	*pyrE*	nucleotide metabolism	orotate phosphoribosyltransferase	8.75	3.89
BSU_15550	*pyrF*	nucleotide metabolism	orotidine 5'-phosphate decarboxylase	7.05	3.16
BSU_17400	*ymaB*	nucleotide metabolism	putative cofactor involved in deoxyribonucleotide synthesis	2.33	2.87
BSU_31050	*gbsB*	osmoregulation	choline dehydrogenase	2.55	2.23
BSU_13860	*papB*	osmoregulation	Xaa-Pro Xaa-Pro-Xaa di-tri-peptidase used in osmoprotection	2.37	2.46
BSU_27010	*adhA*	oxidative stress	putative aldehyde dehydrogenase; carbonyl stress response	4.47	4.39
BSU_27000	*adhR*	oxidative stress	transcriptional regulator regulated by thiol-alkylation	2.19	2.01
BSU_25780	*arsC*	oxidative stress	thioredoxin-coupled arsenate reductase; skin element	28.63	19.70
BSU_19230	*azoJ*	oxidative stress	FMN-dependent NADH-azoreductase	6.08	5.66
BSU_33540	*azoRB*	oxidative stress	NADH:dichloroindophenol oxidoreductase (2-methylhydroquinone resistance)	2.70	2.14
BSU_31370	*bdhJ*	oxidative stress	NADH-dependent butanol dehydrogenase	7.46	7.05
BSU_19460	*bshBB*	oxidative stress	malate N-acetylglucosamine deacetylase (second enzyme)	3.22	4.15
BSU_15120	*bshC*	oxidative stress	malate glucosamine cysteine ligase	6.82	6.31
BSU_29310	*cmoJ*	oxidative stress	monooxygenase (S-alkyl substrates)	3.73	2.35
BSU_05630	*dinB*	oxidative stress	nuclease inhibitor	3.97	5.53
BSU_03910	*gabD*	oxidative stress	succinate-semialdehyde dehydrogenase	19.67	19.23
BSU_32660	*glxB*	oxidative stress	methylglyoxalase; lactoylbacillithiol lyase	3.22	2.95
BSU_36100	*hpxW*	oxidative stress	oxamate amidohydrolase	5.59	2.12
BSU_00710	*hslO*	oxidative stress	disulfide bond chaperone (heat shock protein HSP33)	5.17	3.12
BSU_03470	*hxlR*	oxidative stress	positive regulator of hxlAB expression (formaldehyde sensing)	17.90	6.23
BSU_40540	*hypR*	oxidative stress	transcriptional regulator (Cys-activated by oxidative stress)	13.82	3.25
BSU_08820	*katA*	oxidative stress	vegetative catalase 1	29.09	2.23

BSU_12870	*mhqA*	oxidative stress	hydroquinone-specific extradiol dioxygenase	3.56	4.71
BSU_21690	*msrA*	oxidative stress	peptide methionine S-sulfoxide reductase	5.23	4.26
BSU_21680	*msrB*	oxidative stress	peptide methionine R-sulfoxide reductase	3.51	3.32
BSU_29640	*msrC*	oxidative stress	free methionine-(R)-sulfoxide reductase	3.23	2.15
BSU_37270	*narH*	oxidative stress	nitrate reductase (beta subunit)	2.18	2.43
BSU_37260	*narJ*	oxidative stress	nitrate reductase molybdenum cofactor assembly chaperone NarJ	3.43	3.18
BSU_19550	*noxC*	oxidative stress	water forming NADH oxidase (nitroreductase)	2.63	2.61
BSU_14730	*sigP*	oxidative stress	RNA polymerase ECF-type sigma factor	2.90	3.00
BSU_14740	*sigQ*	oxidative stress	anti-SigP(YlaC) sigma factor	2.72	2.57
BSU_11550	*spxH*	oxidative stress	thiol management effector of SpxA degradation	5.98	4.11
BSU_08860	*ssuD*	oxidative stress	FMNH2-dependent aliphatic sulfonate monooxygenase	5.98	3.32
BSU_28500	*trxA*	oxidative stress	thioredoxin	3.74	5.52
BSU_03870	*ycnE*	oxidative stress	putative AI-2 degrading enzyme	43.64	109.04
BSU_04550	*ydbP*	oxidative stress	putative thioredoxin or thiol-disulfide isomerase	2.12	2.97
BSU_05510	*ydfQ*	oxidative stress	putative thioredoxin or thiol-disulfide isomerase	66.15	17.93
BSU_05660	*ydgI*	oxidative stress	nitroreductase of unidentified specificity (reduces 5-(aziridin-1-yl)-2,4-dinitrobenzamide prodrug)	25.32	4.20
BSU_05670	*ydgJ*	oxidative stress	putative transcriptional regulator (MarR family)	35.71	4.73
BSU_08560	*yfhJ*	oxidative stress	conserved hypothetical protein	12.47	4.56
BSU_07990	*yfjR*	oxidative stress	putative beta-hydroxyacid dehydrogenase	12.79	6.93
BSU_11560	*yjbI*	oxidative stress	putative thiol management oxidoreductase component	9.92	4.55
BSU_13770	*ykvO*	oxidative stress	putative oxidoreductase (HGT island)	16.13	2.08
BSU_17280	*ymaD*	oxidative stress	putative peroxiredoxin-related protein	6.58	9.67
BSU_18790	*yoaZ*	oxidative stress	putative factor of the oxidative stress response	3.21	3.45
BSU_19560	*yodD*	oxidative stress	may be involved in protection against methyl-hydroquinone	2.53	2.84
BSU_19570	*yodE*	oxidative stress	putative thiol-dependent lyase/dioxygenase	3.00	2.90
BSU_19470	*yojF*	oxidative stress	conserved protein of unknown function (bacillithiol synthesis operon)	4.33	4.68
BSU_19479	*yoyC*	oxidative stress	conserved protein of unknown function	3.90	4.52
BSU_22500	*ypjD*	oxidative stress	oxidized nucleotide pyrophosphohydrolase	2.32	2.31
BSU_25800	*yqcK*	oxidative stress	putative thiol lyase	19.46	11.34
BSU_27820	*yrbC*	oxidative stress	putative factor regulating gene expression	14.55	8.60
BSU_26540	*yrkE*	oxidative stress	putative protein involved in sulfur metabolism (DsrE-like)	12.33	3.66
BSU_26530	*yrkF*	oxidative stress	putative rhodanese-related sulfur transferase	18.69	3.33
BSU_27009	*yrzP*	oxidative stress	putative carboxymuconolactone decarboxylase	22.63	18.90
BSU_31030	*yuaE*	oxidative stress	bacillithiol S-transferase	7.72	4.99
BSU_31830	*yueE*	oxidative stress	putative metal-dependent phosphohydrolase	15.99	2.49
BSU_33980	*yvbT*	oxidative stress	putative alkanal monooxygenase	25.63	29.19
BSU_33190	*yvrD*	oxidative stress	putative oxidoreductase	15.76	14.59
BSU_38100	*ywcH*	oxidative stress	putative monooxygenase	15.09	15.38
BSU_36580	*ywnF*	oxidative stress	conserved protein of unknown function	33.63	9.17

BSU_35990	*ywrO*	oxidative stress	nitroreductase (unknown substrate)	5.73	4.02
BSU_06040	*ydiM*	oxidative stress, heat shock	hypothetical protein; prophage 3 region	11.88	9.19
BSU_27785	*yrzF*	oxidative stress, sporulation	putative serine/threonine-protein kinase	13.22	5.97
BSU_05600	*ydgE*	protein modification	putative N-acetyltransferase	3.74	3.31
BSU_10260	*yhfK*	protein modification	putative NAD-binding epimerase / hydratase	2.32	3.68
BSU_22570	*ypiF*	protein modification	conserved protein of unknown function	8.55	3.73
BSU_28190	*engB*	protein synthesis	GTPase involved in ribosome 50S subunit assembly (maturation of the central 50S protuberance)	2.29	2.21
BSU_33450	*helD*	protein synthesis	transcription factor	2.48	2.87
BSU_08880	*rpsNB*	protein synthesis	alternative ribosomal protein S14	20.57	7.68
BSU_23540	*yqkK*	protein synthesis	conserved protein of unknown function	6.74	7.24
BSU_36910	*ywlG*	protein synthesis	conserved protein of unknown function	4.02	2.23
BSU_10300	*aprE*	proteolysis	serine alkaline protease (Ca(2+)-dependent subtilisin E)	4.30	3.71
BSU_17260	*aprX*	proteolysis	alkaline serine protease	55.96	12.70
BSU_00860	*clpC*	proteolysis	class III stress response-related ATPase, AAA+ superfamily	10.77	6.87
BSU_13700	*clpE*	proteolysis	ATP-dependent Clp protease (class III stress gene)	80.38	28.25
BSU_34540	*clpP*	proteolysis	ATP-dependent Clp protease proteolytic subunit; Maxwell's demon	3.12	3.58
BSU_28200	*lonA*	proteolysis	class III heat-shock ATP-dependent LonA protease	2.49	2.23
BSU_07690	*mapB*	proteolysis	methionine aminopeptidase B	2.08	2.78
BSU_00850	*mcsB*	proteolysis	protein arginine kinase	11.28	5.22
BSU_11540	*pepF*	proteolysis	oligoendopeptidase F	2.89	3.84
BSU_10490	*sipV*	proteolysis	type I signal peptidase	2.57	2.29
BSU_15310	*spoIIGA*	proteolysis	protease processing pro-sigma-E	2.73	2.76
BSU_38090	*vpr*	proteolysis	extracellular serine protease	10.74	3.84
BSU_12500	*xkdA*	proteolysis	phage PBSX; putative peptidase	6.14	3.64
BSU_01140	*ybaC*	proteolysis	putative proline iminopeptidase	2.62	2.00
BSU_16860	*ymfH*	proteolysis	putative processing protease	2.04	3.40
BSU_20490	*yoqW*	proteolysis	putative stress-associated peptidase; putative general secretion pathway protein; phage SPbeta	15.90	4.97
BSU_26380	*yqaB*	proteolysis	putative peptidase; skin element	6.31	2.74
BSU_31310	*yugP*	proteolysis	putative metal-dependent protease/peptidase	16.37	7.46
BSU_11520	*mecA*	proteolysis, sporulation	adaptor protein controlling oligomerization of the AAA+ protein ClpC	9.26	2.94
BSU_29230	*dnaEC*	replication	DNA polymerase III (alpha subunit), DnaE3	3.65	3.86
BSU_18490	*rtp*	replication	replication terminator protein	6.75	4.26
BSU_01830	*ndhF*	respiration	putative NADH dehydrogenase; prophage 1 region	3.69	2.34
BSU_30710	*ythA*	respiration	putative cytochrome bd menaquinol oxidase subunit I	2.94	2.54
BSU_40930	*yyaE*	respiration	putative oxidoreductase (molybdoenzyme)	2.81	2.34
BSU_13250	*ykoG*	signal transduction	two-component response regulator [YkoH]	2.00	2.06
BSU_26430	*yrkP*	signal transduction	two-component response regulator [YrkQ]	4.75	2.18
BSU_36350	*ywpD*	signal transduction	putative two-component sensor histidine kinase	7.02	2.38

BSU_11515	.	sporulation	conserved hypothetical protein	20.07	3.12
BSU_19430	*cdaS*	sporulation	spore diadenylate cyclase	9.29	8.82
BSU_06300	*cotA*	sporulation	outer spore coat copper-dependent promiscuous laccase	2.91	2.65
BSU_40530	*cotF*	sporulation	spore coat protein	2.82	2.10
BSU_30920	*cotI*	sporulation	spore coat kinase	2.31	2.38
BSU_06890	*cotJA*	sporulation	component of the inner spore coat	3.10	3.60
BSU_06900	*cotJB*	sporulation	component of the inner spore coat	3.98	3.61
BSU_06850	*cotK*	sporulation	spore inner coat protein	3.14	3.53
BSU_28100	*cotN*	sporulation	spore coat protein	6.55	3.35
BSU_34530	*cotR*	sporulation	spore coat lipolytic enzyme	3.08	2.27
BSU_00880	*disA*	sporulation	diadenylate cyclase; DNA integrity scanning protein; cell cycle checkpoint DNA scanning protein	5.80	2.15
BSU_32350	*fisB*	sporulation	protein required for membrane fission during spore formation	2.12	3.24
BSU_03930	*gdh*	sporulation	forespore glucose 1-dehydrogenase	10.72	6.54
BSU_35800	*gerBA*	sporulation	component of germinant receptor B	2.31	2.02
BSU_37920	*gerQ*	sporulation	inner spore coat protein	2.80	2.19
BSU_30980	*glgB*	sporulation	1,4-alpha-glucan branching enzyme	3.23	2.44
BSU_19700	*kamB*	sporulation	epsilon-amino-beta-lysine acetyl transferase	6.10	2.98
BSU_31460	*kapB*	sporulation	factor required for KinB signal transduction and activation of the phosphorelay to sporulation	6.00	5.90
BSU_01560	*kbaA*	sporulation	inner membrane protein involved in activation of the KinB signaling pathway to sporulation	3.37	2.23
BSU_28210	*lonB*	sporulation	spore-specific ATP-dependent protease LonB	4.52	2.34
BSU_05050	*lrpA*	sporulation	transcriptional regulator (Lrp/AsnC family)	3.62	2.62
BSU_24160	*mmgB*	sporulation	3-hydroxybutyryl-CoA dehydrogenase	5.36	5.26
BSU_24150	*mmgC*	sporulation	propionyl-CoA dehydrogenase subunit	6.38	6.46
BSU_25750	*nucB*	sporulation	sporulation-specific matrix degrading exported DNase	7.18	3.64
BSU_10110	*pbpF*	sporulation	penicillin-binding protein 2C (1F class) required for spore germination	3.02	3.46
BSU_27830	*sgpA*	sporulation	spore germination protein	6.82	3.12
BSU_22930	*sleB*	sporulation	spore germination cortex-lytic enzyme	3.70	2.67
BSU_10950	*slpS*	sporulation	2-phospho-3-sulfolactate synthase	3.83	2.06
BSU_09780	*spaC*	sporulation	spore coat associated protein, subunit C	3.65	2.14
BSU_23530	*spoIIM*	sporulation	autolysin component for dissolution of the septal cell wall (stage II sporulation)	2.04	3.06
BSU_36550	*spoIIQ*	sporulation	forespore protein required for alternative engulfment	17.12	6.72
BSU_24230	*spoIVB*	sporulation	regulatory membrane-associated serine protease	2.12	2.37
BSU_25770	*spoIVCA*	sporulation	site-specific DNA recombinase; skin element	6.31	3.76
BSU_05710	*spoL*	sporulation	spore cortex lytic enzyme	2.76	2.55
BSU_16730	*spoVFA*	sporulation	spore dipicolinate synthase subunit A	6.50	3.10
BSU_16740	*spoVFB*	sporulation	spore dipicolinate synthase subunit B	4.75	2.84
BSU_28110	*spoVID*	sporulation	morphogenetic spore protein (stage VI sporulation)	5.35	2.84
BSU_17420	*spoVK*	sporulation	mother cell sporulation ATPase	4.54	4.04

BSU_09400	*spoVR*	sporulation	involved in spore cortex synthesis (stage V sporulation, conserved in non sporulating bacteria	3.06	2.62
BSU_29570	*sspA*	sporulation	small acid-soluble spore protein (alpha-type SASP)	2.53	2.08
BSU_33340	*sspJ*	sporulation	small acid-soluble spore protein	2.87	2.52
BSU_misc_RNA_32	*ssrSA*	sporulation	6Sa RNA	2.54	2.06
BSU_34790	*trxB*	sporulation	thioredoxin reductase	5.82	4.31
BSU_00080	*yaaC*	sporulation	conserved protein of unknown function	11.42	6.31
BSU_01520	*ybaK*	sporulation	conserved protein of unknown function	12.09	3.94
BSU_03400	*yckD*	sporulation	putative exported protein	4.70	2.76
BSU_03690	*yczF*	sporulation	conserved protein of unknown function	4.60	2.24
BSU_05540	*ydfS*	sporulation	conserved hypothetical protein	11.09	5.57
BSU_07450	*yfmJ*	sporulation	putative oxidoreductase	4.13	4.48
BSU_08770	*ygzA*	sporulation	conserved protein of unknown function	3.33	2.88
BSU_08920	*yhbB*	sporulation	conserved hypothetical protein	4.24	2.75
BSU_09150	*yhcN*	sporulation	putative germination lipoprotein	5.74	3.34
BSU_09165	*yhcO*	sporulation	putative exported protein (sporulation germination island)	4.32	2.78
BSU_09180	*yhcQ*	sporulation	putative spore coat protein	5.89	3.08
BSU_10280	*yhfM*	sporulation	putative membrane protein	14.50	7.07
BSU_10400	*yhxC*	sporulation	putative oxidoreductase	3.07	2.48
BSU_11139	*yizC*	sporulation	conserved hypothetical protein; genus orphan	2.42	2.09
BSU_11549	*yizD*	sporulation	conserved hypothetical protein	95.39	30.03
BSU_13710	*ykvI*	sporulation	putative transporter	3.87	3.74
BSU_13820	*ykvT*	sporulation	cell wall hydrolase related to spore cortex-lytic enzymes	6.28	2.71
BSU_13830	*ykvU*	sporulation	spore membrane protein involved in germination	8.63	3.23
BSU_13050	*ykzH*	sporulation	conserved hypothetical protein	36.91	14.33
BSU_17720	*yndA*	sporulation	putative exported protein of unknown function	2.48	2.07
BSU_19610	*yodI*	sporulation	putative spore coat protein	2.19	2.22
BSU_19670	*yodN*	sporulation	conserved hypothetical protein	6.20	2.91
BSU_19710	*yodQ*	sporulation	putative deacetylase	5.87	4.05
BSU_19660	*yozD*	sporulation	conserved hypothetical protein	5.99	3.18
BSU_21970	*ypeP*	sporulation	ribonuclease	4.35	2.83
BSU_21960	*ypeQ*	sporulation	conserved protein of unknown function	3.21	2.44
BSU_25730	*yqeC*	sporulation	putative catabolic 6-phospho-gluconate dehydrogenase (NAD[+]-dependent)	9.85	4.87
BSU_23620	*yqkF*	sporulation	NADPH-dependent aldo-keto reductase (acts on 4-hydroxy-2,3-trans-nonenal)	3.31	4.24
BSU_27680	*yrbG*	sporulation	conserved hypothetical protein	4.08	2.26
BSU_27470	*yrrD*	sporulation	conserved protein of unknown function	2.63	3.67
BSU_27468	*yrzQ*	sporulation	conserved protein of unknown function	12.86	5.35
BSU_27469	*yrzR*	sporulation	conserved protein of unknown function	3.09	3.28
BSU_30330	*ytvB*	sporulation	putative conserved membrane protein	5.60	2.56
BSU_30470	*ytzC*	sporulation	conserved protein of unknown function	4.93	2.86
BSU_31300	*yugS*	sporulation	putative membrane protein	18.84	12.59
BSU_32360	*yunC*	sporulation	conserved protein of unknown function	2.03	2.90

BSU_31810	*yuzE*	sporulation	conserved protein of unknown function	4.69	6.35
BSU_31820	*yuzF*	sporulation	conserved protein of unknown function	16.93	2.55
BSU_33550	*yvaC*	sporulation	putative integral inner membrane protein	2.90	2.33
BSU_35040	*yvnB*	sporulation	putative exported hydrolase	2.47	2.47
BSU_36490	*ywoC*	sporulation	putative enzyme of isochorismatase family	3.12	3.73
BSU_36210	*ywqH*	sporulation	conserved protein of unknown function	2.69	2.06
BSU_40560	*yybP*	sporulation	membrane protein, may be involved in manganese detoxification	2.64	2.32
BSU_40280	*yycO*	sporulation	conserved exported protein	5.13	4.00
BSU_40270	*yycP*	sporulation	conserved hypothetical protein	3.35	3.26
BSU_40460	*yyzB*	sporulation	conserved protein of unknown function	3.02	2.50
BSU_12340	*uxuA*	sporulation, carbon metabolism	D-mannonate dehydratase	2.09	2.51
BSU_12330	*yjmD*	sporulation, carbon metabolism	putative oxidoreductase	5.16	8.85
BSU_39510	*snaB*	sulfur metabolism	sulfur-containing amino acid acetyltransferase	2.90	2.38
BSU_29290	*sndA*	sulfur metabolism	S-alkyl-N-acetyl-metabolite deacetylase	2.50	2.06
BSU_39470	*sndB*	sulfur metabolism	N-acetyl-sulfur-metabolite deacetylase	2.78	3.62
BSU_10910	*yisZ*	sulfur metabolism	adenylylsulfate kinase	4.21	3.25
BSU_10920	*yitA*	sulfur metabolism	putative sulfate adenylyltransferase	4.18	3.05
BSU_10930	*yitB*	sulfur metabolism	putative phospho-adenylylsulfate reductase	5.35	2.61
BSU_36180	*rttO*	toxin-antitoxin systems	putative ribonuclease antitoxin	3.50	2.04
BSU_04470	*dctP*	transport	C4-dicarboxylate transport protein	2.06	2.14
BSU_17300	*ebrA*	transport	small toxic metabolite efflux transporter subunit	8.27	3.45
BSU_17290	*ebrB*	transport	small toxic metabolite efflux transporter subunit	5.31	2.71
BSU_10520	*glcP*	transport	glucose/mannose:H+ symporter (recent HGT island)	6.01	2.32
BSU_02420	*glnT*	transport	glutamine transporter	2.25	2.17
BSU_02340	*gltP*	transport	proton/glutamate symport protein	4.64	2.55
BSU_11640	*kefQ*	transport	gated K+/H+ antiporter	8.53	8.52
BSU_36470	*pucI*	transport	allantoin permease	4.72	4.61
BSU_35940	*rbsA*	transport	ribose ABC transporter (ATP-binding protein)	10.12	2.95
BSU_35960	*rbsB*	transport	ribose ABC transporter (ribose-binding lipoprotein)	8.59	3.23
BSU_35950	*rbsC*	transport	ribose ABC transporter (permease)	11.56	4.08
BSU_35930	*rbsD*	transport	ribose ABC transporter (membrane protein)	7.88	2.21
BSU_08850	*ssuC*	transport	aliphatic sulfonate ABC transporter (permease)	5.34	3.73
BSU_03610	*tcyA*	transport	cystine ABC transporter (substrate-binding lipoprotein)	3.45	4.17
BSU_03600	*tcyB*	transport	cystine ABC transporter (permease)	4.12	5.02
BSU_03590	*tcyC*	transport	cystine ABC transporter (ATP-binding protein)	3.86	4.84
BSU_00570	*yabM*	transport	putative exporter	2.89	3.34
BSU_01890	*ybcL*	transport	putative efflux transporter; prophage 1 region	9.02	4.08
BSU_03840	*ycnB*	transport	putative efflux transporter	7.32	5.02
BSU_05140	*ydzE*	transport	putative permease	5.96	2.26
BSU_08210	*yfiB*	transport	putative xenobiotic ABC transporter subunit (ATP-binding protein)	7.72	6.30

BSU_08220	*yfiC*	transport	putative ABC transporter (ATP-binding protein)	5.27	5.97
BSU_07460	*yfmI*	transport	putative efflux transporter	4.70	2.80
BSU_08690	*ygaD*	transport	putative ABC transporter (ATP-binding protein)	4.32	3.96
BSU_09550	*yhdP*	transport	putative magnesium efflux pump	2.13	2.06
BSU_10080	*yhfA*	transport	putative transporter	3.19	2.74
BSU_10590	*yhjP*	transport	similar to ABC transporter (peptide binding protein)	2.06	2.46
BSU_10870	*yisU*	transport	similiar to arginine export protein	3.75	2.46
BSU_12400	*yjnA*	transport	putative permease	4.56	4.95
BSU_14030	*ykuC*	transport	similar to macrolide-efflux protein	2.99	2.28
BSU_25010	*yqgE*	transport	putative efflux transporter	3.64	3.82
BSU_24740	*yqxL*	transport	CorA-type divalent ion transporter	5.71	3.32
BSU_30160	*ytcQ*	transport	polygalacturonan and rhamnogalacturonan ABC transporter (solute-binding lipoprotein)	2.44	3.08
BSU_30610	*ytlC*	transport	putative ABC anion transporter component, ATP-binding	2.31	2.54
BSU_29280	*ytnM*	transport	putative transporter	4.92	3.39
BSU_32380	*yunE*	transport	putative transporter	2.81	3.34
BSU_31480	*yuxJ*	transport	putative exporter induced in acid stress	6.07	9.22
BSU_37750	*ywfA*	transport	putative efflux transporter	2.13	2.29
BSU_37040	*ywkB*	transport	putative metabolite transporter	5.19	3.15
BSU_36480	*ywoD*	transport	putative efflux transporter	4.39	5.66
BSU_39500	*yxeM*	transport	putative ABC transporter (binding lipoprotein)	2.62	2.23
BSU_39490	*yxeN*	transport	putative ABC transporter (permease)	2.16	2.47
BSU_39480	*yxeO*	transport	putative ABC transporter (ATP-binding protein)	2.17	2.19
BSU_39000	*yxjC*	transport	putative acid metabolite permease	2.03	2.79
BSU_28740	*araP*	transport, carbon metabolism	arabinose/arabinan permease	3.94	2.60
BSU_28730	*araQ*	transport, carbon metabolism	arabinose/arabinan permease	3.53	2.02
BSU_38050	*sacP*	transport, carbon metabolism	phosphotransferase system (PTS) sucrose-specific enzyme IIBC component	2.09	2.23
BSU_22230	*ypqE*	transport, carbon metabolism	putative phosphotransferase system enzyme IIA component	2.74	2.31
BSU_31540	*nupN*	transport, nucleotide metabolism	lipoprotein involved in guanosine transport	4.83	3.01
BSU_31550	*nupO*	transport, nucleotide metabolism	guanosine ABC transporter (ATP-binding protein)	4.97	4.63
BSU_15640	*fbnA*	tRNAs	putative tRNA modification protein	2.59	2.29
BSU_27500	*mnmA*	tRNAs	tRNA-specific 2-thiouridylase	2.16	2.46
BSU_00180	*tadA*	tRNAs	tRNA specific adenosine A34 deaminase	4.39	2.19
BSU_misc_RNA_58	*tboTA*	tRNAs	T-box riboswitch specific of threonine tRNA ligase	6.41	4.01
BSU_00670	*tilS*	tRNAs	tRNA(ile2) lysidine synthetase	3.84	2.08
BSU_29580	*trmG*	tRNAs	persulfide ATP pyrophosphatase involved in tRNA modification	2.79	2.17
BSU_tRNA_73	*trnSL-Gln1*	tRNAs	tRNA-Gln	6.41	3.79
BSU_04345	.	unknown	hypothetical protein	4.70	2.80
BSU_11525	.	unknown	conserved hypothetical protein	3.96	4.73
BSU_13545	.	unknown	hypothetical protein	2.24	3.32

BSU_17099	.	unknown	hypothetical protein	2.88	2.13
BSU_18595	.	unknown	hypothetical protein	3.17	2.06
BSU_24205	.	unknown	hypothetical protein	13.12	8.13
BSU_25565	.	unknown	hypothetical protein	7.37	2.87
BSU_26827	.	unknown	hypothetical protein	3.65	2.26
BSU_27185	.	unknown	hypothetical protein	3.99	2.56
BSU_27786	.	unknown	hypothetical protein	6.36	4.43
BSU_40358	.	unknown	hypothetical protein	2.97	2.38
BSU_36710	*fdhD*	unknown	protein sulfur transferase involved in molybdenum cofactor synthesis	4.27	4.72
BSU_misc_RNA_94	*mswM*	unknown	manganese ion binding riboswitch	3.20	2.54
BSU_14050	*ppeE*	unknown	exported metallophosphoesterase (Mn2+ and Zn2+)	4.07	2.75
BSU_26410	*psiE*	unknown	phosphate starvation inducible protein	3.16	2.09
BSU_03030	*ycgB*	unknown	putative integral inner membrane protein of unknown function	7.03	3.75
BSU_03160	*ycgJ*	unknown	similar to predicted S-adenosylmethionine-dependent methyltransferase	4.43	2.57
BSU_02710	*yczC*	unknown	putative integral membrane protein of unknown function	2.46	2.30
BSU_05260	*ydeN*	unknown	putative alpha/beta hydrolase	3.04	3.69
BSU_05290	*ydeP*	unknown	putative transcriptional regulator	8.51	2.57
BSU_05580	*ydgC*	unknown	putative transcriptional regulator	4.19	3.57
BSU_05590	*ydgD*	unknown	conserved hypothetical protein	3.90	3.26
BSU_06120	*ydjB*	unknown	hypothetical protein; prophage region 3	3.63	2.97
BSU_06130	*ydjC*	unknown	conserved hypothetical protein; prophage region 3	3.38	2.60
BSU_04359	*ydzK*	unknown	putative membrane protein	3.99	2.11
BSU_06048	*ydzU*	unknown	hypothetical protein; prophage 3 region	9.53	8.17
BSU_07240	*yetN*	unknown	conserved hypothetical protein	2.28	2.09
BSU_08490	*yfhD*	unknown	conserved hypothetical protein	4.17	3.87
BSU_08500	*yfhE*	unknown	hypothetical protein	4.05	3.07
BSU_08510	*yfhF*	unknown	putative nucleotide-or NAD-binding protein	3.48	3.93
BSU_08620	*yfhP*	unknown	putative membrane hydrolase	4.30	2.05
BSU_08680	*ygaC*	unknown	putative RNA binding factor	4.43	3.51
BSU_09250	*yhcX*	unknown	putative amidohydrolase	3.02	3.67
BSU_10090	*yhgB*	unknown	hypothetical protein	2.40	2.63
BSU_10570	*yhjN*	unknown	putative integral inner membrane protein (recent HGT island)	3.03	2.01
BSU_08900	*yhzB*	unknown	conserved hypothetical protein	7.03	5.12
BSU_12140	*yjgA*	unknown	conserved membrane protein	6.41	2.98
BSU_13030	*ykhA*	unknown	promiscuous acyl-CoA thioesterase	3.57	3.30
BSU_13819	*ykzS*	unknown	conserved hypothetical protein (HGT island)	3.41	3.06
BSU_15100	*ylbP*	unknown	putative acetyltransferase	5.58	2.58
BSU_17360	*ymzA*	unknown	conserved hypothetical protein	9.55	6.79
BSU_17699	*ynzK*	unknown	putative membrane protein of unknown function (phage origin)	4.37	5.61
BSU_18810	*yobA*	unknown	hypothetical protein	2.93	3.21
BSU_20760	*yopU*	unknown	hypothetical protein; phage SPbeta	2.83	2.55

BSU_20630	*yoqH*	unknown	conserved hypothetical protein; phage SPbeta	2.28	2.23
BSU_20530	*yoqS*	unknown	putative RNA-binding protein; phage SPbeta	3.87	3.98
BSU_20510	*yoqU*	unknown	conserved hypothetical protein; phage SPbeta	3.71	2.88
BSU_18860	*yozH*	unknown	hypothetical protein; putative defective prophage 6	4.45	2.21
BSU_18900	*yozJ*	unknown	hypothetical protein	5.87	4.44
BSU_18898	*yozW*	unknown	hypothetical protein; putative defective prophage 6	4.76	3.36
BSU_21880	*ypgR*	unknown	putative lyase or scaffold	2.83	2.67
BSU_22590	*ypiA*	unknown	conserved protein of unknown function	7.15	4.76
BSU_26210	*yqaR*	unknown	hypothetical protein; skin element	3.97	2.68
BSU_25950	*yqcC*	unknown	conserved phage protein of unknown function; skin element	3.75	2.34
BSU_24910	*yqgM*	unknown	putative glycosyltransferase	2.16	34.16
BSU_26550	*yrkD*	unknown	putative metal-sensitive transcriptional regulator involved in sulfur metabolism	6.22	2.25
BSU_26510	*yrkH*	unknown	putative sulfur transferase / hydrolase	9.43	2.73
BSU_26400	*yrkS*	unknown	hypothetical protein	3.36	2.69
BSU_26826	*yrpDX*	unknown	expressed polypeptide of unknown function	8.21	5.70
BSU_27490	*yrrB*	unknown	putative tetratricopeptide repeat family protein	2.06	2.73
BSU_28330	*ysnE*	unknown	putative indole acetic acid N-acetyltransferase	2.46	2.26
BSU_29550	*ytcJ*	unknown	putative N-substituted formamide deformylase, dihydropyrimidinase or isoaspartyl peptidase	3.75	5.52
BSU_30580	*ytmA*	unknown	putative esterase	2.48	2.25
BSU_29870	*ytzB*	unknown	conserved protein of unknown function	5.69	3.76
BSU_32370	*yunD*	unknown	putative nuclease/nucleotidase/phosphoesterase	2.10	2.88
BSU_32390	*yunF*	unknown	conserved protein of unknown function	2.91	3.26
BSU_32120	*yuzG*	unknown	conserved protein or unknown function	12.56	4.27
BSU_34730	*yvcN*	unknown	putative arylamine N-acetyltransferase	2.66	2.93
BSU_35140	*yvkN*	unknown	conserved protein of unknown function	7.65	2.67
BSU_37990	*ywdE*	unknown	putative integral inner membrane protein of unknown function	3.27	2.04
BSU_37140	*ywjG*	unknown	conserved protein of unknown function	5.64	3.50
BSU_36150	*ywqN*	unknown	putative oxidoreductase	2.80	2.06
BSU_36080	*ywrF*	unknown	putative flavin-binding monooxygenase	7.41	3.91
BSU_38018	*ywzG*	unknown	putative transcriptional regulator, PadR family	11.90	3.71
BSU_39800	*yxcD*	unknown	conserved protein of unknown function	5.08	2.24
BSU_39570	*yxeF*	unknown	lipocalin-like lipoprotein	2.12	2.19
BSU_39460	*yxeQ*	unknown	putative catabolic enzyme	2.05	3.97
BSU_39910	*yxnB*	unknown	conserved protein of unknown function	8.05	4.27
BSU_40790	*yyaO*	unknown	conserved hypothetical protein	8.69	2.34
BSU_40730	*yyaS*	unknown	putative integral inner membrane protein	3.15	2.40
BSU_40939	*yyzM*	unknown	putative nucleic acid binding protein	5.86	2.72

Table S2: Transcripts uniquely induced by ES24. IF: induction factor

locus tag	gene name	category	gene product	IF ES24
BSU_misc_RNA_46	*kswC*	amino acid metabolism	lysine riboswitch	2.05
BSU_misc_RNA_91	*pswI*	amino acid metabolism	proline T-box riboswitch	2.87
BSU_37770	*rocB*	amino acid metabolism	putative N-deacylase involved in arginine and ornithine utilization	2.17
BSU_22670	*trpD*	amino acid metabolism	anthranilate phosphoribosyltransferase	2.86
BSU_22650	*trpF*	amino acid metabolism	phosphoribosylanthranilate isomerase	2.19
BSU_21910	*metAA*	amino acid metabolism, cell wall synthesis	homoserine O-acetyltransferase	2.77
BSU_29590	*iscSB*	amino acid metabolism, sulfur metabolism	cysteine desulfurase	3.28
BSU_37680	*bacG*	antibiotic production	cyclohexenol-containing tetrahydro-4-hydroxyphenylpyruvate H(4)HPP in bacilysin synthesis	2.17
BSU_10550	*ntdA*	antibiotic production	biosynthesis of neotrehalosadiamine (3,3'-diamino-3,3'-dideoxy-alpha,beta-trehalose);3-oxo-glucose-6-phosphate:glutamate aminotransferase; kanosamine aminotransferase (recent HGT island)	3.65
BSU_10540	*ntdB*	antibiotic production	biosynthesis of neotrehalosadiamine (3,3'-diamino-3,3'-dideoxy-alpha,beta-trehalose);kanosamine-6-phosphate phosphatase (recent HGT island)	3.30
BSU_10530	*ntdC*	antibiotic production	biosynthesis of neotrehalosadiamine (3,3'-diamino-3,3'-dideoxy-alpha,beta-trehalose), glucose-6-P 3-dehydrogenase; kanosamine biosynthesis (recent HGT island)	2.67
BSU_10560	*ntdR*	antibiotic production	transcriptional regulator of the ntd operon (NtdR-NTD) (recent HGT island)	2.36
BSU_17140	*pksF*	antibiotic production	decarboxylase converting malonyl-S-AcpK to acetyl-S-AcpK for bacillaene-related polyketide synthesis	2.40
BSU_24020	*bmrR*	antibiotic resistance	transcriptional regulator (MerR family)	2.05
BSU_29040	*ytbD*	antibiotic resistance	putative transporter	10.25
BSU_31321	*mstX*	biofilms	atypical membrane-integrating regulator of biofilm formation (Mistic protein)	8.67
BSU_08060	*acoA*	carbon metabolism	acetoin dehydrogenase E1 component (TPP-dependent alpha subunit)	2.08
BSU_28790	*araB*	carbon metabolism	L-ribulokinase	2.37
BSU_28780	*araD*	carbon metabolism	L-ribulose-5-phosphate 4-epimerase	2.77
BSU_28770	*araL*	carbon metabolism	glycolytic and pentose phosphate intermediates phosphatase	2.52
BSU_28750	*araN*	carbon metabolism	sugar-binding lipoprotein	3.49
BSU_39260	*bglH*	carbon metabolism	aryl-phospho-beta-d-glucosidase	3.17
BSU_05860	*gmuE*	carbon metabolism	ROK fructokinase; glucomannan utilization protein E	2.55
BSU_05870	*gmuF*	carbon metabolism	phosphohexomutase; cupin family	2.52
BSU_05850	*gmuR*	carbon metabolism	transcriptional regulator (GntR family)	2.64
BSU_39740	*iolC*	carbon metabolism	2-deoxy-5-keto-D-gluconic acid kinase	2.12

BSU_39720	*iolE*	carbon metabolism	scyllo-inosose dehydratase	3.67
BSU_39700	*iolG*	carbon metabolism	myo-inositol 2-dehydrogenase/D-chiro-inositol 3-dehydrogenase	3.11
BSU_39690	*iolH*	carbon metabolism	putative sugar-phosphate epimerase/isomerase	2.80
BSU_39680	*iolI*	carbon metabolism	inosose isomerase	2.83
BSU_39670	*iolJ*	carbon metabolism	2-deoxy-5-keto-D-gluconic acid 6-phosphate aldolase	2.99
BSU_34460	*levB*	carbon metabolism	endolevanase, selectively cleaves the (beta-2,6) fructosyl bonds	2.49
BSU_31520	*maeL*	carbon metabolism	two-component sensor histidine kinase [MaeM]	2.59
BSU_31530	*maeM*	carbon metabolism	two-component response regulator for malate metabolism [MaeL]	2.56
BSU_12020	*manA*	carbon metabolism	mannose-6 phosphate isomerase; cupin family	4.66
BSU_34620	*mdxD*	carbon metabolism	maltogenic alpha-amylase	2.04
BSU_34630	*mdxR*	carbon metabolism	transcriptional activator of the maltodextrin operon (LacI family)	5.52
BSU_35920	*rbsK*	carbon metabolism	ribokinase	3.96
BSU_35910	*rbsR*	carbon metabolism	ribose operon repressor (LacI family, D-ribose)	3.35
BSU_31180	*rhaA*	carbon metabolism	L-rhamnose isomerase	4.15
BSU_31210	*rhaR*	carbon metabolism	transcriptional regulator of the rhamnose operon (RhaR / l-rhamnulose-1-phosphate)	2.05
BSU_36920	*rpiB*	carbon metabolism	D-ribose 5-phosphate epimerase (promiscuous)	4.34
BSU_38070	*sacT*	carbon metabolism	transcriptional antiterminator of sacAP expression	3.37
BSU_17610	*xylB*	carbon metabolism	xylulose kinase	2.12
BSU_34200	*sigL*	carbon, amino acid, and fatty acid metabolism	RNA polymerase sigma-54 factor (sigma-L)	2.16
BSU_00620	*divIC*	cell division	cell-division initiation protein	2.01
BSU_23616	*mciZ*	cell division	cell division inhibitor	3.97
BSU_28610	*zapA*	cell division	regulator of cell division	3.68
BSU_17640	*alrB*	cell wall synthesis and turnover	alanine racemase (minor activity)	6.13
BSU_04230	*amj*	cell wall synthesis and turnover	lipid II flippase	3.77
BSU_02370	*gamR*	cell wall synthesis and turnover	transcriptional regulator-GlcN6P (GntR family)	3.95
BSU_26810	*murI*	cell wall synthesis and turnover	glutamate racemase	2.81
BSU_38120	*rodA*	cell wall synthesis and turnover	glycosyltransferase involved in extension of the lateral walls of the cell	2.84
BSU_35600	*tuaB*	cell wall synthesis and turnover	putative exporter involved in biosynthesis of teichuronic acid	2.78
BSU_35590	*tuaC*	cell wall synthesis and turnover	putative glycosyltransferase (teichuronic acid)	2.63
BSU_35560	*tuaF*	cell wall synthesis and turnover	putative hydrolase involved in teichuronic acid synthesis	2.13
BSU_35550	*tuaG*	cell wall synthesis and turnover	putative glycosyltransferase (teichuronic acid)	2.31
BSU_08980	*yhbH*	cell wall synthesis and turnover	factor involved in shape determination	2.52
BSU_13789	*ykzQ*	cell wall synthesis and turnover	putative peptidoglycan binding protein	3.22
BSU_24530	*lipM*	co-factors	protein octanoyltransferase	2.23
BSU_27870	*nadB*	co-factors	L-aspartate oxidase	4.48
BSU_16670	*ribC*	co-factors	bifunctional riboflavin kinase FAD synthase	2.40
BSU_25590	*comEA*	competence	membrane bound high-affinity DNA-binding receptor	4.30

BSU_25570	*comEC*	competence	DNA channel for uptake in competent cells	3.99
BSU_25600	*comER*	competence	putative pyrroline-5'-carboxylate reductase	2.22
BSU_35470	*comFA*	competence	ATP-dependent helicase competence protein	5.03
BSU_24680	*comGF*	competence	component of the DNA transport pilin platform	2.74
BSU_24670	*comGG*	competence	component of the DNA transport pilin platform	2.52
BSU_03620	*bsdA*	detoxification	HTH-type transcriptional regulator BsdA (LysR family)	4.60
BSU_08230	*catD*	detoxification	catechol-2,3-dioxygenase membrane subunit	30.78
BSU_08240	*catE*	detoxification	catechol-2,3-dioxygenase subunit	32.78
BSU_33680	*catR*	detoxification	transcriptional regulator of catechol dioxygenase	2.26
BSU_07220	*flvL*	detoxification	transcriptional regulator (FlvL-flavonoids)	2.08
BSU_38370	*glxA*	detoxification	glyoxalase I; hemithioacetal-bacillithiol lactoyl-bacillithiol formation	2.73
BSU_13040	*hmpA*	detoxification	flavohemoglobin	2.81
BSU_13850	*pfeT*	detoxification	Fe^{2+} efflux pump, P_{1B4}-type ATPase, protects the cell against iron intoxication	12.32
BSU_00260	*yaaN*	detoxification	putative toxic compound resistance protein	2.69
BSU_03652	*yclD*	detoxification	putative FMN-binding enzyme subunit	2.07
BSU_29050	*ytbE*	detoxification	promiscuous aldo/keto reductase	8.24
BSU_40860	*yyaH*	detoxification	putative glyoxalase	2.37
BSU_01810	*adaA*	DNA repair	trigger enzyme	2.73
BSU_16110	*dprA*	DNA repair	DNA processing Smf single strand binding protein	2.26
BSU_13410	*ligV*	DNA repair	ATP-dependent DNA ligase subunit Ku; AP/deoxyribose 5'-phosphate (5'-dRP)-lyase	2.31
BSU_36170	*nfi*	DNA repair	endonuclease V	2.42
BSU_05640	*ydgG*	DNA repair	putative transcriptional regulator (MarR family)	3.59
BSU_06740	*yefB*	DNA repair	putative site-specific recombinase / invertase; HGT island	2.23
BSU_18880	*yobE*	DNA repair	putative SOS response associated phage protein; putative defective prophage 6	6.04
BSU_18950	*yozL*	DNA repair	conserved hypothetical protein of phage origin; putative defective prophage 6	3.42
BSU_23650	*yqkC*	DNA repair	conserved protein of unknown function	2.45
BSU_01889	*ybzH*	gene regulation	putative transcriptional regulator (ArsR family); prophage 1 region	4.89
BSU_05170	*ydeE*	gene regulation	putative transcriptional regulator (AraC/XylS family)	2.25
BSU_05240	*ydeL*	gene regulation	putative PLP-dependent transcriptional regulator	2.73
BSU_08250	*yfiF*	gene regulation	putative transcriptional regulator (AraC/XylS family; cupin family)	5.10
BSU_10510	*yhjH*	gene regulation	putative transcriptional regulator	2.42
BSU_10880	*yisV*	gene regulation	putative PLP-dependent transcriptional regulator	2.25
BSU_13760	*ykvN*	gene regulation	putative transcriptional regulator (HGT island)	5.97
BSU_29030	*ytcD*	gene regulation	putative transcriptional regulator (HxlR family)	2.56
BSU_30020	*ytzE*	gene regulation	putative transcriptional regulator (DeoR family)	2.57
BSU_32920	*yusT*	gene regulation	transcriptional regulator of unknown specificity (LysR family)	2.32
BSU_33030	*yuxN*	gene regulation	putative transcriptional regulator	2.11
BSU_33580	*yvaF*	gene regulation	putative transcriptional regulator	2.33
BSU_37550	*ywhA*	gene regulation	putative transcriptional regulator (MarR family)	2.27
BSU_36160	*ywqM*	gene regulation	putative transcriptional regulator (LysR family)	2.25
BSU_40800	*yyaN*	gene regulation	putative transcriptional regulator (MerR family)	2.21

BSU_40710	*yybA*	gene regulation	transcriptional regulator (MarR family, polyamine export and degradation)	3.23
BSU_04680	*rsbS*	general stress response	antagonist of RsbT	2.35
BSU_04720	*rsbW*	general stress response	switch protein/serine kinase and anti-sigma factor (inhibitory sigma-B binding protein)	2.72
BSU_04730	*sigB*	general stress response	RNA polymerase sigma-37 factor (sigma(B))	2.06
BSU_08580	*spdL*	general stress response	factor for peptide SdpC immunity	2.25
BSU_00170	*yaaI*	general stress response	putative amidase (isochorismatase family)	2.10
BSU_05150	*ydeC*	general stress response	putative transcriptional regulator (AraC/XylS family)	4.55
BSU_06400	*yebE*	general stress response	conserved hypothetical protein	2.46
BSU_06410	*yebG*	general stress response	conserved hypothetical protein	3.81
BSU_17250	*ymaE*	general stress response	putative hydrolase	2.27
BSU_17240	*ymzB*	general stress response	conserved protein of unknown function involved in ethanol resistance	2.65
BSU_19240	*yocK*	general stress response	putative general stress protein	2.22
BSU_33850	*yvbG*	general stress response	putative integral membrane protein	4.59
BSU_36680	*ywmF*	general stress response	putative integral membrane protein	2.93
BSU_33520	*csoR*	ion homeostasis	repressor of copper utilisation proteins-Cu(I)	4.24
BSU_09560	*cueR*	ion homeostasis	indirect copper efflux transcriptional regulator	2.19
BSU_26650	*czcD*	ion homeostasis	potassium/proton-divalent cation antiporter	11.07
BSU_36600	*mta*	ion homeostasis	transcriptional regulator regulating efflux transporters synthesis (Fur-independent bacillibactin export)	7.20
BSU_33420	*nhaK*	ion homeostasis	Na+/H+ antiporter	16.70
BSU_07530	*yfmB*	ion homeostasis	conserved hypothetical protein	2.29
BSU_19520	*yojA*	ion homeostasis	putative H+/anion permease	2.79
BSU_26640	*yrdP*	ion homeostasis	putative oxidoreductase	8.99
BSU_26830	*zinT*	ion homeostasis	lipoprotein buffering protein for Zn2+ transport	2.75
BSU_03360	*zinU*	ion homeostasis	zinc metallochaperone with NTPase activity	3.26
BSU_07150	*hmoA*	iron-sulfur clusters, heme	heme-degrading monooxygenase	3.81
BSU_00900	*ispD*	lipid metabolism	2-C-methyl-D-erythritol 4-phosphate cytidylyltransferase, nonmevalonate isoprenoid pathway	4.18
BSU_00910	*ispF*	lipid metabolism	2-C-methyl-D-erythritol-2,4-cyclodiphosphate synthase	2.92
BSU_32910	*yusS*	lipid metabolism	conserved enzyme of unknown function	2.22
BSU_09510	*asiMA*	membrane stress	negative regulator of the activity of sigma-M	2.74
BSU_40170	*liaE*	membrane stress	AdoMet radical amino acid epimerase modifying factor LiaD	2.10
BSU_22940	*prsW*	membrane stress	protease required for RsiW anti-sigma(W) degradation	2.14
BSU_09520	*sigM*	membrane stress	RNA polymerase ECF (extracytoplasmic function)-type sigma factor (sigma(M))	3.56
BSU_00250	*xpaC*	membrane stress	putative phosphatase	3.73
BSU_02330	*ybfQ*	membrane stress	putative enzyme with rhodanese domain	2.92
BSU_02380	*ybgB*	membrane stress	conserved protein of unknown function (B. subtilis-specific)	2.14
BSU_03240	*ycgQ*	membrane stress	conserved protein of unknown function	2.13

BSU_22980	*ypbG*	membrane stress	putative phosphoesterase	2.00
BSU_27180	*yrhH*	membrane stress	putative methyltransferase	9.88
BSU_31000	*yuaI*	membrane stress	putative N-acetyltransferase	2.10
BSU_13950	*mcpC*	motility/chemotaxis	methyl-accepting chemotaxis protein	2.39
BSU_31250	*tlpA*	motility/chemotaxis	methyl-accepting chemotaxis protein (membrane curvature dependent localisation)	2.15
BSU_31230	*tlpB*	motility/chemotaxis	methyl-accepting chemotaxis protein	2.85
BSU_33690	*yvaQ*	motility/chemotaxis	putative methyl-accepting transducer	2.69
BSU_35330	*fliS*	motility/chemotaxis	flagellar assembly protein FliS	2.11
BSU_35350	*yvyC*	motility/chemotaxis	putative flagellar protein of unknown function	2.11
BSU_35440	*yvyF*	motility/chemotaxis	putative transcriptional regulator of flagella formation	2.38
BSU_35430	*flgM*	motility/chemotaxis	anti-sigma factor repressor of sigma(D)-dependent transcription	2.74
BSU_11940	*yjcP*	motility/chemotaxis	conserved hypothetical protein: phage island	2.07
BSU_11950	*yjcQ*	motility/chemotaxis	conserved hypothetical protein; phage island	2.46
BSU_26200	*yqaS*	motility/chemotaxis	putative phage-related terminase small subunit; skin element	3.01
BSU_09380	*nsrR*	nitrosative stress	NO-dependent activator of the ResDE regulon (Fe-S NO binding site)	2.57
BSU_01750	*cdaA*	nucleotide metabolism	diadenylate cyclase	2.81
BSU_34780	*nahA*	nucleotide metabolism	RNA pyrophosphohydrolase, converts primary transcripts to 5'-monophosphate RNA	3.92
BSU_32510	*pucA*	nucleotide metabolism	xanthine dehydrogenase molybdopterin recruitment factor	5.81
BSU_misc_RNA_27	*pyaB*	nucleotide metabolism	pyrimidine biosynthesis operon attenuator	3.55
BSU_15510	*pyrAA*	nucleotide metabolism	pyrimidine-specific carbamoyl-phosphate synthetase (small subunit, glutaminase subunit)	7.96
BSU_15490	*pyrB*	nucleotide metabolism	aspartate carbamoyltransferase	5.83
BSU_15500	*pyrC*	nucleotide metabolism	dihydroorotase	6.88
BSU_15530	*pyrK*	nucleotide metabolism	dihydroorotate dehydrogenase (electron transfer subunit)	5.91
BSU_38480	*relQ*	nucleotide metabolism	(p)ppGpp synthetase	3.00
BSU_40090	*ahpC*	oxidative stress	alkyl hydroperoxide reductase (small subunit)	2.88
BSU_40100	*ahpF*	oxidative stress	alkyl hydroperoxide reductase (large subunit)	2.92
BSU_19540	*arxR*	oxidative stress	transcriptional repressor	2.06
BSU_30340	*blrA*	oxidative stress	blue light GTP-binding receptor	3.25
BSU_35180	*csbA*	oxidative stress	conserved membrane protein of unknown function	2.54
BSU_13170	*guaD*	oxidative stress	guanine deaminase	2.59
BSU_32990	*mrgA*	oxidative stress	metalloregulation DNA-binding stress protein	33.47
BSU_38110	*nfrAA*	oxidative stress	FMN-containing NADPH-linked nitro/flavin reductase	11.00
BSU_03860	*nfrAB*	oxidative stress	NADPH-FMN oxidoreductase (nitroreductase)	38.98
BSU_08730	*perR*	oxidative stress	transcriptional regulator (Fur family)	3.23
BSU_19330	*sodF*	oxidative stress	superoxide dismutase (Fe2+-dependent)	5.27
BSU_05790	*ydhK*	oxidative stress	hypothetical protein	3.92
BSU_11490	*yjbC*	oxidative stress	putative thiol oxidation management factor; putative acetyltransferase	3.07
BSU_23830	*yqjL*	oxidative stress	putative hydrolase	3.45
BSU_23820	*yqjM*	oxidative stress	NADPH-dependent flavin oxidoreductase (acting on cinnamaldehyde-related compounds)	23.24

BSU_32770	*yusE*	oxidative stress	putative thiol-disulfide oxidoreductase with thioredoxin domain	4.81
BSU_38930	*yxjJ*	oxidative stress	conserved protein of unknown function	2.33
BSU_35870	*edmS*	PGA	factor required extrachromosomal elements maintenance	2.85
BSU_35860	*pgdS*	PGA	gamma-DD-glutamyl hydrolase (PGA depolymerase)	3.06
BSU_09410	*phoA*	phosphate metabolism	alkaline phosphatase A	9.25
BSU_32150	*paiA*	polyamine metabolism	polyamine N-acetyltransferase	2.09
BSU_30700	*rpmEB*	protein synthesis	ribosomal protein L31	3.40
BSU_rRNA_28	*rrnG-5S*	protein synthesis	ribosomal RNA-5S	3.66
BSU_00360	*rsmI*	protein synthesis	16S rRNA 2'-O-ribose C1402 methyltransferase	2.21
BSU_00270	*yaaO*	protein synthesis	putative decarboxylase	2.20
BSU_05950	*ydiF*	protein synthesis	putative energy-sensing inhibitor of translation	2.11
BSU_21940	*degR*	proteolysis	activator of degradative enzymes (aprE, nprE, sacB) production or activity	4.32
BSU_38470	*eapD*	proteolysis	exported double-zinc aminopeptidase	2.60
BSU_38400	*epr*	proteolysis	extracellular serine protease	2.91
BSU_24830	*yqgT*	proteolysis	putative d,l-endopeptidase	2.43
BSU_00330	*dnaH*	replication	subunit of the DNA replication complex	2.01
BSU_27160	*cypB*	respiration	cytochrome P450 CYP102A3	2.86
BSU_12210	*cypE*	respiration	cytochrome P450 CYP109B1, monooxygenase	3.38
BSU_22540	*qcrC*	respiration	menaquinol:cytochrome c oxidoreductase (cytochrome cc subunit)	2.03
BSU_24290	*xseB*	signal transduction	exodeoxyribonuclease VII (small subunit)	2.06
BSU_02010	*ybdK*	signal transduction	two-component system sensor histidine kinase [YbdJ]	2.16
BSU_26420	*yrkQ*	signal transduction	two-component sensor histidine kinase [YrkP]	2.51
BSU_34070	*yvfT*	signal transduction	two-component sensor histidine kinase [YvfU]	2.64
BSU_34060	*yvfU*	signal transduction	two-component response regulator [YvfT]	2.30
BSU_27750	*bofC*	sporulation	bypass of forespore C, intercompartmental signaling factor	2.35
BSU_05130	*cdnL*	sporulation	transcriptional regulator for repair and outgrowth of heat damaged spores	2.22
BSU_19780	*cgeA*	sporulation	spore outermost layer component	2.61
BSU_19790	*cgeB*	sporulation	protein involved in maturation of the outermost layer of the spore	3.80
BSU_19760	*cgeD*	sporulation	putative glycosyltransferase involved in maturation of the outermost layer of the spore	2.04
BSU_19750	*cgeE*	sporulation	protein involved in maturation of the outermost layer of the spore	2.89
BSU_36060	*cotH*	sporulation	spore coat protein kinase	2.29
BSU_31730	*cotIC*	sporulation	inner spore coat protein	3.96
BSU_06910	*cotJC*	sporulation	enzyme component of the inner spore coat	2.29
BSU_32270	*cotNH*	sporulation	spore coat-associated protein	2.54
BSU_32950	*cotNW*	sporulation	spore lipoprotein	2.27
BSU_30910	*cotSA*	sporulation	spore coat protein	2.16
BSU_00240	*csfB*	sporulation	forespore-specific anti-sigma factor	3.00
BSU_01530	*cwlD*	sporulation	N-acetylmuramoyl-L-alanine amidase	10.83
BSU_23190	*dacB*	sporulation	D-alanyl-D-alanine carboxypeptidase (penicillin-binding protein 5*) (required for spore cortex)	2.65
BSU_33050	*gerAA*	sporulation	component of the GerA germination receptor	3.02

BSU_33060	*gerAB*	sporulation	component of the germination receptor GerA; putative transporter	2.52
BSU_28410	*gerE*	sporulation	transcriptional regulator required for the expression of late spore coat genes	2.10
BSU_10700	*gerPC*	sporulation	spore germination protein	2.16
BSU_30970	*glgC*	sporulation	glucose-1-phosphate adenylyltransferase (ADP-glucose pyrophosphorylase) subunit alpha	2.34
BSU_40630	*ictI*	sporulation	inner spore coat protein	2.37
BSU_19690	*kamA*	sporulation	lysine 2,3-aminomutase	3.04
BSU_22970	*mecB*	sporulation	adaptor to ClpC; regulator of competence and sporulation	3.13
BSU_37510	*pbpG*	sporulation	sporulation specific penicillin-binding protein 2D	2.13
BSU_17900	*pcfA*	sporulation	factor controlling DNA replication	2.31
BSU_01570	*pdaB*	sporulation	polysaccharide deacetylase involved in sporulation	2.22
BSU_25830	*rapE*	sporulation	response regulator aspartate phosphatase; skin element	2.51
BSU_27840	*safA*	sporulation	morphogenetic protein associated with SpoVID	3.64
BSU_01920	*skfB*	sporulation	synthesis of sporulation killing factor A	2.57
BSU_01935	*skfC*	sporulation	sporulation killing factor biosynthesis and export	2.46
BSU_01950	*skfE*	sporulation	sporulation killing factor biosynthesis and export; ABC transporter (binding protein)	2.49
BSU_01960	*skfF*	sporulation	sporulation killing factor biosynthesis and export; ABC transporter (permease)	2.54
BSU_01970	*skfG*	sporulation	sporulation killing factor biosynthesis and export	4.29
BSU_10940	*slpH*	sporulation	2-phospho,3-sulfolactate phosphatase (sporulation related)	4.32
BSU_09770	*spaD*	sporulation	spore coat associated protein	3.11
BSU_23170	*spmB*	sporulation	spore maturation protein	2.32
BSU_07280	*spmG*	sporulation	putative CDP-sugar-dehydratase/epimerase	2.33
BSU_07270	*spmH*	sporulation	glucose-1-phosphate cytidylyltransferase (sporulation)	2.01
BSU_37130	*spo0F*	sporulation	two-component response regulator of sporulation initiation	2.09
BSU_28060	*spoIIB*	sporulation	spatial and temporal regulator of the dissolution of septal peptidoglycan during engulfment (stage II sporulation)	2.87
BSU_12830	*spoIISA*	sporulation	three-component apoptosis factor (ABC toxin/antitoxin/antitoxin system)	2.06
BSU_12815	*spoIISC*	sporulation	three component toxin / antitoxin / antitoxin SpoIISABC, antitoxin C	2.53
BSU_27670	*spoVB*	sporulation	involved in spore cortex synthesis (stage V sporulation); translocase with flippase function for peptidoglycan synthesis	2.19
BSU_14250	*sppO*	sporulation	spore protein cse15	2.45
BSU_19320	*sqhC*	sporulation	squalene-hopene cyclase, sporulenol synthase (spore protection)	2.87
BSU_08550	*sspK*	sporulation	small acid-soluble spore protein	3.41
BSU_22290	*sspM*	sporulation	small acid-soluble spore protein	4.47
BSU_misc_RNA_82	*surF*	sporulation	small untranslated RNA expressed under sporulation conditions (SurF-AbrB)	3.05
BSU_31270	*tgl*	sporulation	protein-glutamine gamma-glutamyltransferase (transglutaminase)	2.89
BSU_03680	*yclG*	sporulation	putative uronase	3.11
BSU_05530	*ydfR*	sporulation	conserved membrane protein of unknown function	2.88
BSU_05730	*ydhF*	sporulation	putative phosphate-starvation lipoprotein	2.16
BSU_08800	*ygaK*	sporulation	putative FAD-dependent oxido-reductase	26.31

BSU_10000	*yhaH*	sporulation	putative membrane protein; acid tolerance protein	2.19
BSU_09350	*yhdB*	sporulation	conserved hypothetical protein	8.57
BSU_10009	*yhzF*	sporulation	membrane protein of unknown function	2.99
BSU_10780	*yisN*	sporulation	conserved hypothetical protein	2.64
BSU_12110	*yjfA*	sporulation	conserved hypothetical protein	3.57
BSU_13060	*ykjA*	sporulation	conserved membrane protein of unknown function	3.22
BSU_13380	*ykoS*	sporulation	putative integral membrane protein	3.06
BSU_13390	*ykoT*	sporulation	putative glycosyltransferase	2.34
BSU_13780	*ykvP*	sporulation	spore protein (HGT island)	7.77
BSU_13940	*ykwB*	sporulation	putative acetyltransferase / amidohydrolase	6.23
BSU_13509	*ykzP*	sporulation	conserved protein of unknown function	5.96
BSU_14800	*ylaJ*	sporulation	putative germination lipoprotein	4.72
BSU_14950	*ylbB*	sporulation	putative enzyme	3.03
BSU_14970	*ylbD*	sporulation	sporulation-related protein (coat)	2.07
BSU_17320	*ymaF*	sporulation	putative sporulation-related protein of unknown function	2.93
BSU_18290	*yngL*	sporulation	putative integral inner membrane protein	3.25
BSU_18620	*yoaI*	sporulation	putative 4-hydroxyphenylacetate-3-hydroxylase	3.10
BSU_18700	*yoaQ*	sporulation	conserved hypothetical protein	2.24
BSU_19020	*yobN*	sporulation	putative amine oxidase (flavoprotein)	3.15
BSU_19510	*yojB*	sporulation	conserved hypothetical protein	3.70
BSU_19749	*yoyG*	sporulation	putative toxin of a type I toxin family (sporulation operon)	2.60
BSU_19680	*yozE*	sporulation	conserved protein of unknown function	3.14
BSU_18710	*yozF*	sporulation	putative lipoprotein	2.34
BSU_18600	*yozQ*	sporulation	putative sporulation protein	2.20
BSU_21950	*ypzA*	sporulation	putative spore coat protein	2.26
BSU_23350	*ypzD*	sporulation	putative germination protein	4.85
BSU_22849	*ypzH*	sporulation	conserved protein of unknown function	2.57
BSU_26240	*yqaO*	sporulation	conserved phage protein of unknown function; skin element	2.76
BSU_25820	*yqcI*	sporulation	conserved protein of unknown function; skin element	3.09
BSU_25080	*yqfX*	sporulation	conserved protein of unknown function expressed in germinating spores	2.12
BSU_32760	*yusD*	sporulation	conserved protein of unknown function	4.61
BSU_34640	*yvdD*	sporulation	putative enzyme	2.07
BSU_05010	*rapI*	sporulation, biofilms	ICEBs1 mobile element: response regulator aspartate phosphatase	2.71
BSU_misc_RNA_65	*sncO*	sporulation, biofilms	ICEBs1 mobile element: conserved small untranslated RNA	2.24
BSU_04990	*yddJ*	sporulation, biofilms	ICEBs1 mobile element: putative lipoprotein of unknown function	2.76
BSU_04940	*conE*	sporulation, biofilms, transport	ICEBs1 mobile element: VirB4-like ATPase	2.14
BSU_12380	*uxaB*	sporulation, carbon metabolism	tagaturonate reductase (altronate oxidoreductase)	2.05
BSU_36540	*ywnJ*	sporulation, membrane stress	putative integral inner membrane protein	4.22
BSU_38499	*ywzH*	sporulation, membrane stress	conserved hypothetical membrane protein	2.12
BSU_10390	*yhfW*	sporulation, respiration	putative oxidoreductase with Rieske [2Fe-2S] center	2.62

BSU_36190	*rttN*	sporulation, toxin-antitoxin systems	putative ribonuclease toxin	2.42
BSU_29400	*ascR*	sulfur metabolism	transcriptional regulator of operon snaA-ytnM degrading cysteine-containing compounds (AscR-N-acetylcysteine)	2.24
BSU_29330	*cmoO*	sulfur metabolism	monooxygenase (S-alkyl substrates)	3.37
BSU_33440	*cysJ*	sulfur metabolism	assimilatory sulfite reductase (flavoprotein alpha-subunit)	2.02
BSU_10070	*sndC*	sulfur metabolism	N-acetyl amino acid acetylase, promiscuous activity	2.41
BSU_misc_RNA_74	*asrE*	toxin-antitoxin systems	small regulatory antitoxin RNA, toxin-antitoxin type I system (BsrE/AsrE)	2.01
BSU_misc_RNA_77	*asrG*	toxin-antitoxin systems	antitoxin small RNA	2.02
BSU_misc_RNA_92	*asrH*	toxin-antitoxin systems	antitoxin RNA	2.84
BSU_misc_RNA_81	*ratA*	toxin-antitoxin systems	antisense RNA controlling synthesis of TxpA, membrane associated toxin of a toxin antitoxin system; skin element	2.02
BSU_26050	*txpA*	toxin-antitoxin systems	toxic peptide of toxin-antitoxin system; skin element	2.31
BSU_11390	*appB*	transport	oligopeptide ABC transporter (permease)	2.21
BSU_12310	*exuM*	transport	putative Na+:altronate/mannonate symporter	9.48
BSU_12360	*exuT*	transport	hexuronate transporter	2.13
BSU_32590	*frlN*	transport	fructose-amino acid permease	2.97
BSU_32600	*frlO*	transport	fructose amino acid-binding lipoprotein	2.28
BSU_03060	*lctP*	transport	L-lactate permease	2.08
BSU_36940	*mntP*	transport	manganese efflux pump	2.20
BSU_28910	*pftA*	transport	pyruvate uptake system subunit A	2.68
BSU_28900	*pftB*	transport	pyruvate import system subunit B	3.63
BSU_24970	*pstA*	transport	phosphate ABC transporter (permease)	4.54
BSU_24960	*pstBA*	transport	phosphate ABC transporter (ATP-binding protein)	3.26
BSU_24950	*pstBB*	transport	phosphate ABC transporter (ATP-binding protein)	3.91
BSU_24990	*pstS*	transport	phosphate ABC transporter (phosphate binding lipoprotein)	2.73
BSU_08840	*ssuA*	transport	aliphatic sulfonate ABC transporter (binding lipoprotein)	10.34
BSU_29370	*tcyK*	transport	sulfur-containing amino acid ABC transporter binding lipoprotein	11.66
BSU_05610	*vmlR*	transport	ATP-binding cassette efflux transporter	2.66
BSU_03250	*ycgR*	transport	putative permease	2.34
BSU_05650	*ydgH*	transport	putative membrane component	4.44
BSU_09110	*yhcJ*	transport	putative ABC transporter (binding lipoprotein)	2.71
BSU_10450	*yhjB*	transport	putative Na+/metabolite cotransporter	2.80
BSU_14320	*yknU*	transport	putative ABC transporter (ATP-binding protein)	3.63
BSU_14330	*yknV*	transport	putative ABC transporter (ATP-binding protein)	2.37
BSU_26860	*yraO*	transport	putative citrate transporter	2.62
BSU_26490	*yrkJ*	transport	putative permease	7.41
BSU_27690	*yrzE*	transport	putative transporter	2.34
BSU_30350	*yttB*	transport	putative efflux transporter	3.29
BSU_33570	*yvaE*	transport	similar to multidrug-efflux transporter	2.31
BSU_34090	*yvfR*	transport	putative ABC efflux transporter (ATP-binding protein)	3.81

BSU_34080	*yvfS*	transport	putative ABC transporter (permease)	4.60
BSU_33560	*yvaD*	transport, antibiotic resistance	similar to multidrug-efflux transporter	2.18
BSU_39710	*iolF*	transport, carbon metabolism	inositol transport protein	2.25
BSU_00340	*trmNF*	tRNAs	tRNA1(Val) (adenine(37)-N6)-methyltransferase	2.03
BSU_tRNA_7	*trnSL-Ser1*	tRNAs	tRNA-Ser	2.63
BSU_36950	*tsaC*	tRNAs	tRNA(NNU) t(6)A37 threonylcarbamoyladenosine modification; threonine-dependent ADP-forming ATPase	2.03
BSU_04745	.	unknown	hypothetical protein	5.18
BSU_18596	.	unknown	conserved hypothetical protein	2.46
BSU_21925	.	unknown	hypothetical protein	2.09
BSU_26399	.	unknown	hypothetical protein	5.57
BSU_27085	.	unknown	hypothetical protein	2.86
BSU_29845	.	unknown	hypothetical protein	2.36
BSU_31725	.	unknown	hypothetical protein	9.27
BSU_35678	.	unknown	hypothetical protein	2.48
BSU_36739	.	unknown	hypothetical protein	3.04
BSU_37569	.	unknown	hypothetical protein	2.07
BSU_38495	.	unknown	hypothetical protein	4.47
BSU_27170	*bscR*	unknown	transcriptional regulator for cypB	4.24
BSU_misc_RNA_64	*bsrC*	unknown	small regulatory RNA	3.35
BSU_misc_RNA_66	*sncZ*	unknown	putative small conserved untranslated RNA	5.90
BSU_02050	*ybdO*	unknown	putative phage protein; prophage region 1	3.63
BSU_03260	*ycgS*	unknown	putative aromatic hydrocarbon hydrolase	6.24
BSU_04790	*ydcK*	unknown	conserved protein of unknown function	2.04
BSU_05380	*ydfE*	unknown	putative flavoprotein	2.60
BSU_05450	*ydfK*	unknown	putative integral inner membrane protein	2.77
BSU_06820	*yeeG*	unknown	putative phage receptor protein	30.59
BSU_06870	*yesE*	unknown	conserved hypothetical protein	2.19
BSU_07480	*yfmG*	unknown	putative enzyme	2.45
BSU_10180	*yhfC*	unknown	putative integral membrane protein	2.02
BSU_10230	*yhfH*	unknown	conserved protein of unknown function	3.15
BSU_10410	*yhzC*	unknown	conserved protein of unknown function	2.51
BSU_09889	*yhzD*	unknown	conserved hypothetical protein	4.85
BSU_11070	*yitP*	unknown	conserved hypothetical protein	5.30
BSU_11960	*yjcR*	unknown	putative phage-related nucleic acid binding terminase small subunit; phage island	2.30
BSU_12030	*yjdF*	unknown	conserved hypothetical protein	2.87
BSU_13280	*ykoJ*	unknown	putative exported protein	2.06
BSU_13810	*ykvS*	unknown	conserved protein of unknown function (HGT island)	2.66
BSU_13320	*ykzB*	unknown	conserved protein of unknown function	2.68
BSU_15040	*ylbK*	unknown	putative hydrolase	2.53
BSU_18680	*yoaO*	unknown	conserved protein of unknown function	2.23
BSU_18690	*yoaP*	unknown	conserved hypothetical protein	2.50
BSU_18890	*yobF*	unknown	conserved hypothetical protein	3.39

BSU_19010	*yobM*	unknown	putative phage protein; putative defective prophage 6	2.26
BSU_19090	*yobU*	unknown	putative effector of transcriptional regulator	3.42
BSU_19100	*yobV*	unknown	putative transcriptional regulator	2.15
BSU_18400	*yoeD*	unknown	putative excisionase	2.34
BSU_21370	*yomG*	unknown	putative DNA wielding protein; SPbeta phage	2.83
BSU_21360	*yomH*	unknown	conserved protein of unknown function; phage SPbeta	5.44
BSU_21330	*yomK*	unknown	conserved protein of unknown function; phage SPbeta	2.03
BSU_20750	*yopV*	unknown	hypothetical protein; phage SPbeta	3.18
BSU_20540	*yoqR*	unknown	hypothetical protein; phage SPbeta	3.80
BSU_20520	*yoqT*	unknown	conserved hypothetical protein; phage SPbeta	5.33
BSU_20410	*yorE*	unknown	conserved protein of unknown function; phage SPbeta	2.90
BSU_20400	*yorF*	unknown	conserved hypothetical protein; phage SPbeta	2.45
BSU_21310	*yozP*	unknown	hypothetical protein; phage SPbeta	2.49
BSU_21760	*ypmP*	unknown	conserved hypothetical protein	2.23
BSU_26190	*yqaT*	unknown	putative phage-related terminase large subunit; skin element	2.35
BSU_26180	*yqbA*	unknown	putative phage capsid protein; skin element	2.00
BSU_26120	*yqbG*	unknown	conserved phage protein of unknown function; skin element	3.14
BSU_26075	*yqbK*	unknown	putative phage tail sheath protein; skin element	2.14
BSU_26010	*yqbQ*	unknown	conserved phage protein of unknown function; skin element	2.28
BSU_25990	*yqbS*	unknown	conserved phage protein of unknown function; skin element	5.48
BSU_25980	*yqbT*	unknown	putative phage baseplate assembly protein; skin element	8.69
BSU_25110	*yqfU*	unknown	putative integral inner membrane protein	2.60
BSU_24920	*yqgL*	unknown	conserved protein of unknown function	2.34
BSU_24820	*yqgU*	unknown	putative lipoprotein	2.16
BSU_24210	*yqiG*	unknown	putative NADH-dependent flavin oxidoreductase	17.11
BSU_23900	*yqjF*	unknown	conserved protein of unknown function	2.34
BSU_23640	*yqkD*	unknown	putative hydrolase	2.11
BSU_25920	*yqxG*	unknown	putative phage-related lytic exoenzyme; skin element	2.45
BSU_23519	*yqzK*	unknown	conserved protein of unknown function	2.99
BSU_26940	*yraH*	unknown	putative lyase	2.61
BSU_27800	*yrzH*	unknown	hypothetical protein	8.59
BSU_27190	*yrzI*	unknown	conserved protein of unknown function	3.09
BSU_28600	*yshB*	unknown	putative integral membrane protein	2.90
BSU_31780	*yueH*	unknown	conserved hypothetical protein	2.13
BSU_31340	*yugM*	unknown	putative transporter	2.09
BSU_31330	*yugN*	unknown	conserved protein of unknown function	2.69
BSU_32060	*yuiD*	unknown	putative integral inner membrane protein	2.08
BSU_32780	*yusF*	unknown	putative ribonuclease	3.83
BSU_32890	*yusQ*	unknown	hydratase of unidentified specificity (tautomerase superfamily)	2.93
BSU_32190	*yuzB*	unknown	conserved protein of unknown function	2.05
BSU_34840	*yvcB*	unknown	conserved protein of unknown function	2.10

BSU_35390	*yviE*	unknown	conserved protein of unknown function	3.83
BSU_35190	*yvkC*	unknown	putative phosphotransferase	2.18
BSU_34830	*yvzA*	unknown	conserved protein of unknown function	2.29
BSU_34729	*yvzJ*	unknown	putative lipoprotein	3.58
BSU_38000	*ywdD*	unknown	putative integral inner membrane protein of unknown function	3.12
BSU_37450	*ywhK*	unknown	factor interacting with DNA helicase PcrA	2.27
BSU_37440	*ywhL*	unknown	conserved protein of unknown function	2.56
BSU_36630	*ywnA*	unknown	putative transcriptional regulator	4.53
BSU_36620	*ywnB*	unknown	putative oxidoreductase	9.58
BSU_36220	*ywqG*	unknown	conserved protein of unknown function	2.03
BSU_36140	*ywqO*	unknown	conserved protein of unknown function	2.57
BSU_39790	*yxcE*	unknown	conserved protein of unknown function	3.93
BSU_39250	*yxiE*	unknown	phosphate starvation protein (universal stress protein A family)	4.35
BSU_40720	*yyaT*	unknown	putative acetyltransferase (polyamine degradation)	3.11
BSU_40640	*yybH*	unknown	conserved hypothetical protein	2.77

Table S3: Transcripts uniquely induced by NFT. IF: induction factor

locus tag	gene name	category	gene product	IF NFT
BSU_27250	*mccB*	amino acid metabolism	cystathionine gamma-lyase and homocysteine gamma-lyase for reverse transsulfuration pathway	2.48
BSU_18480	*proH*	amino acid metabolism	pyrroline-5-carboxylate reductase	2.07
BSU_18470	*proJ*	amino acid metabolism	glutamate 5-kinase	2.41
BSU_10240	*yhfI*	antibiotic resistance	putative metal-dependent hydrolase, metallo-beta-lactamase superfamily	2.82
BSU_31840	*bznD*	biofilms, competence, motility	benzil reductase (benzoin forming)	2.66
BSU_02490	*gudD*	carbon metabolism	glucarate dehydratase	3.01
BSU_22480	*mgsA*	carbon metabolism	methylglyoxal synthase	2.09
BSU_07080	*rhgZ*	carbon metabolism	beta-galacturonidase	2.15
BSU_38040	*sacA*	carbon metabolism	sucrose-6-phosphate hydrolase	2.68
BSU_23480	*dacF*	cell wall synthesis and turnover	D-alanyl-D-alanine carboxypeptidase (penicilin binding protein)	3.20
BSU_12990	*eepC*	cell wall synthesis and turnover	gamma-D-glutamyl-L-diaminoacid endopeptidase	2.21
BSU_misc_RNA_36	*fswA*	co-factors	flavin riboswitch	3.49
BSU_32800	*gcvH*	co-factors	glycine cleavage system protein H (lipoyl acceptor protein)	2.62
BSU_10250	*lplJ*	co-factors	lipoate-protein ligase	2.94
BSU_14310	*moaD*	co-factors	molybdopterin synthase (small subunit)	2.03
BSU_23260	*ribBA*	co-factors	fused 3,4-dihydroxy-2-butanone 4-phosphate synthase and GTP cyclohydrolase II	2.27
BSU_23280	*ribD*	co-factors	fused diaminohydroxyphosphoribosylaminopyrimidine deaminase; 5-amino-6-(5-phosphoribosylamino) uracil reductase	2.26
BSU_23270	*ribE*	co-factors	riboflavin synthase (alpha subunit)	2.35
BSU_23250	*ribH*	co-factors	riboflavin synthase (beta subunit)	2.48
BSU_23240	*ypzK*	co-factors	putative riboflavin synthesis-related N-acetyltransferase	2.29
BSU_02970	*yceK*	detoxification	putative transcriptional regulator (ArsR family)	10.41
BSU_10630	*addA*	DNA repair	ATP-dependent deoxyribonuclease (subunit A)	2.66
BSU_10620	*addB*	DNA repair	ATP-dependent deoxyribonuclease (subunit B)	2.19
BSU_10660	*hlpB*	DNA repair	HNH nuclease-like essential for DNA repair	2.48
BSU_17850	*lexA*	DNA repair	transcriptional repressor of the SOS regulon	2.88
BSU_27480	*recDB*	DNA repair	5'-3' helicase associated to SSB; exonuclease V	2.76
BSU_10650	*sbcC*	DNA repair	DNA ATP-dependent repair enzyme	2.82
BSU_10640	*sbcD*	DNA repair	DNA repair exonuclease	2.65
BSU_06840	*yeeI*	gene regulation	putative DNA integrase or transcriptional regulator	2.20
BSU_04400	*gsiB*	general stress response	general stress protein glucose starvation induced	2.02
BSU_04190	*ydaD*	general stress response	putative dehydrogenase	2.19

BSU_13169	*ykzN*	general stress response	hypothetical protein	2.54
BSU_25460	*dnaJ*	heat shock	co-factor of molecular chaperone	2.12
BSU_25470	*dnaK*	heat shock	molecular chaperone, ATP-dependent	2.10
BSU_06030	*groEL*	heat shock	chaperonin large subunit	3.61
BSU_00590	*hslR*	heat shock	ribosomal RNA binding protein involved in 50S recycling; heat shock protein	2.11
BSU_10100	*hmoB*	iron sulfur clusters, heme	heme-degrading monooxygenase	2.09
BSU_34390	*pnbA*	lipid metabolism	para-nitrobenzyl esterase (intracellular esterase B)	2.33
BSU_22420	*panC*	lipid metabolism, co-factors	pantothenate synthetase	2.01
BSU_22410	*panD*	lipid metabolism, co-factors	aspartate 1-decarboxylase	2.00
BSU_25070	*ispG*	lipid metabolism, sporulation	4-hydroxy-3-methylbut-2-en-1-yl diphosphate synthase (1-hydroxy-2-methyl-2-(E)-butenyl 4-diphosphate synthase)	2.04
BSU_00580	*yabN*	membrane stress	putative fusion methylase and nucleotide pyrophosphohydrolase	2.02
BSU_32480	*pucD*	nucleotide metabolism	xanthine dehydrogenase, substrate and molybdenum cofactor subunit	2.16
BSU_22460	*bshA*	oxidative stress	N-acetyl-alpha-D-glucosaminyl L-malate synthase; malate glycosyltransferase for bacillithiol synthesis	2.43
BSU_22470	*bshBA*	oxidative stress	N-acetyl-alpha-D-glucosaminyl L-malate deacetylase 1; malate N-acetylglucosamine N-acetyl hydrolase	2.40
BSU_22490	*dapB*	oxidative stress	(4S)-4-hydroxy-2,3,4, 5-tetrahydro-(2S)-dipicolinic acid (HTPA) dehydratase reductase	2.26
BSU_25020	*sodA*	oxidative stress	superoxide dismutase (Mn[2+]-dependent)	2.03
BSU_12410	*yjoA*	oxidative stress	bacillithiol S-transferase	3.50
BSU_19390	*yojN*	oxidative stress	putative nitric-oxide reductase associated protein	2.23
BSU_19380	*yojO*	oxidative stress	putative activator of nitric oxide reductase	2.52
BSU_29480	*ytxK*	oxidative stress	putative nucleic acid methyltransferase	2.08
BSU_07830	*hypO*	oxidative stress, respiration	NAD(P)H-flavin oxidoreductase (nitroreductase)	2.94
BSU_19800	*phyC*	phosphate metabolism	phytase	2.28
BSU_16870	*efpI*	protein synthesis	EF-P-5 aminopentanone reductase (EF-P repair enzyme), NADPH-dependent	3.79
BSU_21850	*rsmJ*	protein synthesis	putative 16S rRNA m(2)G1516 methyltransferase	2.06
BSU_16845	*ymfF*	proteolysis	putative metalloprotease	2.99
BSU_22080	*ypwA*	proteolysis	metal-dependent carboxypeptidase	2.28
BSU_13260	*ykoH*	signal transduction	two-component sensor histidine kinase [YkoG]	2.28
BSU_17670	*cotU*	sporulation	spore coat protein	4.70
BSU_25540	*gpr*	sporulation	spore germination protease	2.01
BSU_00160	*sleL*	sporulation	spore peptidoglycan N-acetylglucosaminidase	2.29
BSU_29070	*spcF*	sporulation	membrane calmodulin-like protein essential for stage III sporulation	2.09
BSU_00600	*spcP*	sporulation	spore protein involved in the shaping of the spore coat	2.21
BSU_25530	*spoIIP*	sporulation	spore autolysin (stage II sporulation)	2.23
BSU_40950	*sprC*	sporulation	spore-specific protease	2.35
BSU_07310	*yfnD*	sporulation	putative glycosyltransferase (complex carbohydrate synthase)	2.04
BSU_10610	*yhjR*	sporulation	putative electron carrier protein (putative sporulation gene)	4.31

BSU_16880	*ymfJ*	sporulation	putative enzyme	3.54
BSU_17760	*yndE*	sporulation	putative spore germination integral inner membrane protein	2.29
BSU_18280	*yngK*	sporulation	putative exported glycoside hydrolase	2.43
BSU_24580	*yqhH*	sporulation	putative RNA polymerase-associated helicase protein	2.44
BSU_27570	*yrzK*	sporulation	conserved protein of unknown function	2.20
BSU_14569	*ykzV*	toxin-antitoxin systems	putative type I toxin (toxin I motif)	4.32
BSU_39060	*citH*	transport	secondary transporter of divalent metal ions/citrate complexes	2.64
BSU_34590	*mdxG*	transport	maltodextrin ABC transporter (permease)	2.13
BSU_30110	*rmgS*	transport	lipoprotein required for rhamnogalaturonan transport and degradation	3.11
BSU_15650	*tcaB*	transport	P-type calcium transport ATPase (sporulation)	2.99
BSU_02960	*yceJ*	transport	putative carbohydrate transporter (possibly exporter)	116.38
BSU_18540	*yoaB*	transport	negatively charged metabolite transporter	2.02
BSU_36450	*ywoG*	transport	putative efflux transporter	2.36
BSU_39450	*yxeR*	transport	putative ethanolamine permease	2.19
BSU_22450	*cca*	tRNAs	tRNA nucleotidyltransferase	2.37
BSU_misc_RNA_22	*qswA*	tRNAs	preQ1 riboswitch	2.22
BSU_13730	*queD*	tRNAs	6-carboxy-5,6,7,8-tetrahydropterin synthase; queuosine biosynthesis	2.05
BSU_13740	*queE*	tRNAs	7-carboxy-7-deazaguanine synthase	2.01
BSU_22205	.	unknown	hypothetical protein	3.33
BSU_26569	.	unknown	hypothetical protein	2.06
BSU_29479	.	unknown	hypothetical protein	2.87
BSU_34399	.	unknown	hypothetical protein	4.31
BSU_30090	*rmgU*	unknown	putative membrane enzyme for rhamnogalaturonan degradation	2.48
BSU_02790	*ycdB*	unknown	conserved protein of unknown function	2.24
BSU_05400	*ydfG*	unknown	conserved hypothetical protein	2.48
BSU_07410	*yfmN*	unknown	hypothetical protein	2.69
BSU_10320	*yhfP*	unknown	putative oxidoreductase	2.44
BSU_14550	*ykrA*	unknown	putative hydrolase	2.11
BSU_14650	*yktB*	unknown	conserved protein of unknown function	2.73
BSU_20980	*yonV*	unknown	conserved protein of unknown function; phage SPbeta	2.02
BSU_20840	*yopM*	unknown	hypothetical protein; phage SPbeta	4.73
BSU_19970	*yosX*	unknown	conserved hypothetical protein; phage SPbeta	6.23
BSU_27729	*yrzS*	unknown	conserved membrane protein of unknown function	2.31
BSU_32790	*yusG*	unknown	conserved protein of unknown function	2.41

Table S4: Strains and plasmids used in this study.

species	strain	genotype	induction	reference
B. subtilis	168	*trpC2*	-	(2)
B. subtilis	1048	*cat rpoC-gfp Pxyl-'rpoC*	1% xylose	(3)
B. subtilis	1049	*amyE::spc Pxyl-rpsB-gfp*	1% xylose	(3)
B. subtilis	BS23	*atpA-gfp Pxyl-'atpA cat*	0.1% xylose	(4)
B. subtilis	BSN101	*secA::Pxyl-secA-gfp cat*	0.1% xylose	this study
B. subtilis	BWB09	*ΔxynA ΔamyE*	-	this study
B. subtilis	HM771	*dnaN::gfp-dnaN cat cat* (MS104 into 168)	-	(5)
B. subtilis	HS63	*amyE::spc Pxyl-divIVA-msfgfp*	0.5% xylose	(6)
B. subtilis	LH131	*amyE::spc Pxyl-gfp-minD*	0.1% xylose	(7)
B. subtilis	TEB1030	*trpC2 his nprE aprE bpf ispI lipA lipB*	-	(8)
B. subtilis	UG10	*amyE::spc Pxyl-recA-mgfp*	0.5% xylose	(9)
S. pneumoniae	D39	-	-	(10)
E. coli	BW25113	F-, Δ(araD-araB)567, ΔlacZ4787(::rrnB-3), rph-1, Δ(rhaD-rhaB)568, hsdR514	-	(11)
E. coli	KC606	BW25113 *ftsZ::ftsZ55-56-sfGFP*	-	(12)
E. coli	MC4100	*F− (araD139) Δ(argF-lac)169 λ− e14− flhD5301 Δ(fruK-yeiR)725(fruA25) relA1 rpsL150(Strr) rbsR22 Δ(fimB-fimE)632(::IS1) deoC1, spoT1]*	-	(13)
E. coli	RC1	*Δlac Δara ΔminCDE*		(14)

plasmid	genotype	induction	reference
pABCON2-*fhuA* ΔC/Δ4L	*P_{OXB}11-fhuA ΔC/Δ4L*	-	(15)
pBSlipA	*PhpaII-lipA*	-	(16)
pCS73	*neo bleo PamyQ-amyM*	-	(17)
pSH54	*Pxyl-secA-gfp cat*	-	this study
pFX9	*bla Plac-gfp-minD minE*	100 µM IPTG	(18)

Table S5: Primers used in this study.

Name	Sequence (5'-3')	Target
SV77	ATGCTTGGTACCTTAAATAAAATGTTTGATCCAAC	SecA-Fw
SV78	ATATGTCGACTTCAGTACGGCCGCAGC	SecA-Rv
BW05	CTAATTGAGAGAAGTTTCTATAGAATTTT	SpR-mazF-Fw
BW06	CTACCCAATCAGTACGTTAATTT	SpR-mazF-Rv
BW41	CAGATCATCCTTAATCAGGGGTAGCTAACG	XnyAUp-Fw
BW42	GAAACTTCTCTCAATTAGATTTCATGTAAACCGAGAACCA	XnyAUp-Rv
BW45	GCAAAAGCCCTTATGAGGGCTTTTTTAATTGTTGTTTGCAGTAAC	XnyADn-Fw
BW46	ACCCCTGATTAAGGATGATCTGATGTTACCTCCTATAATATTTTTTCCG	XnyADn-Rv
BW47	AATTAACGTACTGATTGGGTAGTTCTTAGTTGGATTATCGGCAGC	XnyA-Fw
BW48	GGATGATCTGTTACCACACTGTTACGTTAGAACTTCCACTAC	XnyA-Rv
BW49	ATGATCAATTGGGGGCCGTTTTAACGATTGCTGCC	AmyEUp-Fw
BW50	TCCCGTCTAGCCTTGCCCTCTTGACACTCCTTATTTGA	AmyEUp-Rv
BW51	GGGCAAGGCTAGACGGGACTTACCGAAAGAAA	AmyEDn-Fw
BW52	TATAGAAACTTCTCTCAATTAGCCCGCTCTTTTGGCAGGCCGC	AmyEDn-Rv
BW53	AACGTACTGATTGGGTAGGCCATTCAGACATCTCCGA	AmyE-Fw
BW54	CAGACCTGGCATTGATCGTGCCTGTCAGTTTAC	AmyE-Rv

Supplementary References

1. Strahl H, Hamoen LW. 2010. Membrane potential is important for bacterial cell division. Proc Natl Acad Sci U S A 107:12281–12286.

2. Anagnostopoulos C, Spizizen J. 1960. Requirements for transformation in *Bacillus subtilis*. J Bacteriol 81:741–746.

3. Lewis PJ, Thaker SD, Errington J. 2000. Compartmentalization of transcription and translation in *Bacillus subtilis*. EMBO J 19:710–718.

4. Johnson AS, van Horck S, Lewis PJ. 2004. Dynamic localization of membrane proteins in *Bacillus subtilis*. Microbiology 150:2815–2824.

5. Su'etsugu M, Errington J. 2011. The replicase sliding clamp dynamically accumulates behind progressing replication forks in *Bacillus subtilis* cells. Mol Cell 41:720–732.

6. Jahn N, Brantl S, Strahl H. 2015. Against the mainstream: the membrane-associated type I toxin BsrG from *Bacillus subtilis* interferes with cell envelope biosynthesis without increasing membrane permeability. Mol Microbiol 98:651–666.

7. Müller A, Wenzel M, Strahl H, Grein F, Saaki TN V, Kohl B, Siersma T, Bandow JE, Sahl H-G, Schneider T, Hamoen LW. 2016. Daptomycin inhibits cell envelope synthesis by interfering with fluid membrane microdomains. Proc Natl Acad Sci U S A 113:E7077–E7086.

8. Eggert T, van Pouderoyen G, Dijkstra BW, Jaeger KE. 2001. Lipolytic enzymes LipA and LipB from *Bacillus subtilis* differ in regulation of gene expression, biochemical properties, and three-dimensional structure. FEBS Lett 502:89–92.

9. Saeloh D, Tipmanee V, Jim KK, Dekker MP, Bitter W, Voravuthikunchai SP, Wenzel M, Hamoen LW. 2018. The novel antibiotic rhodomyrtone traps membrane proteins in vesicles with increased fluidity. PLoS Pathog 14:e1006876.

10. Avery OT, Macleod CM, McCarty M. 1944. Studies on the chemical nature of the substane inducing transformation of pneumococcal types: Induction of transformation by a desoxyribonucleic acid fraction isolated from *Pneumococcus* type III. J Exp Med 79:137–158.

11. Datsenko KA, Wanner BL. 2000. One-step inactivation of chromosomal genes in *Escherichia coli* K-12 using PCR products. Proc Natl Acad Sci U S A 97.

12. Yang X, Lyu Z, Miguel A, McQuillen R, Huang KC, Xiao J. 2017. GTPase activity-coupled treadmilling of the bacterial tubulin FtsZ organizes septal cell wall synthesis. Science 355:744–747.

13. Ferenci T, Zhou Z, Betteridge T, Ren Y, Liu Y, Feng L, Reeves PR, Wang L. 2009. Genomic sequencing reveals regulatory mutations and recombinational events in the widely used MC4100 lineage of *Escherichia coli* K-12. J Bacteriol 191:4025–4029.

14. Rowland SL, Fu X, Sayed MA, Zhang Y, Cook WR, Rothfield LI. 2000. Membrane redistribution of the *Escherichia coli* MinD protein induced by MinE. J Bacteriol 182:613–619.

15. Steenhuis M, Ten Hagen-Jongman CM, van Ulsen P, Luirink J. 2020. Stress-based

high-throughput screening assays to identify inhibitors of cell envelope biogenesis. Antibiot (Basel, Switzerland) 9:808.

16. Skoczinski P, Volkenborn K, Fulton A, Bhadauriya A, Nutschel C, Gohlke H, Knapp A, Jaeger K-E. 2017. Contribution of single amino acid and codon substitutions to the production and secretion of a lipase by *Bacillus subtilis*. Microb Cell Fact 16:160.

17. Henriques G, McGovern S, Neef J, Antelo-Varela M, Götz F, Otto A, Becher D, van Dijl JM, Jules M, Delumeau O. 2020. SppI forms a membrane protein complex with SppA and inhibits its protease activity in *Bacillus subtilis*. mSphere 5.

18. Shih Y-L, Le T, Rothfield L. 2003. Division site selection in *Escherichia coli* involves dynamic redistribution of Min proteins within coiled structures that extend between the two cell poles. Proc Natl Acad Sci U S A 100:7865–7870.
